# Scalable synthesis and coupling of quaternary α-arylated amino acids: α-aryl substituents are tolerated in α-helical peptides†

**DOI:** 10.1039/d1sc01378e

**Published:** 2021-06-09

**Authors:** Daniel J. Leonard, Francis Zieleniewski, Isabelle Wellhöfer, Emily G. Baker, John W. Ward, Derek N. Woolfson, Jonathan Clayden

**Affiliations:** School of Chemistry, University of Bristol Cantock's Close Bristol BS8 1TS UK D.N.Woolfson@bristol.ac.uk j.clayden@bristol.ac.uk; School of Biochemistry, University of Bristol, Medical Sciences Building, University Walk Bristol BS8 1TS UK; Bristol BioDesign Institute, University of Bristol, Life Sciences Building Tyndall Avenue Bristol BS8 1TQ UK

## Abstract

Quaternary amino acids are important tools for the modification and stabilisation of peptide secondary structures. Here we describe a practical and scalable synthesis applicable to quaternary alpha-arylated amino acids (Q4As), and the development of solid-phase synthesis conditions for their incorporation into peptides. Monomeric and dimeric α-helical peptides are synthesised with varying degrees of Q4A substitution and their structures examined using biophysical methods. Both enantiomers of the Q4As are tolerated in folded monomeric and oligomeric α-helical peptides, with the (*R*)-enantiomer slightly more so than the (*S*).

## Abstract 2

Both *R* and *S* enantiomers of Fmoc-protected amino acids bearing α-aryl substituents may be made on gram scale. Solid-phase synthesis leads to helical peptides unperturbed by the presence of these additional α-aryl groups.

## Graphical abstract

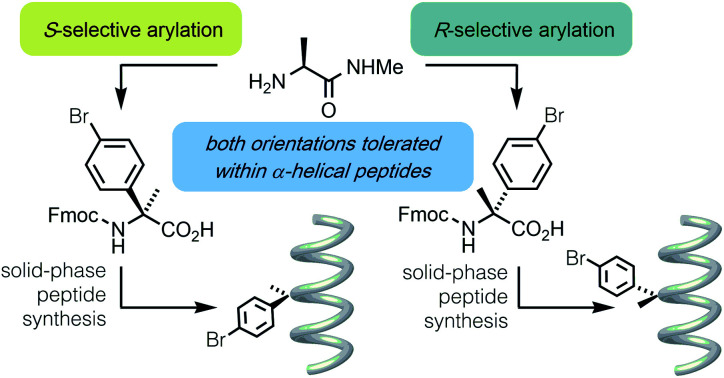

## Introduction

Advances in automated synthesis and better understanding of peptide and peptidomimetic structural properties have allowed rapid development in the field of peptide-based therapeutics. To date, over 60 peptidic drugs have been brought to market, with a further 400 potential therapeutics currently under evaluation in clinical or pre-clinical trials.^1^

The use of peptides as active pharmaceutical compounds has advantages. Such molecules can exhibit high biological activity, low toxicity and excellent specificity. However, there are concerns that peptidic drugs lack oral bioavailability and have poor stability under physiological conditions. Accordingly, the development of chemical methods to stabilise the bioactive states of peptides whilst maintaining activity and improving bioavailability is an active area of research.^2^ The incorporation of unnatural amino acids is a key aspect of this work. In particular, quaternary amino acids have been at the forefront of research in this area, being widely employed in the synthesis of bioactive peptides and peptidomimetics due to their resistance to racemisation and higher metabolic stability.^3^ Substitution at the C_α_ carbon of α-amino acids can alter steric features sufficiently to influence conformations adopted by peptides, which plays a major role in determining their biological activities.^4^ α-Arylated amino acids do not occur naturally and their incorporation into peptide structures could allow exploration of new chemical space beyond that tested through traditional peptide design. In addition, whilst phenylglycine and related residues are prone to racemisation during Fmoc solid-phase peptide synthesis (SPPS),^5^ quaternary α-arylated amino acids, which lack an enolisable proton, are fully configurationally stable.

## Results and discussion

### Gram-scale synthesis of the Q4As

**Scheme 1:**
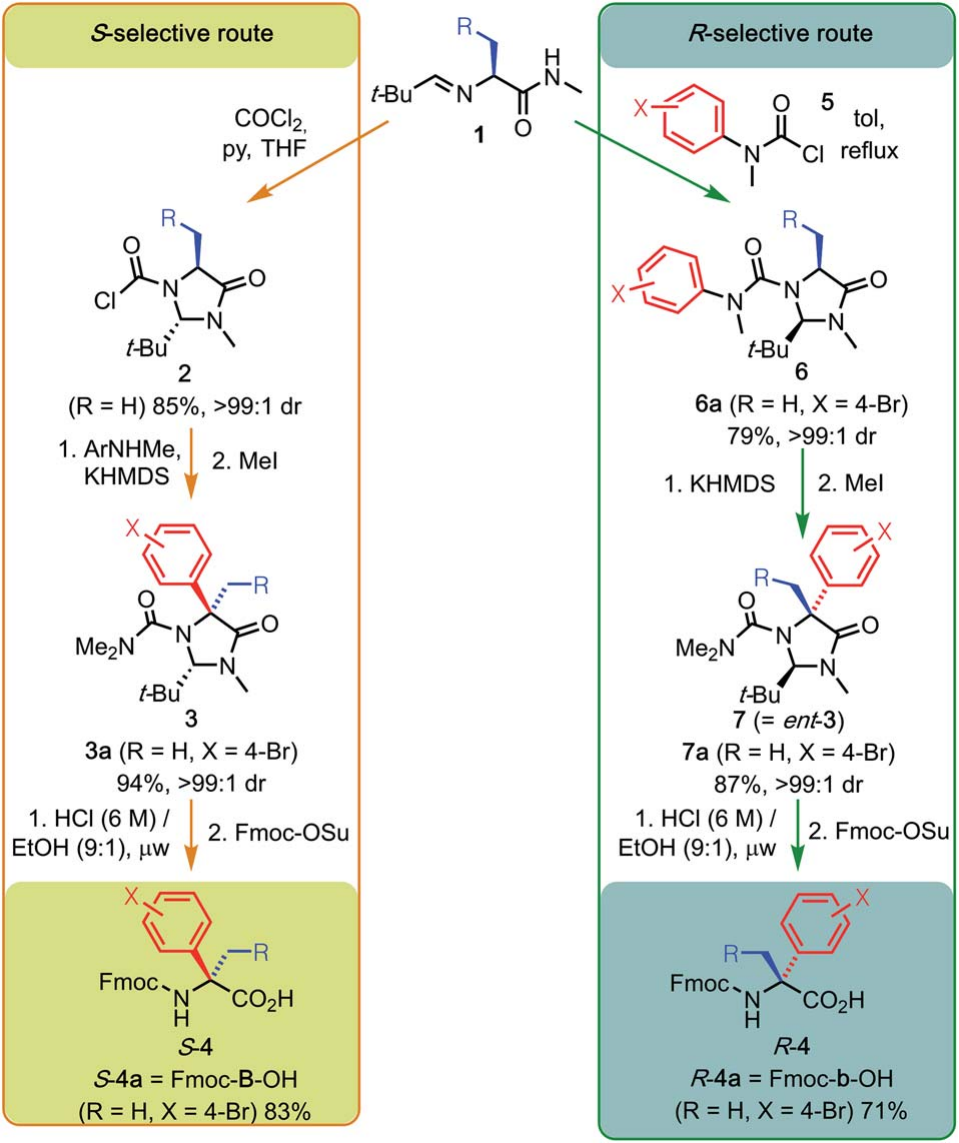
Synthetic pathways from l-alanine to Fmoc-protected *S* and *R*-(α-4-bromophenyl)alanine **4a** by way of *N*-chloroformylimidazolidinone **2a** or *N*′-bromophenyl imidazolinyl urea **6a**. The sequence shown by the orange arrows starting from **1** constitutes a general *S*-selective route to quaternary alpha-arylated amino acids (Q4As) *S*-**4**, whereas the sequence shown by the green arrows constitutes an *R*-selective route to Q4As *R*-**4**. KHMDS = potassium hexamethyldisilazide.

Previously, we reported a general and stereodivergent synthesis of quaternary alpha-arylated amino acids (Q4As).^6^ This allows the preparation of both product enantiomers by arylation of the same tertiary precursor depending on the choice of route. In order to test the suitability of Q4As for automated SPPS, larger quantities of Fmoc-protected products were required, and so we optimised the method further, focussing on overall yield and scalability. Our principal target was a multi-gram preparation of both *S*- and *R*-(α-4-bromophenyl)alanine **4a** (Scheme 1), for which we use the single letter codes **B** and **b**. These amino acids provide a representative example of the compound class, and the bromophenyl substituent provides a potential synthetic handle for functionalisation after incorporation into the peptide chain.

Amidation of commercial l-alanine methyl ester hydrochloride and treatment with pivaldehyde gave the starting material for both routes, imine **1** (R = H). Since our original publication,^6^ we have optimised the method for diastereoselective formation of *trans N*-chloroformylimidazolidinones.^7^ In this way, 40 g of **2** (R = H) were readily prepared as a single diastereoisomer (our previously reported method gave a mixture of **2** and its *cis* isomer). Sequential addition of KHMDS to a solution of **2** and 4-bromo-*N*-methylaniline (the formal equivalent of the aryl electrophile) effected one-pot tandem urea formation and diastereoselective N to C migration of the aryl ring to install the quaternary centre. Quenching with methyl iodide gave the protected *N*,*N*-dimethyl urea **3a**, a step that proved critical for avoiding the formation of hydantoin by-products in the subsequent hydrolysis.^6^ This is an operationally simple, one-pot procedure. It combines urea formation, deprotonation, rearrangement and protection to give the penultimate species in the synthesis in a remarkable 94% yield. This is with complete diastereoselectivity and without the need for intermediate chromatographic purification.

In a complementary manner, acylation of the same imine **1** with carbamoyl chloride **5** (X = 4-Br) in refluxing toluene resulted in selective cyclisation to the *cis* imidazolidinone, yielding urea **6a**. Diastereoselective N to C migration of the bromophenyl moiety was induced by enolisation of **6a** with KHMDS, generating the enantiomeric α-quaternary product **7a** in high yield after methylation.

Removal of the imidazolidinone motif from **3a** and **7a** to reveal the quaternary amino acid was achieved by acidic hydrolysis in 3 hours using microwave irradiation. Unlike previous methods, which involve laborious prior separation from by-products of the free amino acid by ion-exchange chromatography,^6^*in situ* Fmoc protection allowed facile isolation of both *S*-**4a** and *R*-**4a** in multigram quantities.

We expected this method to be generalisable to the gram-scale synthesis of other Q4As, and to demonstrate its scope we synthesised the further series of functionalised Q4A precursors **3** and used them to prepare Fmoc-protected Q4As **4**, as shown in Table 1. Coupling and rearrangement was unaffected by the steric bulk of the aromatic amino acids: Phe and *O-*benzyl Tyr, and even multiply halogenated targets were formed cleanly. Samples of (*S*)-**4** were isolated in quantities from 0.2–1.8 g.

**Table 1:** Synthesis of Fmoc-protected Q4As *via* imidazolidinones **3**

R=	X=	**3**, yield	(*S*)-**4**, yield/% (scale)
H	4-Br	**3a**, 94	**4a**, 83 (1.8 g)
H	3-Br	**3b**, 91	**4b**, 50 (1.5 g)
H	3-CN	**3c**, 83	
H	3,5-F_2_	**3d**, 91	**4d**, 67 (0.5 g)
H	3,5-Cl_2_	**3e**, 90	
Ph	4-Cl	**3f**, 89	**4f**, 51 (0.2 g)
Ph	4-Me	**3g**, 80	**4g**, 62 (0.4 g)
Ph	3-Br	**3h**, 95	**4h**, 50 (0.4 g)
4-BnOC_6_H_4_	4-Me	**3i**, 88	
4-BnOC_6_H_4_	3-F, 4-Br	**3j**, 93	

### Incorporation of Q4As into peptides

The Fmoc protection achieved by this method enabled direct use of these amino acids in solid-phase peptide synthesis (SPPS). The α-aryl substituents of these quaternary amino acids replace the C_α_–H bonds of their proteinogenic counterparts, offering the possibility of incorporating additional functionality into peptide mimetics without otherwise altering the side chains of the parents. Previous approaches to the incorporation of functionalised quaternary residues have offered much less subtle control: for example, the use of derivatives of achiral 4-aminopiperidine-4-carboxylic acid makes it impossible for the natural side chains to be present.^8^ Thus, we aimed to explore the extent of the effect on secondary structure of inserting the *p*-bromophenyl-substituted quaternary residues **B** and **b** as ‘point mutations’ in peptides of established conformational preference.

The initial challenge was how to incorporate the sterically demanding α-arylated residues **B** (of which *S*-**4** is the Fmoc-protected precursor) and **b** (of which *R*-**4** is the Fmoc protected precursor) into peptide sequences using SPPS. Previously, we had introduced related residues into Aib-based helices by solution-phase methods,^9^ but we sought an efficient and reliable method for automated peptide coupling with Q4As.^10^ Other work has demonstrated efficient coupling of multiple consecutive α,α-dimethylated aminoisobutyric acid (Aib) residues by SPPS in the presence of Oxyma.^11^ We found that applying these same coupling cycles allowed α-arylated amino acids *S*-**4** and *R*-**4** to be coupled to proteinogenic residues.

### Conformational effects of Q4As on peptide secondary structure

**Fig. 1:**
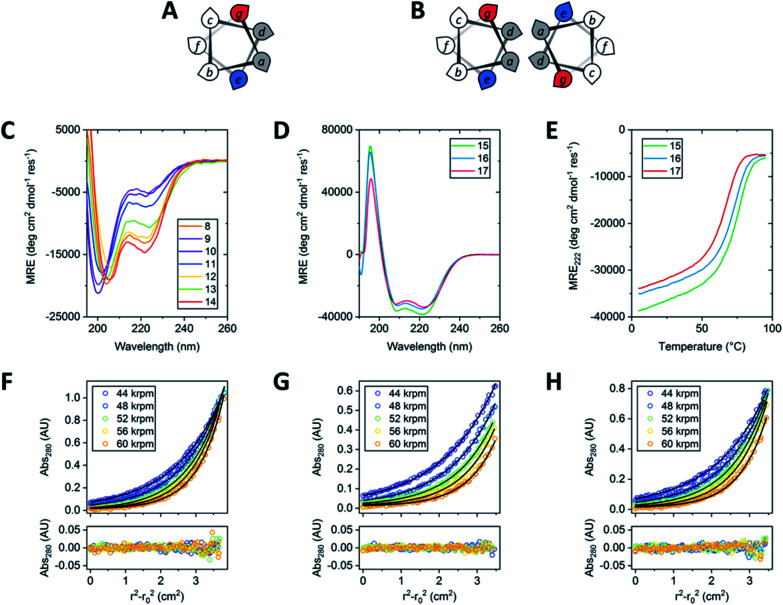
Helical-wheel representations of CC-Mono (A) and CC-Di (B). (C) CD spectra of peptides 8–14. (D) CD spectra of peptides 15–17. (E) Variable temperature CD measurements for peptides 15–17 monitoring MRE_222_ from 5 to 95 °C. All CD measurements were carried out with 50 μM peptide in phosphate-buffered saline (PBS, pH 7.4). (F–H) Sedimentation equilibrium profiles for peptides 15–17 respectively. Top: SE data (circles) fitted to a single-ideal species model (black lines). Bottom: residuals for the above fits. SE measurements were carried out at 20 °C in PBS, pH 7.4 at 37 μM for peptide 15 and 75 μM for peptides 16 & 17.

To determine the consequences of introducing a Q4A residue into regular secondary structures,^12^ we synthesised a series of *de novo* designed peptides (Table 2) based on a monomeric α helix, CC-Mono, and a dimeric α-helical coiled coil, CC-Di (Fig. 1A and B).^13^ CC-Mono (**8**) has the 7-residue (heptad) sequence repeat, *abcdefg*, of coiled-coil peptides that normally oligomerise. However, in CC-Mono, the *a* and *d* positions are occupied by Ala residues, which are too small to drive the association of helices and the formation of a consolidated *a*/*d-*based hydrophobic core typical of coiled-coil oligomers. The parent CC-Mono is 35% α helical by circular dichroism (CD) spectroscopy, which we argued would be highly sensitive to single point mutations to quaternary amino acids should these affect the local and global conformations of the peptides.

**Table 2:** Designed peptides and summary of biophysical data^a^

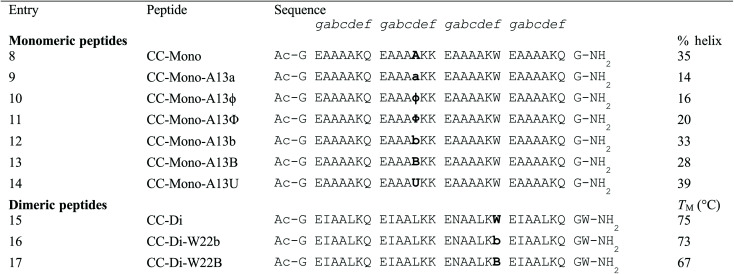

a Letters are standard one-letter amino acid codes, plus **B**/**b**: *S*/*R*-α-(4-bromophenyl)alanine; **Φ**/**ϕ**: l/d-phenylglycine; **U**: α-aminoisobutyric acid (Aib).

Variants of CC-Mono were synthesised in which a single central Ala residue at the *d* position of the second heptad was mutated: peptide **9** had d-Ala (**a**) at this site as a control; peptides **10** and **11** introduced the enantiomers of phenylglycine (d-PhG, **ϕ** and l-PhG, **Φ**) as tertiary analogues of the α-bromophenyl amino acids **b** and **B**, respectively (peptides **12** and **13**). Peptide **14** had the smaller, but still helix-inducing, Aib (**U**) as a further control.

The control peptides (**9** and **14**) responded as predicted: in **9**, with the d-Ala mutation, the helicity measured by CD spectroscopy was reduced by >2 fold (Fig. 1C and Table 2); whereas the substitution with Aib (**14**), known for its high helical propensity,^15^ marginally increased the helicity up to 39%. The incorporation of either enantiomer of phenylglycine (**10** and **11**) into CC-Mono reduced the overall helicity of the system in a similar manner to introduction of d-Ala (Table 2 and Fig. 1C). By contrast, both Q4A substitutions **b** and **B** (peptides **12** and **13**, respectively) were remarkably well accommodated, and only marginally less folded than the parent peptide. Intriguingly, peptide **12** was slightly more folded (33%) than **13** (28%). This suggests that **b**—in which the Me group, rather than the Ar group, is orientated in the same direction as the methyl group in l-Ala—is better tolerated than **B**. α,α-Diarylated amino acids are incompatible with peptide helices.^14^ Therefore, the Q4A residues appear to occupy a ‘sweet spot’ as functionalisable replacements for l α-amino acids in α-helical structures.

Given this evidence that α-arylated amino acids are tolerated in α helices, we explored the insertion of the bromophenyl residues **b** and **B** into a cooperatively folded, oligomerising α-helical system. Specifically, we used the dimeric *de novo* designed coiled-coil peptide, CC-Di (Fig. 1B).^13^ In this peptide, Ile and Leu predominate at the *a* and *d* positions, respectively, of the sequence repeat leading to formation of an amphipathic helix that specifies and stabilises the dimer. The Asn-17, an *a* site, further specifies the dimer by disfavouring the alternative trimeric state. We wanted to test the impact of Q4A residues on such systems, but without compromising the dimer interface. Therefore, we made point mutations by replacing the single Trp at the third *f* position, as this is furthest from the helix–helix interface, Fig. 1B. In addition to CC-Di, peptide **15**, as a control, peptides **16** and **17** were synthesised with the Trp mutated to **b** and **B**, respectively. In each case an additional Trp chromophore was added at the *C* terminus to allow peptide concentration to be determined (Table 2).

As with the monomeric system, by CD spectroscopy, both quaternary α-arylated residues **b** and **B** were well tolerated in the dimeric assembly (Fig. 1D). The α-arylated peptides **16** and **17** showed only slight decreases in α helicity compared with the parent CC-Di (**15**). Moreover, and also similar to the parent, **16** and **17** had sigmoidal thermal unfolding transitions consistent with cooperatively folded and unique species (Fig. 1E). Sedimentation-equilibrium experiments by analytical ultracentrifugation showed that all three peptides formed dimers in solution with fitted molecular weights 2.13, 1.99 and 2.14 times the monomer masses, respectively (Fig. 1F and G). Once again, the (*R*)-enantiomer **b** (where the aryl group directly replaces the α-H) was marginally more favoured than **B**, as indicated by both the helicities from the CD spectra and the midpoints of the thermal unfolding curves (*T*_M_) (Table 2 and Fig. 1E). The high helical content, similar thermal unfolding transitions, and identical oligomerisation states of peptides **16** and **17** compared to the parent **15** indicate that over helical quaternary structures of three peptides are closely similar, with little evidence of disruption such as fraying after the point mutations. Previously, we have found that placing the dimer-specifying Asn at *a* sites of different heptads can lead to helix fraying, which is accompanied by changes in thermal unfolding transitions and oligomeric states.^16^ By contrast, both enantiomers of α-bromophenyl alanine are well tolerated within a fully folded and defined quaternary peptide structure.

## Conclusion

In conclusion, we have shown that both enantiomers of Fmoc-protected α-aryl alanine derivatives can be prepared on a multi-gram scale from the commercial l-alanine methyl ester hydrochloride precursor to **1**. Furthermore, these amino acids, represented by l- and d-α*-*(bromophenyl) alanine, can be incorporated into peptide structures by SPPS, and both enantiomers are well tolerated when they replace l-amino acids in α-helical peptide structures. Future work will incorporate Q4As for more sterically demanding amino acids such as leucine and seek to further exploit the functionalization of these additional ‘conformationally silent’ aryl substituents.

## Data availability

The datasets supporting this article have been uploaded as part of the supplementary material.

## Author contributions

DNW and JPC conceived and directed the project. DJL, FZ, IW, EGB, and JWW planned and carried out experimental work. FZ, DJL, DNW and JPC wrote the manuscript.

## Conflicts of interest

There are no conflicts to declare.

## Supplementary Material

SC-012-D1SC01378E-s001
