# Supplementary material for: Scalable synthesis and coupling of quaternary α-arylated amino acids: α-aryl substituents are tolerated in α-helical peptides
Source: Chem Sci. 2021 Jun 9;12(27):9386–90. doi: 10.1039/d1sc01378e (PMC8278958; doi:10.1039/d1sc01378e)
Supplement: SC-012-D1SC01378E-s001 [file SC-012-D1SC01378E-s001.pdf]

Electronic Supplementary Material (ESI) for Chemical Science.
This journal is © The Royal Society of Chemistry 2021

Scalable Synthesis of Unnatural α-Arylated Amino Acids and their Incorporation into Peptides using SPPS

Daniel J. Leonard,‡[a] Francis Zieleniewski,‡[a] Isabelle Wellhöfer,[a] Emily G. Baker,[a] John W. Ward,[a] Derek N. Woolfson*[a,b,c] and Jonathan Clayden*[a]

[a.] School of Chemistry, University of Bristol, Cantock's Close, Bristol, BS8 1TS, U.K.

[b.] School of Biochemistry, University of Bristol, Medical Sciences Building, University Walk, Bristol, BS8 1TS, U.K.

[c.] Bristol BioDesign Institute, University of Bristol, Life Sciences Building, Tyndall Avenue, Bristol BS8 1TQ, U.K.

j.clayden@bristol.ac.uk

d.n.woolfson@bristol.ac.uk

Supporting Information

Contents

General Information S2
Experimental Procedures and Characterisation S2
^{1}H and ^{13}C NMR Spectra S17
Peptide Synthesis S34
Peptide Purification S35
Peptide Concentration Determination S35
Circular Dichroism Spectroscopy S36
Analytical Ultracentrifugation S36
Peptide Characterisation S37
Sedimentation Equilibrium Traces S47
References S49

General Information

All reactions were performed under a nitrogen atmosphere in flame-dried apparatus, unless stated otherwise. All reagents and chemicals were obtained from chemical suppliers and used without further purification, with the exception of those listed below. DCM, THF, Et_2O and toluene were collected under argon from an Innovative Technologies PureSolve PS-MP-5 solvent purification system. Et_3N was stored over KOH pellets and used directly. Pet.Ether refers to the fractions of petroleum ether boiling between 40 – 60 °C. Acetone/dry ice cooling baths were used to maintain −78 °C. Thin-layer chromatography (TLC) was performed using pre-coated plates (Macherey-Nagel Polygram SIL G/UV254). Visualisation was achieved by way of UV light (at 254 nm), and either potassium permanganate or 'Seebach' stains (2.50 g phosphomolybdic acid hydrate, 1.00 g $Ce(SO_4)_2·4H_2O$, 3.00 mL conc. H_2SO_4, 90.00 mL water). Flash column chromatography (FCC) was carried out using an automated Biotage® Isolera Spektra Four with gradient elution on pre-packed silica gel Biotage® SNAP Ultra columns or Biotage® Sfär C18 columns for reversed phase (RP), with compounds loaded as saturated solutions. Nuclear Magnetic Resonance (NMR) spectra (^{1}H NMR and ^{13}C NMR) were recorded on either were recorded on Jeol ECS (400 MHz), Varian VNMR (400 MHz or 500 MHz) or Bruker Ultrashield (400 MHz or 500 MHz) spectrometers. Chemical shifts (δ) are quoted in parts per million (ppm) downfield of trimethylsilane. Spectra were calibrated using the residual solvent peaks for $CDCl_3$ (δ_H: 7.26 ppm; δ_C: 77.16 ppm) and $(CD_3)_2SO$ (δ_H: 2.50 ppm; δ_C: 39.52 ppm) as appropriate. Coupling constants (*J*) are quoted in Hz and are rounded to the nearest 0.1 Hz. Splitting patterns are abbreviated to: singlet (s), doublet (d), triplet (t), quartet (q), multiplet (m), broad (br.) or some combination thereof. Major and minor rotameric peaks are denoted by the subscripts $_{(maj)}$ and $_{(min)}$. High resolution mass spectra (HRMS) were recorded by technical staff at the University of Bristol on a Thermo Scientific Orbitrap Elite or Bruker Daltonics MicrOTOF II. Optical rotations ($[\alpha]_D^T$) were recorded on a Bellingham & Stanley ADP220 polarimeter and are quoted in g^{-1} mL^{-1} dm^{-1} with the temperature, solvent and concentration (g/100 mL) stated.

Experimental Procedures and Characterisation

4-Bromo-*N*-methylaniline (S1)

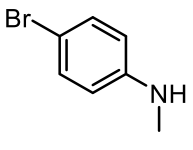

4-Bromoaniline (10.0 g, 58.1 mmol, 1.0 eq.) and paraformaldehyde (2.62 g, 87.2 mmol, 1.5 eq.) were dissolved in MeOH (120 mL, 0.5 M). NaOMe (15.7 g, 291 mmol, 5.0 eq.) was added and the reaction mixture was refluxed for 2 h. The mixture was cooled to 0 °C before $NaBH_4$ (3.30 g, 87.2 mmol, 1.5 eq.) was added in three portions. The mixture was refluxed for a further 1 h. After cooling to room temperature, the mixture was concentrated *in vacuo* before the residue was redissolved in DCM and washed with H_2O. The organic layer was dried over

Na_2SO_4, filtered and concentrated *in vacuo*. Purification by automated FCC (2%→20% EtOAc in Pet.Ether) gave the title compound (7.89 g, 42.4 mmol, 73%) as a brown liquid. **S1:** **R_f** = 0.26 (9:1 Pet.Ether:EtOAc). **^{1}H NMR** (400 MHz, $CDCl_3$): δ_H = 7.29 – 7.23 (m, 2H, CH_{Ar}), 6.51 – 6.45 (m, 2H, CH_{Ar}), 3.72 (br. s, 1H, N*H*), 2.81 (s, 3H, NHCH_3). Data in agreement with reported values.[1]

(4-Bromophenyl)(methyl)carbamic chloride (S2)

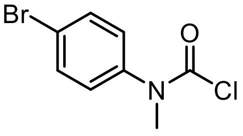

To a solution of triphosgene (3.19 g, 10.8 mmol, 0.50 eq.) in anhydrous DCM (54 mL, 0.4 M) at −78 °C, was added pyridine (1.73 mL, 1.70 g, 21.5 mmol, 1.00 eq.), followed by a solution of aniline **S1** (4.00 g, 21.5 mmol, 1.00 eq.) in anhydrous DCM (1.30 mL, 16.0 M). After 10 min, reaction mixture was allowed to warm to room temperature and left to stir for 3 h before quenching with HCl (20.0 mL, 1.0 M, aq.). The organic layer was separated and the aqueous layer extracted with DCM (3×20.0 mL). The combined organic layers were washed with $NaHCO_3$ (50.0 mL, sat. aq.), dried over $MgSO_4$, filtered and concentrated *in vacuo* to give the title compound (5.34 g, 21.5 mmol, >99%) as an orange solid. **S2:** **R_f** = 0.34 (9:1 Pet.Ether:EtOAc). **^{1}H NMR** (400 MHz, $CDCl_3$): δ_H = 7.56 (d, *J* = 8.6, 2H, CH_{Ar}), 7.14 (d, *J* = 8.2, 2H, CH_{Ar}), 3.36 (s, 3H, NHCH_3). Data in agreement with reported values.[2]

(*S*)-2-((2,2-Dimethylpropylidene)amino)-*N*-methylpropanamide (S3)

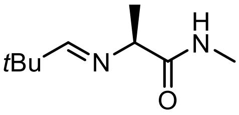

To L-alanine methyl ester hydrochloride (32.0 g, 0.229 mol, 1.0 eq.) was added $MeNH_2$ (33% wt. in EtOH, 200 mL, 1.605 mol, 7.0 eq.). The mixture was left to stir at room temperature for 48 h before being concentrated *in vacuo*. The crude *N*-methylamide hydrochloride was suspended in DCM (150 mL, 1.5 M) before Et_3N (48.0 mL, 0.344 mol, 1.5 eq.), $MgSO_4$ (28.0 g, 0.229 mol, 1.0 eq.) and pivaldehyde (27.4 mL, 0.252 mol, 1.1 eq.) were added. The mixture was left to stir at room temperature for 16 h before being filtered and concentrated *in vacuo*. The crude imine was redissolved in THF and filtered to remove any remaining Et_3N.HCl, before being concentrated *in vacuo* to give the title compound (34.6 g, 0.203 mol, 89%) as a pale yellow oil. **S3: ^{1}H NMR** (400 MHz, $CDCl_3$): δ_H = 7.51 (s, 1H, C*H*C(CH_3)$_3$), 6.91 (br. s, 1H, N*H*), 3.68 (q, *J* = 7.0, 1H, C*H*CH_3), 2.84 (d, *J* = 4.9, 3H, NHCH_3), 1.31 (d, *J* = 7.1, 3H, CHCH_3), 1.07 (s, 9H, C(CH_3)$_3$). Data in agreement with reported values.[3]

(*S*)-2-Amino-*N*-methyl-3-phenylpropanamide (S4)

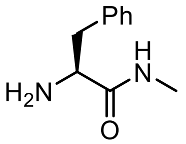

To L-phenylalanine ethyl ester hydrochloride (7.75 g, 33.8 mmol, 1.0 eq.) was added $MeNH_2$ (33% wt. in EtOH, 29.4 mL, 236 mmol, 7.0 eq.). The mixture was left to stir at room temperature for 48 h before being concentrated *in vacuo*, redissolved in $CHCl_3$ and washed with K_2CO_3 (3.8 M, aq.). The organic layer was separated and the aqueous layer

extracted with $CHCl_3$ (3×). The combined organic layers were dried over $MgSO_4$, filtered and concentrated *in vacuo* to give the title compound (5.90 g, 33.1 mmol, 98%) as a white solid. Data in agreement with reported values.[3]

(*S*)-2-((2,2-Dimethylpropylidene)amino)-*N*-methyl-3-phenylpropanamide (S5)

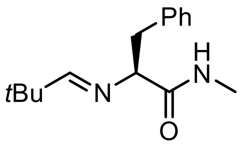

N-Methylamide **S4** (5.90 g, 33.1 mmol, 1.0 eq.) was dissolved in anhydrous DCM (44.0 mL, 0.75 M) before $MgSO_4$ (3.98 g, 33.1 mmol, 1.0 eq.) and pivaldehyde (4.30 mL, 39.7 mmol, 1.2 eq.) were added. The mixture was left to stir at room temperature for 16 h before being filtered and concentrated *in vacuo* to give the title compound (8.15 g, 33.1 mmol, >99%) as a white solid. Data in agreement with reported values.[3]

(*S*)-2-Amino-3-(4-(benzyloxy)phenyl)-*N*-methylpropanamide (S6)

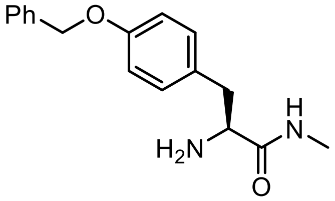

To L-Tyr(Bzl)-OMe·HCl (5.36 g, 16.7 mmol, 1.0 eq.) was added $MeNH_2$ (33% wt. in EtOH, 14.5 mL, 117 mmol, 7.0 eq.). The mixture was left to stir at room temperature for 48 h before being concentrated *in vacuo*, redissolved in $CHCl_3$ and washed with K_2CO_3 (3.8 M, aq.). The organic layer was separated and the aqueous layer extracted with $CHCl_3$ (3×). The combined organic layers were dried over $MgSO_4$, filtered and concentrated *in vacuo* to give the title compound (4.68 g, 16.5 mmol, 99%) as a white solid. Data in agreement with reported values.[4]

(*S*)-3-(4-(Benzyloxy)phenyl)-2-((2,2-dimethylpropylidene)amino)-*N*-methylpropanamide (S7)

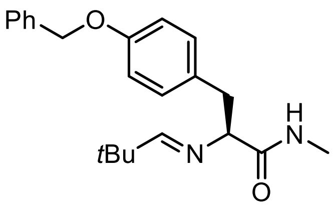

N-Methylamide **S6** (4.68 g, 16.5 mmol, 1.0 eq.) was dissolved in anhydrous DCM (40.0 mL, 0.4 M) before $MgSO_4$ (1.98 g, 16.5 mmol, 1.0 eq.) and pivaldehyde (2.15 mL, 19.7 mmol, 1.2 eq.) were added. The mixture was left to stir at room temperature for 16 h before being filtered and concentrated *in vacuo* to give the title compound (5.62 g, 15.9 mmol, 97%) as a white solid. Data in agreement with reported values.[4]

(2*S*,5*S*)-2-(*tert*-Butyl)-3,5-dimethyl-4-oxoimidazolidine-1-carbonyl chloride (S8)

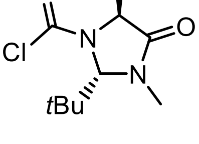

To a solution of imine **S3** (34.6 g, 0.203 mol, 1.0 eq.) in anhydrous THF (400 mL, 0.5 M) at 0 °C, was added, dropwise over ca. 30 mins, phosgene solution (20% wt. in toluene, 130 mL, 0.244 mol, 1.2 eq.). The reaction mixture was allowed to warm to room temperature and left to stir for 2 h. The mixture was cooled to 0 °C and pyridine (24.7 mL, 0.305 mol, 1.5 eq.) was added dropwise. The reaction was allowed to warm to room temperature and left to stir for a further 16 h before quenching with HCl (30 mL, 1 M, aq.). The mixture was concentrated

in vacuo, diluted in EtOAc and washed with HCl (3×, 1 M, aq.) and brine, dried over $MgSO_4$, filtered and concentrated *in vacuo*. The crude product (*>97:3 dr*) was filtered through a silica plug with Pet.Ether/EtOAc (3:1) and recrystallised from Pet.Ether/Et_2O (1:1) to give the title compound (40.2 g from two crops, 0.173 mol, 85%, >99:1 dr) as a white solid. **S8: R_f** = 0.27 (2:1 Pet.Ether:EtOAc); **^{1}H NMR** (400 MHz, $CDCl_3$) (0.56:0.44 rotamers): δ_H = 5.18 (br. s, 0.44H, C*H*C(CH$_3$)$_{3(min)}$), 5.13 (br. s, 0.56H, C*H*C(CH$_3$)$_{3(maj)}$), 4.15 (br. s, 1H, C*H*CH$_3$), 3.05 (s, 3H, NC*H*$_3$), 1.76 (br. s, 1.38H, CHC*H*$_{3(min)}$), 1.62 (br. s, 1.62H, CHC*H*$_{3(maj)}$), 1.06 (br. s, 5.19H, C(C*H*$_3$)$_{3(maj)}$), 1.01 (br. s, 3.81H, C(C*H*$_3$)$_{3(min)}$). Data in agreement with reported values.[3]

(2*S*,5*S*)-5-Benzyl-2-(*tert*-butyl)-3-methyl-4-oxoimidazolidine-1-carbonyl chloride (S9)

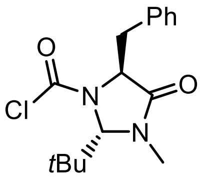

To a solution of imine **S5** (8.15 g, 33.1 mmol, 1.0 eq.) in anhydrous THF (66 mL, 0.5 M) at 0 °C, was added, dropwise over ca. 30 mins, phosgene solution (20% wt. in toluene, 26.0 mL, 49.6 mmol, 1.5 eq.). The reaction mixture was allowed to warm to room temperature and left to stir for 2 h. The mixture was cooled to 0 °C and pyridine (5.35 mL, 66.2 mmol, 2.0 eq.) was added dropwise. The reaction was allowed to warm to room temperature and left to stir for a further 16 h before quenching with HCl (1 M, aq.). The mixture was concentrated *in vacuo*, diluted in EtOAc and washed with HCl (3×, 1 M, aq.) and brine, dried over $MgSO_4$, filtered and concentrated *in vacuo*. The crude product was purified by automated FCC to give the title compound (8.67 g, 28.1 mmol, 85%, >99:1 dr) as a white solid. Data in agreement with reported values.[3]

(2*S*,5*S*)-5-(4-(Benzyloxy)benzyl)-2-(*tert*-butyl)-3-methyl-4-oxoimidazolidine-1-carbonyl chloride (S10)

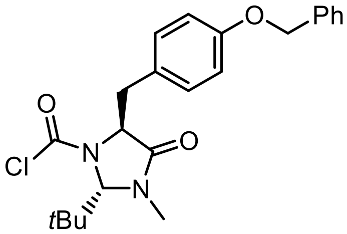

To a solution of imine **S7** (5.80 g, 16.5 mmol, 1.0 eq.) in anhydrous THF (33 mL, 0.5 M) at 0 °C, was added, dropwise over ca. 30 mins, phosgene solution (20% wt. in toluene, 13.2 mL, 24.7 mmol, 1.5 eq.). The reaction mixture was allowed to warm to room temperature and left to stir for 2 h. The mixture was cooled to 0 °C and pyridine (2.70 mL, 32.9 mmol, 2.0 eq.) was added dropwise. The reaction was allowed to warm to room temperature and left to stir for a further 16 h before quenching with HCl (1 M, aq.). The mixture was concentrated *in vacuo*, diluted in EtOAc and washed with HCl (3×, 1 M, aq.) and brine, dried over $MgSO_4$, filtered and concentrated *in vacuo*. The crude product was purified by automated FCC to give the title compound (5.31 g, 12.8 mmol, 78 %, >99:1 dr) as a white solid. Data in agreement with reported values.[3]

(2*R*,5*S*)-*N*-(4-Bromophenyl)-2-(*tert*-butyl)-*N*,3,5-trimethyl-4-oxoimidazolidine-1-carboxamide (6a)

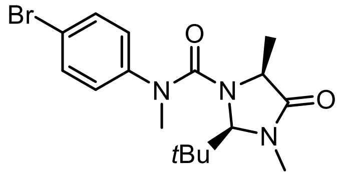

Imine **S3** (3.20 g, 18.8 mmol, 1.00 eq.), carbamoyl chloride **S2** (5.60 g, 22.6 mmol, 1.2 eq.) and DMAP (115 mg, 0.94 mmol, 0.05 eq.) were dissolved in anhydrous toluene (95 mL, 0.2 M). The reaction mixture was stirred at reflux for 72 h before being cooled to room temperature and diluted with EtOAc. The mixture was washed with HCl (3×30 mL, 1.0 M, aq.), $NaHCO_3$ (3×30 mL, sat. aq.) and brine. The combined organic layers were dried over $MgSO_4$, filtered and concentrated *in vacuo*. The crude product was purified by automated FCC (4% MeOH in DCM) and recrystallised from pentane:EtOAc (20:1) to give the title compound (5.64 g, 14.7 mmol, 79%) as a white solid. **6a: R_f** = 0.24 (9:1 pentane:EtOAc); **^{1}H NMR** (400 MHz, $CDCl_3$): δ_H = 7.57 – 7.47 (m, 2H, CH_{Ar}), 7.11 – 7.01 (m, 2H, CH_{Ar}), 5.58 (s, 1H, C*H*C(CH_3)$_3$, 3.95 (q, *J* = 6.9, 1H, C*H*CH_3), 3.16 (s, 3H, NCH_3), 2.95 (s, 3H, NCH_3), 0.98 (s, 9H, C(CH_3)$_3$), 0.47 (d, *J* = 6.9, 3H, CHCH_3); **^{13}C {^{1}H} NMR** (100 MHz, $CDCl_3$): δ_C = 173.1 (*C*=O), 163.2 (*C*=O), 145.4 (C_{Ar}), 133.4 (2×CH_{Ar}), 129.0 (2×CH_{Ar}), 120.4 (C_{Ar}), 82.8 (*C*HC(CH_3)$_3$), 59.2 (*C*HCH_3), 41.5 (N*C*H_3), 36.8 (*C*(CH_3)$_3$), 31.6 (N*C*H_3), 27.0 (C(*C*H_3)$_3$), 17.8 (CH*C*H_3); **HRMS** (ESI$^+$): *m/z* calcd for $C_{17}H_{24}BrN_3O_2$ ([M+H]$^+$) 382.1125, found 382.1125.

(2*R*,5*R*)-5-(4-Bromophenyl)-2-(*tert*-butyl)-*N*,*N*,3,5-trimethyl-4-oxoimidazolidine-1-carboxamide (7a)

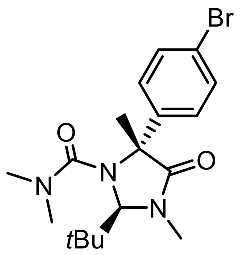

To a solution of urea **6a** (3.91 g, 9.87 mmol, 1.00 eq.) in anhydrous THF (100 mL, 0.1 M) at 0 °C was added dropwise KHMDS (1.0 M in THF, 14.8 mL, 14.8 mmol, 1.50 eq.). After 15 min, the reaction mixture was warmed to room temperature and allowed to stir for 3 h. MeI (2.46 mL, 39.5 mmol, 4.00 eq.) was added dropwise and the mixture was left to stir for a further 16 h. HCl (1 M, aq.) was added and, after dilution with EtOAc, the layers were separated. The organic layer was washed with HCl (1 M, aq., 2×), $Na_2S_2O_3$ (aq., 2×) and brine. The combined organic layers were dried over $MgSO_4$, filtered and concentrated *in vacuo.* The crude product was purified by automated FCC (1:1 Pet.Ether:EtOAc) to give the title compound (3.29 g, 8.61 mmol, 87%) as a white solid. **7a: R_f** = 0.32 (1:1 Pet.Ether:EtOAc); **^{1}H NMR** (400 MHz, $CDCl_3$): δ_H = 7.48 – 7.36 (m, 2H, CH_{Ar}), 7.12 – 7.01 (m, 2H, CH_{Ar}), 5.62 (s, 1H, C*H*C(CH_3)$_3$), 3.12 (s, 3H, NCH_3), 2.58 (br. s, 6H, N(CH_3)$_2$), 2.03 (s, 3H, CCH_3), 1.01 (s, 9H, C(CH_3)$_3$); **^{13}C {^{1}H} NMR** (100 MHz, $CDCl_3$): δ_C = 173.8 (*C*=O), 163.1 (*C*=O), 138.7 (C_{Ar}), 131.3 (2×CH_{Ar}), 128.1 (2×CH_{Ar}), 122.3 (C_{Ar}), 81.7 (*C*HC(CH_3)$_3$), 69.0 (*C*CH_3), 38.3 (N(*C*H_3)$_2$), 37.4 (*C*(CH_3)$_3$), 32.1 (N*C*H_3), 26.4 (C(*C*H_3)$_3$), 23.0 (C*C*H_3); **HRMS** (ESI$^+$): *m/z* calcd for $C_{18}H_{26}BrN_3O_2$ ([M+H]$^+$) 396.1281, found 396.1276.

(2*S*,5*S*)-5-(4-Bromophenyl)-2-(*tert*-butyl*)-N,N,3,5*-trimethyl-4-oxoimidazolidine-1-carboxamide (3a)

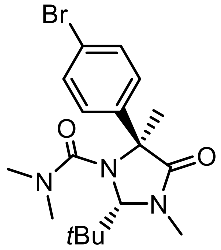

To a solution of *N*-chloroformylimidazolidinone **S8** (4.40 g, 18.9 mmol, 1.00 eq.) and aniline **S1** (3.87 g, 20.8 mmol, 1.10 eq.) in anhydrous THF (190 mL, 0.1 M) at −78 °C was added, dropwise, KHMDS (1.0 M in THF, 22.7 mL, 1.2 eq.). The reaction mixture was allowed to warm to 0 °C over ca. 2 h before additional KHMDS (1.0 M in THF, 22.7 mL, 1.2 eq.) was added dropwise. After 15 min, the mixture was allowed to warm to room temperature and stirred for a further 2 h. MeI (4.71 mL, 75.6 mmol, 4.00 eq.) was added dropwise and the mixture was left to stir for a further 16 h. HCl (1 M, aq.) was added and, after dilution with EtOAc, the layers were separated. The organic layer was washed with HCl (1 M, aq., 2×), $Na_2S_2O_3$ (aq., 2×) and brine. The combined organic layers were dried over $MgSO_4$, filtered and concentrated *in vacuo* to give the title compound (7.04 g, 17.8 mmol, 94%) as a pale yellow solid. **3a: HRMS** (ESI$^+$): *m/z* calcd for $C_{18}H_{26}BrN_3O_2$ ($[M+H]^+$) 396.1281, found 396.1275. For full data see compound **7a**.

(2*S*,5*S*)-5-(3-Bromophenyl)-2-(*tert*-butyl*)-N,N,3,5*-trimethyl-4-oxoimidazolidine-1-carboxamide (3b)

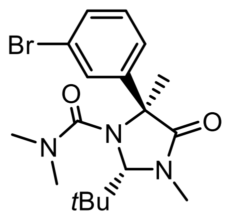

To a solution of *N*-chloroformylimidazolidinone **S8** (4.50 g, 19.3 mmol, 1.00 eq.) and 3-bromo-*N*-methylaniline (4.32 g, 23.2 mmol, 1.20 eq.) in anhydrous THF (130 mL, 0.15 M) at −78 °C was added, dropwise, KHMDS (1.0 M in THF, 29.0 mL, 1.5 eq.). The reaction mixture was allowed to warm to 0 °C over ca. 2 h before additional KHMDS (1.0 M in THF, 29.0 mL, 1.5 eq.) was added dropwise. After 15 min, the mixture was allowed to warm to room temperature and stirred for a further 2 h. MeI (4.80 mL, 77.1 mmol, 4.00 eq.) was added dropwise and the mixture was left to stir for a further 16 h. HCl (1 M, aq.) was added and, after dilution with EtOAc, the layers were separated. The organic layer was washed with HCl (1 M, aq., 2×), $Na_2S_2O_3$ (aq., 2×) and brine. The combined organic layers were dried over $MgSO_4$, filtered and concentrated *in vacuo* to give the title compound (6.94 g, 17.5 mmol, 91%) as an off white solid. **3b: ^{1}H NMR** (500 MHz, $CDCl_3$): δ_H = 7.34 – 7.28 (m, 2H, CH_{Ar}), 7.10 (t, *J* = 7.8 Hz, 1H, CH_{Ar}), 7.00 (d, *J* = 7.9 Hz, 1H, CH_{Ar}), 5.55 (s, 1H, C*H*C(CH_3)$_3$), 3.06 (s, 3H, NCH_3), 2.50 (br. s, 6H, N(CH_3)$_2$), 1.96 (s, 3H, CCH_3), 0.93 (s, 9H, C(CH_3)$_3$); **^{13}C {^{1}H} NMR** (125 MHz, $CDCl_3$): δ_C = 173.3 (*C*=O), 162.8 (*C*=O), 141.5 (C_{Ar}), 131.0 (CH_{Ar}), 129.5 (CH_{Ar}), 129.1 (CH_{Ar}), 125.1 (CH_{Ar}), 122.0 (C_{Ar}Br), 81.4 (*C*HC(CH_3)$_3$), 68.7 (*C*CH$_3$), 38.1 (N(*C*H$_3$)$_2$), 37.1 (*C*(CH_3)$_3$), 31.9 (N*C*H$_3$), 26.1 (C(*C*H$_3$)$_3$), 22.8 (C*C*H$_3$); **HRMS** (ESI$^+$): *m/z* calcd for $C_{18}H_{27}BrN_3O_2$ ($[M+H]^+$) 396.1281, found 396.1267.

(2*S*,5*S*)-2-(*tert*-Butyl)-5-(3-cyanophenyl)-*N*,*N*,3,5-trimethyl-4-oxoimidazolidine-1-carboxamide (3c)

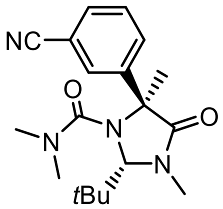

To a solution of *N*-chloroformylimidazolidinone **S8** (4.50 g, 19.3 mmol, 1.00 eq.) and 3-(methylamino)benzonitrile (3.07 g, 23.2 mmol, 1.20 eq.) in anhydrous THF (130 mL, 0.15 M) at −78 °C was added, dropwise, KHMDS (1.0 M in THF, 29.0 mL, 1.5 eq.). The reaction mixture was allowed to warm to 0 °C over ca. 2 h before additional KHMDS (1.0 M in THF, 29.0 mL, 1.5 eq.) was added dropwise. After 15 min, the mixture was allowed to warm to room temperature and stirred for a further 2 h. MeI (4.80 mL, 77.1 mmol, 4.00 eq.) was added dropwise and the mixture was left to stir for a further 16 h. HCl (1 M, aq.) was added and, after dilution with EtOAc, the layers were separated. The organic layer was washed with HCl (1 M, aq., 2×), $Na_2S_2O_3$ (aq., 2×) and brine. The combined organic layers were dried over $MgSO_4$, filtered and concentrated *in vacuo* to give the title compound (5.50 g, 16.1 mmol, 83%) as a white solid. **3c:** **^{1}H NMR** (400 MHz, $CDCl_3$): δ_H = 7.49 (s, 2H, CH_{Ar}), 7.38 – 7.28 (m, 2H, CH_{Ar}), 5.54 (s, 1H, C*H*C(CH_3)$_3$), 3.08 (s, 3H, NCH_3), 2.52 (br. s, 6H, N(CH_3)$_2$), 1.99 (s, 3H, CCH_3), 0.94 (s, 9H, C(CH_3)$_3$); **^{13}C {^{1}H} NMR** (125 MHz, $CDCl_3$): δ_C = 172.8 (*C*=O), 162.6 (*C*=O), 140.8 (C_{Ar}), 131.5 (CH_{Ar}), 130.9 (CH_{Ar}), 129.6 (CH_{Ar}), 128.8 (CH_{Ar}), 118.4 (*C*≡N), 112.0 (C_{Ar}CN), 81.5 (*C*HC(CH_3)$_3$), 68.4 (*C*CH$_3$), 38.2 (N(CH_3)$_2$), 37.1 (*C*(CH_3)$_3$), 31.8 (NCH_3), 26.0 (C(CH_3)$_3$), 22.6 (CCH_3); **HRMS** (ESI$^+$): *m/z* calcd for $C_{19}H_{27}N_4O_2$ ([M+H]$^+$) 343.2129, found 343.2128.

(2*S*,5*S*)-2-(*tert*-Butyl)-5-(3,5-difluorophenyl)-*N*,*N*,3,5-trimethyl-4-oxoimidazolidine-1-carboxamide (3d)

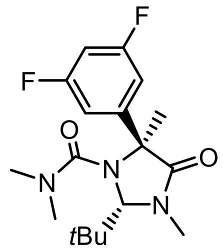

To a solution of *N*-chloroformylimidazolidinone **S8** (5.00 g, 21.5 mmol, 1.00 eq.) and 3,5-difluoro-*N*-methylaniline (3.38 g, 23.6 mmol, 1.10 eq.) in anhydrous THF (210 mL, 0.1 M) at −78 °C was added, dropwise, KHMDS (1.0 M in THF, 25.8 mL, 1.2 eq.). The reaction mixture was allowed to warm to 0 °C over ca. 2 h before additional KHMDS (1.0 M in THF, 25.8 mL, 1.2 eq.) was added dropwise. After 15 min, the mixture was allowed to warm to room temperature and stirred for a further 2 h. MeI (4.00 mL, 64.3 mmol, 3.00 eq.) was added dropwise and the mixture was left to stir for a further 16 h. HCl (1 M, aq.) was added and, after dilution with EtOAc, the layers were separated. The organic layer was washed with HCl (1 M, aq., 2×), $Na_2S_2O_3$ (aq., 2×) and brine. The combined organic layers were dried over $MgSO_4$, filtered and concentrated *in vacuo* to give the title compound (6.90 g, 19.5 mmol, 91%) as a white solid. **3d:** **^{1}H NMR** (500 MHz, $CDCl_3$): δ_H = 6.81 – 6.58 (m, 3H, CH_{Ar}), 5.60 (s, 1H, C*H*C(CH_3)$_3$), 3.10 (s, 3H, NCH_3), 2.60 (br. s, 6H, N(CH_3)$_2$), 1.99 (s, 3H, CCH_3), 0.98 (s, 9H, C(CH_3)$_3$); **^{13}C {^{1}H} NMR** (125 MHz, $CDCl_3$): δ_C = 173.0 (*C*=O), 162.9 (*C*=O), 162.7 (dd, $^1J_{C-F}$ = 248.8, 12.7, 2×C_{Ar}F), 143.7 (t, $^3J_{C-F}$ = 8.4, C_{Ar}), 109.8 – 109.6 (m, 2×CH_{Ar}), 103.7 (t, $^2J_{C-F}$ = 25.3, CH_{Ar}), 81.6 (*C*HC(CH_3)$_3$), 68.6 (*C*CH$_3$), 38.4 (N(CH_3)$_2$),

37.4 ($C(CH_3)_3$), 32.0 (NCH_3), 26.2 ($C(CH_3)_3$), 23.0 (CCH_3); **HRMS** (ESI+): *m/z* calcd for $C_{18}H_{25}F_2N_3O_2Na$ ($[M+Na]^+$) 376.1807 found 376.1802.

(2*S*,5*S*)-2-(*tert*-Butyl)-5-(3,5-dichlorophenyl)-*N*,*N*,3,5-trimethyl-4-oxoimidazolidine-1-carboxamide (3e)

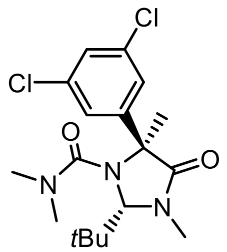

To a solution of *N*-chloroformylimidazolidinone **S8** (5.00 g, 21.5 mmol, 1.00 eq.) and 3,5-dichloro-*N*-methylaniline (4.16 g, 23.6 mmol, 1.10 eq.) in anhydrous THF (210 mL, 0.1 M) at −78 °C was added, dropwise, KHMDS (1.0 M in THF, 25.8 mL, 1.2 eq.). The reaction mixture was allowed to warm to 0 °C over ca. 2 h before additional KHMDS (1.0 M in THF, 25.8 mL, 1.2 eq.) was added dropwise. After 15 min, the mixture was allowed to warm to room temperature and stirred for a further 2 h. MeI (4.00 mL, 64.3 mmol, 3.00 eq.) was added dropwise and the mixture was left to stir for a further 16 h. HCl (1 M, aq.) was added and, after dilution with EtOAc, the layers were separated. The organic layer was washed with HCl (1 M, aq., 2×), $Na_2S_2O_3$ (aq., 2×) and brine. The combined organic layers were dried over $MgSO_4$, filtered and concentrated *in vacuo* to give the title compound (7.46 g, 19.3 mmol, 90%) as an off white solid. **3e: ^{1}H NMR** (400 MHz, $CDCl_3$): δ_H = 7.25 (t, *J* = 1.8, 1H, CH_{Ar}), 7.06 (d, *J* = 1.9, 2H, CH_{Ar}), 5.60 (s, 1H, $CHC(CH_3)_3$), 3.12 (s, 3H, NCH_3), 2.61 (br. s, 6H, $N(CH_3)_2$), 2.01 (s, 3H, CCH_3), 0.99 (s, 9H, $C(CH_3)_3$); **^{13}C {^{1}H} NMR** (100 MHz, $CDCl_3$): δ_C = 172.9 (*C*=O), 162.9 (*C*=O), 142.9 (C_{Ar}), 134.8 (2×C_{Ar}Cl), 128.4 (CH_{Ar}), 125.1 (2×CH_{Ar}), 81.6 ($CHC(CH_3)_3$), 68.6 (CCH_3), 38.4 ($N(CH_3)_2$), 37.4 ($C(CH_3)_3$), 32.1 (NCH_3), 26.3 ($C(CH_3)_3$), 23.1 (CCH_3); **HRMS** (ESI+): *m/z* calcd for $C_{18}H_{25}ClN_3O_2Na$ ($[M+Na]^+$) 408.1216, found 408.1222.

(2*S*,5*S*)-5-Benzyl-2-(*tert*-butyl)-5-(4-chlorophenyl)-*N*,*N*,3-trimethyl-4-oxoimidazolidine-1-carboxamide (3f)

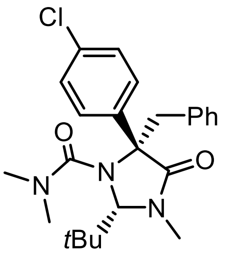

To a solution of *N*-chloroformylimidazolidinone **S9** (700 mg, 2.27 mmol, 1.00 eq.) and 4-chloro-*N*-methylaniline (385 mg, 2.72 mmol, 1.20 eq.) in anhydrous THF (23.0 mL, 0.1 M) at −78 °C was added, dropwise, KHMDS (1.0 M in THF, 2.72 mL, 1.2 eq.). The reaction mixture was allowed to warm to 0 °C over ca. 2 h before additional KHMDS (1.0 M in THF, 2.72 mL, 1.2 eq.) was added dropwise. After 15 min, the mixture was allowed to warm to room temperature and stirred for a further 2 h. MeI (0.42 mL, 6.80 mmol, 3.00 eq.) was added dropwise and the mixture was left to stir for a further 16 h. HCl (1 M, aq.) was added and, after dilution with EtOAc, the layers were separated. The organic layer was washed with HCl (1 M, aq., 2×), $Na_2S_2O_3$ (aq., 2×) and brine. The combined organic layers were dried over $MgSO_4$, filtered and concentrated *in vacuo*. Purification by automated FCC (10%→50% EtOAc in Pet.Ether) gave the title compound (864 mg, 2.02 mmol, 89%) as a white solid. **3f: ^{1}H NMR** (500 MHz, $CDCl_3$): δ_H = 7.40 (d, *J* = 7.3 Hz, 2H, CH_{Ph}), 7.31 – 7.17 (m, 5H, CH_{Ph} and CH_{Ar}), 7.15 (d, *J* = 8.2 Hz, 2H,

CH_{Ar}), 5.37 (s, 1H, C*H*C(CH_3)$_3$), 3.85 (d, *J* = 14.1 Hz, 1H, CH_AH_BPh), 3.76 (d, *J* = 14.4 Hz, 1H, CH_AH_BPh), 3.11 (s, 3H, NCH_3), 2.79 (s, 6H, N(CH_3)$_2$), 0.56 (s, 9H, C(CH_3)$_3$); **^{13}C {^{1}H} NMR** (125 MHz, $CDCl_3$): δ_C = 172.1 (*C*=O), 163.8 (*C*=O), 137.9 (C_{Ar}), 136.0 (C_{Ar}), 134.0 (C_{Ar}), 131.2 (2×CH_{Ar}), 128.5 (4×CH_{Ar}), 128.0 (2×CH_{Ar}), 127.2 (CH_{Ph}), 81.6 (*C*HC(CH_3)$_3$), 74.0 (*C*CH$_2$Ph), 40.1 (*C*H$_2$Ph), 38.5 (N(CH_3)$_2$), 36.3 (*C*(CH_3)$_3$), 31.5 (NCH_3), 25.9 (C(CH_3)$_3$); **HRMS** (ESI$^+$): *m/z* calcd for $C_{24}H_{31}ClN_3O_2$ ([M+H]$^+$) 428.2099, found 428.2096.

(2*S*,5*S*)-5-Benzyl-2-(*tert*-butyl)-*N*,*N*,3-trimethyl-4-oxo-5-(*p*-tolyl)imidazolidine-1-carboxamide (3g)

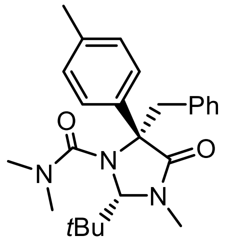

To a solution of *N*-chloroformylimidazolidinone **S9** (600 mg, 1.94 mmol, 1.00 eq.) and *N*-methyl-*p*-toluidine (283 mg, 2.33 mmol, 1.20 eq.) in anhydrous THF (210 mL, 0.1 M) at −78 °C was added, dropwise, KHMDS (1.0 M in THF, 2.33 mL, 1.2 eq.). The reaction mixture was allowed to warm to 0 °C over ca. 2 h before additional KHMDS (1.0 M in THF, 2.33 mL, 1.2 eq.) was added dropwise. After 15 min, the mixture was allowed to warm to room temperature and stirred for a further 2 h. MeI (0.36 mL, 5.83 mmol, 3.00 eq.) was added dropwise and the mixture was left to stir for a further 16 h. HCl (1 M, aq.) was added and, after dilution with EtOAc, the layers were separated. The organic layer was washed with HCl (1 M, aq., 2×), $Na_2S_2O_3$ (aq., 2×) and brine. The combined organic layers were dried over $MgSO_4$, filtered and concentrated *in vacuo* to give the title compound (637 mg, 1.56 mmol, 80%) as a white solid. **3g: ^{1}H NMR** (500 MHz, $CDCl_3$): δ_H = 7.42 (d, *J* = 7.4, 2H, CH_{Ph}), 7.24 (t, *J* = 7.8, 2H, CH_{Ph}), 7.19 (t, *J* = 7.2, 1H, CH_{Ph}), 7.10 (s, 4H, CH_{Ar}), 5.39 (s, 1H, C*H*C(CH_3)$_3$), 3.95 – 3.73 (m, 2H, CH_2Ph), 3.12 (s, 3H, NCH_3), 2.77 (br. s, 6H, N(CH_3)$_2$), 2.30 (s, 3H, CH_3), 0.57 (s, 9H, C(CH_3)$_3$); **^{13}C {^{1}H} NMR** (125 MHz, $CDCl_3$): δ_C = 172.7 (*C*=O), 164.0 (*C*=O), 137.9 (C_{Ar}), 136.6 (C_{Ar}), 136.4 (C_{Ar}), 131.2 (2×CH_{Ph}), 128.6 (2×CH_{Ar}), 128.4 (2×CH_{Ph}), 127.0 (CH_{Ph}), 126.8 (2×CH_{Ar}), 81.6 (*C*HC(CH_3)$_3$), 74.4 (*C*CH$_2$Ph), 40.1 (*C*H$_2$Ph), 38.5 (N(CH_3)$_2$), 36.4 (*C*(CH_3)$_3$), 31.6 (NCH_3), 26.0 (C(CH_3)$_3$), 21.2 (CH_3); **HRMS** (ESI$^+$): *m/z* calcd for $C_{25}H_{34}N_3O_2$ ([M+H]$^+$) 408.2646, found 408.2648.

(2*S*,5*S*)-5-Benzyl-5-(3-bromophenyl)-2-(*tert*-butyl)-*N*,*N*,3-trimethyl-4-oxoimidazolidine-1-carboxamide (3h)

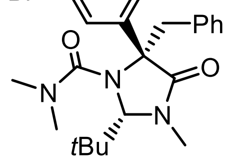

To a solution of *N*-chloroformylimidazolidinone **S9** (500 mg, 1.62 mmol, 1.00 eq.) and 3-bromo-*N*-methylaniline (331 mg, 1.78 mmol, 1.10 eq.) in anhydrous THF (16.0 mL, 0.1 M) at −78 °C was added, dropwise, KHMDS (1.0 M in THF, 1.94 mL, 1.2 eq.). The reaction mixture was allowed to warm to 0 °C over ca. 2 h before additional KHMDS (1.0 M in THF, 1.94 mL, 1.2 eq.) was added dropwise. After 15 min, the mixture was allowed to warm to room temperature and stirred for a further 2 h. MeI (0.30 mL, 4.86 mmol, 3.00 eq.) was added dropwise and the mixture was left to stir for a further 16 h. HCl (1 M, aq.) was added

and, after dilution with EtOAc, the layers were separated. The organic layer was washed with HCl (1 M, aq., 2×), $Na_2S_2O_3$ (aq., 2×) and brine. The combined organic layers were dried over $MgSO_4$, filtered and concentrated *in vacuo* to give the title compound (723 mg, 1.53 mmol, 95%) as a white solid. **3h:** ^{1}**H NMR** (400 MHz, $CDCl_3$): δ_H = 7.45 (t, *J* = 1.9, 1H, C*H*$_{Ar}$), 7.44 – 7.37 (m, 3H, C*H*$_{Ar}$ and 2×C*H*$_{Ph}$), 7.28 – 7.19 (m, 3H, C*H*$_{Ar}$ and 2×C*H*$_{Ph}$), 7.19 (t, *J* = 7.9, 1H, C*H*$_{Ph}$), 7.11 – 7.02 (m, 1H, C*H*$_{Ar}$), 5.36 (s, 1H, C*H*C(CH_3)$_3$), 3.90 – 3.69 (m, 2H, C*H*$_2$Ph), 3.12 (s, 3H, NC*H*$_3$), 2.81 (br. s, 6H, N(C*H*$_3$)$_2$), 0.57 (s, 9H, C(C*H*$_3$)$_3$); ^{13}**C {^{1}H} NMR** (100 MHz, $CDCl_3$): δ_C = 171.7 (*C*=O), 163.6 (*C*=O), 141.4 (*C*$_{Ar}$), 135.9 (*C*$_{Ph}$), 131.2 (3×CH$_{Ar}$), 129.8 (*C*H$_{Ar}$), 129.5 (*C*H$_{Ar}$), 128.4 (2×*C*H$_{Ar}$), 127.2 (*C*H$_{Ar}$), 126.1 (*C*H$_{Ar}$), 121.9 (*C*$_{Ar}$Br), 81.6 (*C*HC(CH_3)$_3$), 74.0 (*C*CH$_2$Ph), 40.0 (*C*H$_2$Ph), 38.4 (N(*C*H$_3$)$_2$), 36.3 (*C*(CH_3)$_3$), 31.4 (N*C*H$_3$), 25.8 (C(*C*H$_3$)$_3$); **HRMS** (ESI$^+$): *m/z* calcd for $C_{24}H_{30}BrN_3O_2Na$ ([M+Na]$^+$) 494.1414, found 494.1409.

(2*S*,5*S*)-5-(4-(Benzyloxy)benzyl)-2-(*tert*-butyl)-*N*,*N*,3-trimethyl-4-oxo-5-(*p*-tolyl)imidazolidine-1-carboxamide (3i)

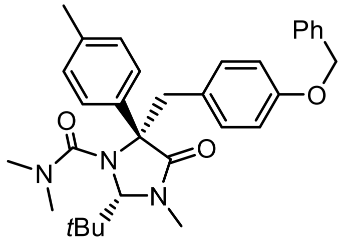

To a solution of *N*-chloroformylimidazolidinone **S10** (700 mg, 1.69 mmol, 1.00 eq.) and *N*-methyl-*p*-toluidine (245 mg, 2.02 mmol, 1.20 eq.) in anhydrous THF (17.0 mL, 0.1 M) at −78 °C was added, dropwise, KHMDS (1.0 M in THF, 2.00 mL, 1.2 eq.). The reaction mixture was allowed to warm to 0 °C over ca. 2 h before additional KHMDS (1.0 M in THF, 2.00 mL, 1.2 eq.) was added dropwise. After 15 min, the mixture was allowed to warm to room temperature and stirred for a further 2 h. MeI (0.32 mL, 5.06 mmol, 3.00 eq.) was added dropwise and the mixture was left to stir for a further 16 h. HCl (1 M, aq.) was added and, after dilution with EtOAc, the layers were separated. The organic layer was washed with HCl (1 M, aq., 2×), $Na_2S_2O_3$ (aq., 2×) and brine. The combined organic layers were dried over $MgSO_4$, filtered and concentrated *in vacuo* to give the title compound (760 mg, 1.48 mmol, 88%) as a white solid. **3i:** ^{1}**H NMR** (400 MHz, $CDCl_3$): δ_H = 7.43 – 7.28 (m, 7H, C*H*$_{Ar}$), 7.14 – 7.05 (m, 4H, C*H*$_{Ar}$), 6.90 – 6.82 (m, 2H, C*H*$_{Ar}$), 5.39 (s, 1H, C*H*C(CH_3)$_3$), 5.04 (s, 2H, OC*H*$_2$Ph), 3.93 – 3.64 (m, 2H, NCC*H*$_2$), 3.11 (s, 3H, NC*H*$_3$), 2.76 (s, 6H, N(C*H*$_3$)$_2$), 2.30 (s, 3H, C*H*$_3$), 0.60 (s, 9H, C(C*H*$_3$)$_3$); ^{13}**C {^{1}H} NMR** (100 MHz, $CDCl_3$): δ_C = 172.7 (*C*=O), 164.1 (*C*=O), 158.0 (*C*$_{Ar}$O), 137.8 (*C*$_{Ar}$), 137.3 (*C*$_{Ar}$), 136.4 (*C*$_{Ar}$), 132.2 (2×*C*H$_{Ar}$), 129.0 (*C*$_{Ar}$), 128.6 (4×*C*H$_{Ar}$), 127.9 (*C*H$_{Ar}$), 127.5 (2×*C*H$_{Ar}$), 126.9, (2×*C*H$_{Ar}$) 114.9 (2×*C*H$_{Ar}$), 81.6 (*C*HC(CH_3)$_3$), 74.5 (N*C*CH$_2$), 70.0 (O*C*H$_2$Ph), 39.3 (NC*C*H$_2$), 38.5 (N(*C*H$_3$)$_2$), 36.4 (*C*(CH_3)$_3$), 31.5 (N*C*H$_3$), 26.0 (C(*C*H$_3$)$_3$), 21.2 (*C*H$_3$); **HRMS** (ESI$^+$): *m/z* calcd for $C_{32}H_{40}N_3O_3$ ([M+H]$^+$) 514.3075, found 514.3064.

(2*S*,5*S*)-5-(4-(Benzyloxy)benzyl)-5-(4-bromo-3-fluorophenyl)-2-(*tert*-butyl)-*N*,*N*,3-trimethyl-4-oxoimidazolidine-1-carboxamide (3j)

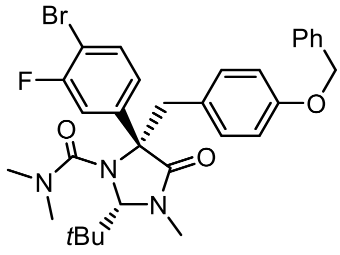

To a solution of *N*-chloroformylimidazolidinone **S10** (500 mg, 1.21 mmol, 1.00 eq.) and 4-bromo-3-fluoro-*N*-methylaniline (295 mg, 1.45 mmol, 1.20 eq.) in anhydrous THF (12.0 mL, 0.1 M) at −78 °C was added, dropwise, KHMDS (1.0 M in THF, 1.45 mL, 1.2 eq.). The reaction mixture was allowed to warm to 0 °C over ca. 2 h before additional KHMDS (1.0 M in THF, 1.45 mL, 1.2 eq.) was added dropwise. After 15 min, the mixture was allowed to warm to room temperature and stirred for a further 2 h. MeI (0.23 mL, 3.62 mmol, 3.00 eq.) was added dropwise and the mixture was left to stir for a further 16 h. HCl (1 M, aq.) was added and, after dilution with EtOAc, the layers were separated. The organic layer was washed with HCl (1 M, aq., 2×), $Na_2S_2O_3$ (aq., 2×) and brine. The combined organic layers were dried over $MgSO_4$, filtered and concentrated *in vacuo*. The crude product was purified by automated FCC to give the title compound (668 mg, 1.12 mmol, 93%) as a white solid. **3j: ^{1}H NMR** (400 MHz, $CDCl_3$): δ_H = 7.48 (dd, *J* = 8.5, 7.2, 1H, CH_{Ar}), 7.44 – 7.25 (m, 7H, CH_{Ar}), 7.03 (d, *J* = 10.2, 1H, CH_{Ar}), 6.91 – 6.80 (m, 2H, CH_{Ar}), 5.36 (s, 1H, C*H*C(CH_3)$_3$), 5.04 (s, 2H, OCH_2Ph), 3.85 – 3.59 (m, 2H, NCCH_2), 3.11 (s, 3H, NCH_3), 2.81 (s, 6H, N(CH_3)$_2$), 0.58 (s, 9H , C(CH_3)$_3$); **^{13}C {^{1}H} NMR** (125 MHz, $CDCl_3$): δ_C = 171.6 (*C*=O), 163.7 (*C*=O), 158.6 (d, $^1J_{C-F}$ = 247.2, C_{Ar}F), 158.1 (C_{Ar}O), 141.2 (C_{Ar}), 137.1 (C_{Ar}), 132.8 (C_{Ar}), 132.2 (2×CH_{Ar}), 128.6 (2×CH_{Ar}), 128.0 (CH_{Ar}), 127.9 (C_{Ar}), 127.5 (2×CH_{Ar}), 124.2 (CH_{Ar}), 115.6 (d, $^2J_{C-F}$ = 23.6, CH_{Ar}), 115.0 (2×CH_{Ar}), 108.8 (d, $^2J_{C-F}$ = 20.9, C_{Ar}Br), 81.7 (*C*HC(CH_3)$_3$), 73.8 (N*C*CH$_2$), 70.0 (O*C*H$_2$Ph), 39.2 (NC*C*H$_2$), 38.6 (N(*C*H$_3$)$_2$), 36.4 (*C*(CH_3)$_3$), 31.6 (N*C*H$_3$), 25.9 (C(*C*H$_3$)$_3$); **HRMS** (ESI$^+$): *m/z* calcd for $C_{31}H_{35}BrFN_3O_3Na$ ([M+Na]$^+$) 618.1738, found 618.1743.

(*R*)-2-((((9*H*-Fluoren-9-yl)methoxy)carbonyl)amino)-2-(4-bromophenyl)propanoic acid (*R*-4a)

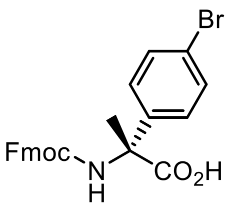

Urea **7a** (1.80 g, 4.54 mmol, 1.00 eq.) was split into two batches, each was suspended in HCl (6.0 M, aq.)/EtOH (10:1, 0.5 M, 4.50 mL) and heated in a microwave reactor at 160 °C for 3 h. The cooled reaction mixtures were combined, diluted with H_2O and washed with DCM (3×) before being concentrated *in vacuo*. The residue was redissolved in MeOH/H_2O (1:1) and the solution neutralised by addition of Na_2CO_3 (3.50 eq.). The mixture was again concentrated *in vacuo*, resuspended in MeOH and filtered to remove most of the salts. Concentration *in vacuo* gave the crude amino acid which was used without further purification. The crude amino acid was dissolved in H_2O/dioxane (30.0 mL, 1:1) and Na_2CO_3 was added until pH 8-9. Fmoc-OSu (2.29 g, 6.79 mmol, 1.50 eq.) was added, and the resulting suspension was stirred for 16 h at room temperature. H_2O (15 mL) was added before the

mixture was acidified with conc. HCl and extracted with EtOAc (2×). The combined organic layers were dried over $MgSO_4$ and concentrated *in vacuo*. Purification by automated FCC (0.5%→3.5% MeOH in DCM, 0.25% AcOH) gave the title compound (1.51 g, 3.24 mmol, 71%) as a white solid. ***R*-4a:** $[\alpha]_D^{22}$ = −32 (*c* = 0.25 in $CHCl_3$); **R_f** = 0.18 (2% MeOH in CH_2Cl_2, 0.25% AcOH); **^{1}H NMR** (400 MHz, $(CD_3)_2SO$): δ_H = 12.91 (br. s, 1H, COO*H*), 7.89 (d, *J* = 7.5, 2H, CH_{Ar}), 7.86 – 7.71 (m, 2H, CH_{Ar}), 7.56 – 7.50 (m, 2H, CH_{Ar}), 7.45 – 7.37 (m, 4H, CH_{Ar}), 7.37 – 7.29 (m, 2H, CH_{Ar}), 4.32 – 4.15 (m, 3H, Fmoc-CH_2, Fmoc-C*H*), 1.74 (s, 3H, CH_3); **^{13}C {^{1}H} NMR** (100 MHz, $(CD_3)_2SO$): δ_C = 173.3 (*C*OOH), 154.8 (*C*=O), 143.8 (2×Fmoc-C_{Ar}), 140.7 (2×Fmoc-C_{Ar}, C_{Ar}), 130.8 (2×CH_{Ar}), 128.7 (2×CH_{Ar}), 127.6 (2×CH_{Ar}), 127.1 (2×CH_{Ar}), 125.3 (2×CH_{Ar}), 120.5 (C_{Ar}), 120.1 (2×CH_{Ar}), 65.5 (Fmoc-CH_2), 61.0 (*C*CH_3), 46.7 (Fmoc-*C*H), 24.2 (CH_3); **HRMS** (ESI$^+$): *m/z* calcd for $C_{24}H_{20}BrNO_4$ ([M+H]$^+$) 466.0648, found 466.0646.

(*S*)-2-(((9*H*-Fluoren-9-yl)methoxy)carbonyl)amino)-2-(4-bromophenyl)propanoic acid (*S*-4a)

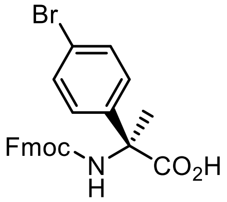

Urea **3a** (1.80 g, 4.54 mmol, 1.00 eq.) was split into two batches, each was suspended in HCl (6.0 M, aq.)/EtOH (10:1, 0.5 M, 4.50 mL) and heated in a microwave reactor at 160 °C for 3 h. The cooled reaction mixtures were combined, diluted with H_2O and washed with DCM (3×) before being concentrated *in vacuo*. The residue was redissolved in MeOH/H_2O (1:1) and the solution neutralised by addition of Na_2CO_3 (3.50 eq.). The mixture was again concentrated *in vacuo*, resuspended in MeOH and filtered to remove most of the salts. Concentration *in vacuo* gave the crude amino acid as a white solid which was used without further purification. The crude amino acid was dissolved in H_2O/dioxane (30.0 mL, 1:1) and Na_2CO_3 was added until pH 8-9. Fmoc-OSu (2.29 g, 6.79 mmol, 1.50 eq.) was added, and the resulting suspension was stirred for 16 h at room temperature. H_2O (15.0 mL) was added before the mixture was acidified with conc. HCl and extracted with EtOAc (2×). The combined organic layers were dried over $MgSO_4$ and concentrated *in vacuo*. Purification by automated FCC (0.5%→3.5% MeOH in DCM, 0.25% AcOH) gave the title compound (1.76 g, 3.77 mmol, 83%) as a white solid. ***S*-4a:** $[\alpha]_D^{22}$ = +32 (*c* = 0.25 in $CHCl_3$); **HRMS** (ESI$^+$): *m/z* calcd for $C_{24}H_{20}BrNO_4$ ([M+H]$^+$) 466.0648, found 466.0644. For full data see compound ***R*-4a**.

(*S*)-2-(((9*H*-Fluoren-9-yl)methoxy)carbonyl)amino)-2-(3-bromophenyl)propanoic acid (4b)

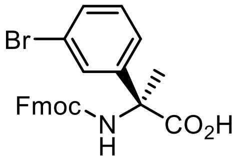

Urea **3b** (2.50 g, 6.31 mmol, 1.00 eq.) was split into two batches, each was suspended in HCl (6.0 M, aq.)/EtOH (9:1, 0.5 M, 6.30 mL) and heated in a microwave reactor at 160 °C for 3 h. The cooled reaction mixtures were combined, diluted with H_2O and washed with DCM (3×) before being concentrated *in vacuo*. The residue was redissolved in MeOH/H_2O (1:1) and the solution neutralised by addition of Na_2CO_3 (3.50 eq.). The mixture was again concentrated *in vacuo*, resuspended in MeOH and filtered to remove most

of the salts. Concentration *in vacuo* gave the crude amino acid which was used without further purification. The crude amino acid was dissolved in H_2O/dioxane (20.0 mL, 1:1) and Na_2CO_3 was added until pH 8-9. Fmoc-Cl (2.45 g, 9.46 mmol, 1.50 eq.) was added, and the resulting suspension was stirred for 16 h at room temperature. H_2O was added before the mixture was acidified with conc. HCl and extracted with EtOAc (2×). The combined organic layers were dried over $MgSO_4$ and concentrated *in vacuo*. Purification by automated RP FCC (50→100% MeCN in H_2O, 0.1% FA) and freeze drying gave the title compound (1.48 g, 3.17 mmol, 50%) as a white solid. **4b: ^{1}H NMR** (500 MHz, $CDCl_3$, 0.75:0.25 mixture of rotamers): δ_H = 8.24 (s, 0.75H, N$H_{(maj)}$), 7.85 – 7.64 (m, 2H, CH_{Ar}), 7.63 – 6.93 (m, 10H, CH_{Ar}), 6.11 (s, 0.25H, N$H_{(min)}$), 4.57 – 4.29 (m, 2H, Fmoc-CH_2), 4.20 (s, 0.25H, Fmoc-C$H_{(min)}$), 3.90 (s, 0.75H, Fmoc-C$H_{(maj)}$), 1.99 (s, 0.75H, C$H_{3(min)}$), 1.67 (s, 2.25H, C$H_{3(maj)}$); **^{13}C {^{1}H} NMR** (125 MHz, $CDCl_3$, major rotamer): δ_C = 175.4 (*C*OOH), 157.4 (*C*=O), 143.5 (*C*$_{Ar}$), 143.2 (*C*$_{Ar}$), 142.8 (*C*$_{Ar}$), 141.4 (*C*$_{Ar}$), 141.2 (*C*$_{Ar}$), 131.0 (*C*H$_{Ar}$), 129.9 (*C*H$_{Ar}$), 129.4 (*C*H$_{Ar}$), 127.8 (2×*C*H$_{Ar}$), 127.2 (2×*C*H$_{Ar}$), 124.8 (*C*H$_{Ar}$), 124.6 (*C*H$_{Ar}$), 124.2 (*C*H$_{Ar}$), 122.7 (*C*$_{Ar}$), 120.1 (2×*C*H$_{Ar}$), 67.4 (Fmoc-*C*H$_2$), 61.6 (*C*CH$_3$), 47.0 (Fmoc-*C*H), 22.3 (*C*H$_3$); **HRMS** (ESI$^-$): *m/z* calcd for $C_{24}H_{19}BrNO_4$ ([M−H]$^-$) 464.0497, found 464.0495.

(*S*)-2-((((9*H*-Fluoren-9-yl)methoxy)carbonyl)amino)-2-(3,5-difluorophenyl)propanoic acid (4d)

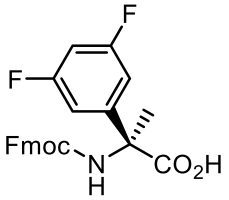

Urea **3d** (620 mg, 1.75 mmol, 1.00 eq.) was suspended in HCl (6.0 M, aq.)/EtOH (9:1, 0.5 M, 3.50 mL) and heated in a microwave reactor at 160 °C for 3 h. The cooled reaction mixture was diluted with H_2O and washed with DCM (3×) before being concentrated *in vacuo*. The residue was redissolved in MeOH/H_2O (1:1) and the solution neutralised by addition of Na_2CO_3 (3.50 eq.). The mixture was again concentrated *in vacuo*, resuspended in MeOH and filtered to remove most of the salts. Concentration *in vacuo* gave the crude amino acid which was used without further purification. The crude amino acid was dissolved in H_2O/dioxane (12.0 mL, 1:1) and Na_2CO_3 was added until pH 8-9. Fmoc-Cl (681 mg, 2.63 mmol, 1.50 eq.) was added, and the resulting suspension was stirred for 16 h at room temperature. H_2O was added before the mixture was acidified with conc. HCl and extracted with EtOAc (2×). The combined organic layers were dried over $MgSO_4$ and concentrated *in vacuo*. Purification by automated RP FCC (50→100% MeCN in H_2O, 0.1% FA) and freeze drying overnight gave the title compound (498 mg, 1.18 mmol, 67%) as a white solid. **4d: ^{1}H NMR** (500 MHz, $CDCl_3$, 0.83:0.17 mixture of rotamers): δ_H = 8.16 (s, 0.83H, N$H_{(maj)}$), 7.84 – 7.61 (m, 2H, CH_{Ar}), 7.60 – 7.02 (m, 6H, CH_{Ar}), 7.03 – 6.44 (m, 3H, CH_{Ar}), 6.12 (s, 0.17H, N$H_{(min)}$), 4.51 (ddd, *J* = 80.5, 10.9, 4.3 Hz, 2H, Fmoc-CH_2), 4.20 (s, 0.17H, Fmoc-C$H_{(min)}$), 3.92 (t, *J* = 4.4 Hz, 0.83H, Fmoc-C$H_{(maj)}$), 1.97 (s, 0.51H, C$H_{3(min)}$), 1.42 (s, 2.49H, C$H_{3(maj)}$); **^{13}C {^{1}H} NMR** (125 MHz, $CDCl_3$, major rotamer): δ_C = 174.8 (*C*OOH), 162.7 (dd, $^1J_{C-F}$ = 248.1, 12.8, 2×*C*$_{Ar}$F), 157.3 (*C*=O), 144.9 – 143.9 (m, *C*$_{Ar}$), 143.2 (*C*$_{Ar}$), 143.1(*C*$_{Ar}$), 141.6 (*C*$_{Ar}$), 141.2 (*C*$_{Ar}$), 127.8 (2×*C*H$_{Ar}$), 127.2 (*C*H$_{Ar}$), 127.1 (*C*H$_{Ar}$), 124.3 (*C*H$_{Ar}$), 123.8 (*C*H$_{Ar}$), 120.2 (*C*H$_{Ar}$), 120.1 (*C*H$_{Ar}$), 110.5 – 108.4 (m, 2×C*H*$_{Ar}$), 103.4 (t, $^2J_{C-F}$ =

25.4, CH_{Ar}), 67.0 (Fmoc-CH_2), 61.4 (CCH_3), 46.9 (Fmoc-CH), 22.0 (CH_3); **HRMS** (ESI^-): *m/z* calcd for $C_{24}H_{18}NO_4F_2$ ($[M-H]^-$) 422.1204, found 422.1205.

(*S*)-2-((((9*H*-Fluoren-9-yl)methoxy)carbonyl)amino)-2-(4-chlorophenyl)-3-phenylpropanoic acid (4f)

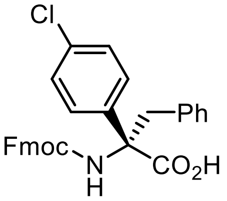

Urea **3f** (300 mg, 0.701 mmol, 1.00 eq.) was suspended in HCl (6.0 M, aq.)/EtOH (9:1, 0.5 M, 1.40 mL) and heated in a microwave reactor at 160 °C for 3 h. The cooled reaction mixture was diluted with H_2O and washed with DCM (3×) before being concentrated *in vacuo*. The residue was redissolved in MeOH/H_2O (1:1) and the solution neutralised by addition of Na_2CO_3 (3.50 eq.). The mixture was again concentrated *in vacuo*, resuspended in MeOH and filtered to remove most of the salts. Concentration *in vacuo* gave the crude amino acid which was used without further purification. The crude amino acid was dissolved in H_2O/dioxane (4.50 mL, 1:1) and Na_2CO_3 was added until pH 8-9. Fmoc-Cl (272 mg, 1.05 mmol, 1.50 eq.) was added, and the resulting suspension was stirred for 16 h at room temperature. H_2O was added before the mixture was acidified with conc. HCl and extracted with EtOAc (2×). The combined organic layers were dried over $MgSO_4$ and concentrated *in vacuo*. Purification by automated RP FCC (50→100% MeCN in H_2O, 0.1% FA) and freeze drying overnight gave the title compound (178 mg, 0.357 mmol, 51%) as a white solid. **4f:** ^{1}**H NMR** (100 MHz, $CDCl_3$, ca. 0.65:0.35 mixture of rotamers): δ_H = 9.00 – 6.53 (m, 17H, CH_{Ar}), 6.50 (s, 0.35H, N$H_{(min)}$), 6.06 (s, 0.65H, N$H_{(maj)}$), 4.90 – 4.37 (m, 0.70H, Fmoc-C$H_{2(min)}$), 4.43 (ddd, *J* = 96.0, 10.8, 6.6 Hz, 1.30H, Fmoc-C$H_{2(maj)}$), 4.20 (t, *J* = 6.6 Hz, 0.65H, Fmoc-C$H_{2(maj)}$), 3.98 (s, 0.35H, Fmoc-C$H_{(min)}$), 3.95 – 3.74 (m, 1.30H, CH_2Ph$_{(maj)}$), 3.42 – 2.66 (m, 0.70H, CH_2Ph$_{(min)}$); ^{13}**C {^{1}H} NMR** (125 MHz, $CDCl_3$, major rotamer): δ_C = 175.5 (*C*OOH), 154.4 (*C*=O), 143.9 (C_{Ar}), 143.6 (C_{Ar}), 141.5 (2×C_{Ar}), 138.1 (C_{Ar}), 135.3 (C_{Ar}), 134.3 (C_{Ar}), 130.1 (2×CH_{Ar}), 129.0 (2×CH_{Ar}), 128.6 (2×CH_{Ar}), 127.9 (2×CH_{Ar}), 127.6 (2×CH_{Ar}), 127.4 (CH_{Ar}), 127.3 (2×CH_{Ar}), 125.2 (CH_{Ar}), 125.0 (CH_{Ar}), 120.1 (2×CH_{Ar}), 66.7 (Fmoc-CH_2), 65.7 (CCH_2Ph), 47.4 (Fmoc-*C*H), 39.0 (CH_2Ph). **HRMS** (ESI^+): *m/z* calcd for $C_{30}H_{25}ClNO_4$ ($[M+H]^+$) 498.1467, found 498.1465.

(*S*)-2-((((9*H*-Fluoren-9-yl)methoxy)carbonyl)amino)-3-phenyl-2-(*p*-tolyl)propanoic acid (4g)

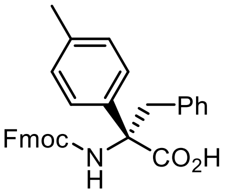

Urea **3g** (580 mg, 1.42 mmol, 1.00 eq.) was suspended in HCl (6.0 M, aq.)/EtOH (9:1, 0.5 M, 3.0 mL) and heated in a microwave reactor at 160 °C for 3 h. The cooled reaction mixture was diluted with H_2O and washed with DCM (3×) before being concentrated *in vacuo*. The residue was redissolved in MeOH/H_2O (1:1) and the solution neutralised by addition of Na_2CO_3 (3.50 eq.). The mixture was again concentrated *in vacuo*, resuspended in MeOH and filtered to remove most of the salts. Concentration *in vacuo* gave the crude amino acid which was used without further purification. The crude amino acid was dissolved in H_2O/dioxane (9 mL, 1:1) and Na_2CO_3 was added until pH 8-9. Fmoc-Cl (554 mg, 2.13 mmol, 1.50 eq.)

was added, and the resulting suspension was stirred for 16 h at room temperature. H_2O was added before the mixture was acidified with conc. HCl and extracted with EtOAc (2×). The combined organic layers were dried over $MgSO_4$ and concentrated *in vacuo*. Purification by automated RP FCC (50→100% MeCN in H_2O, 0.1% FA) and freeze drying overnight gave the title compound (421 mg, 0.88 mmol, 62%) as a white solid. **4g: ^{1}H NMR** (500 MHz, $CDCl_3$): δ_H = 7.78 (d, *J* = 7.7 Hz, 2H, CH_{Ar}), 7.54 (dd, *J* = 13.4, 7.5 Hz, 2H, CH_{Ar}), 7.41 (t, *J* = 7.4 Hz, 2H, CH_{Ar}), 7.37 – 7.13 (m, 9H, CH_{Ar}), 7.06 (d, *J* = 7.4 Hz, 2H, CH_{Ar}), 6.04 (s, 1H, N*H*), 4.58 – 4.24 (m, 2H, Fmoc-CH_2), 4.21 (t, *J* = 7.0 Hz, 1H, Fmoc-C*H*), 3.94 – 3.78 (m, 2H, CH_2Ph), 2.35 (s, 3H, CH_3); **^{13}C {^{1}H} NMR** (125 MHz, $CDCl_3$): δ_C = 175.5 (*C*OOH), 154.6 (*C*=O), 144.0 (C_{Ar}), 143.8 (C_{Ar}), 141.50 (C_{Ar}), 141.46 (C_{Ar}), 138.2 (C_{Ar}), 136.4 (C_{Ar}), 135.7 (C_{Ar}), 130.2 (2×CH_{Ar}), 129.6 (2×CH_{Ar}), 128.6 (2×CH_{Ar}), 127.9 (2×CH_{Ar}), 127.3 (2×CH_{Ar}), 127.2 (CH_{Ar}), 125.9 (2×CH_{Ar}), 125.3 (CH_{Ar}), 125.1 (CH_{Ar}), 120.14 (CH_{Ar}), 120.12 (CH_{Ar}), 66.7 (Fmoc-CH_2), 65.9 (CCH$_2$Ph), 47.4 (Fmoc-*C*H), 39.4 (CH$_2$Ph), 21.2 (CH_3); **HRMS** (ESI$^+$): *m/z* calcd for $C_{31}H_{28}NO_4$ ([M+H]$^+$) 478.2013, found 478.2009.

(*S*)-2-((((9*H*-Fluoren-9-yl)methoxy)carbonyl)amino)-2-(3-bromophenyl)-3-phenylpropanoic acid (4h)

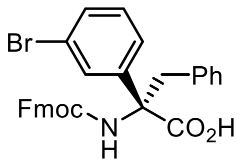

Urea **3h** (680 mg, 1.44 mmol, 1.00 eq.) was suspended in HCl (6.0 M, aq.)/EtOH (9:1, 0.5 M, 3.00 mL) and heated in a microwave reactor at 160 °C for 3 h. The cooled reaction mixture was diluted with H_2O and washed with DCM (3×) before being concentrated *in vacuo*. The residue was redissolved in MeOH/H_2O (1:1) and the solution neutralised by addition of Na_2CO_3 (3.50 eq.). The mixture was again concentrated *in vacuo*, resuspended in MeOH and filtered to remove most of the salts. Concentration *in vacuo* gave the crude amino acid which was used without further purification. The crude amino acid was dissolved in H_2O/dioxane (9.0 mL, 1:1) and Na_2CO_3 was added until pH 8-9. Fmoc-Cl (559 mg, 2.16 mmol, 1.50 eq.) was added, and the resulting suspension was stirred for 16 h at room temperature. H_2O was added before the mixture was acidified with conc. HCl and extracted with EtOAc (2×). The combined organic layers were dried over $MgSO_4$ and concentrated *in vacuo*. Purification by automated RP FCC (50→100% MeCN in H_2O, 0.1% FA) and freeze drying overnight gave the title compound (389 mg, 0.717 mmol, 50%) as a white solid. **4h: ^{1}H NMR** (400 MHz, $CDCl_3$, major rotamer): δ_H = 7.78 (d, *J* = 7.6 Hz, 2H, CH_{Ar}), 7.64 (s, 1H, CH_{Ar}), 7.59 – 7.10 (m, 12H, CH_{Ar}) 7.04 (d, *J* = 7.5 Hz, 2H, CH_{Ar}), 6.04 (s, 1H, N*H*), 4.59 – 4.25 (m, 2H, Fmoc-CH_2), 4.25 – 4.16 (m, 1H, Fmoc-C*H*), 3.95 – 3.73 (m, 2H, CH_2Ph); **^{13}C {^{1}H} NMR** (125 MHz, $CDCl_3$): δ_C = 174.7 (*C*OOH), 154.4 (*C*=O), 143.9 (C_{Ar}), 143.7 (C_{Ar}), 141.8 (C_{Ar}), 141.5 (2×C_{Ar}), 135.2 (C_{Ar}), 131.6 (CH_{Ar}), 130.3 (CH_{Ar}), 130.1 (2×CH_{Ar}), 129.5 (CH_{Ar}), 128.7 (2×CH_{Ar}), 127.9 (2×CH_{Ar}), 127.5 (CH_{Ar}), 127.3 (2×CH_{Ar}), 125.3 (CH_{Ar}), 125.1 (CH_{Ar}), 124.8 (CH_{Ar}), 123.1 (C_{Ar}), 120.2 (2×CH_{Ar}), 66.9 (Fmoc-CH_2), 65.7 (CCH$_2$Ph), 47.4 (Fmoc-*C*H), 39.1 (CH$_2$Ph); **HRMS** (ESI$^+$): *m/z* calcd for $C_{30}H_{25}BrNO_4$ ([M+H]$^+$) 542.0961, found 542.0965.

^{1}H and ^{13}C NMR Spectra

(2*R*,5*S*)-*N*-(4-Bromophenyl)-2-(*tert*-butyl)-*N*,3,5-trimethyl-4-oxoimidazolidine-1-carboxamide (6a)

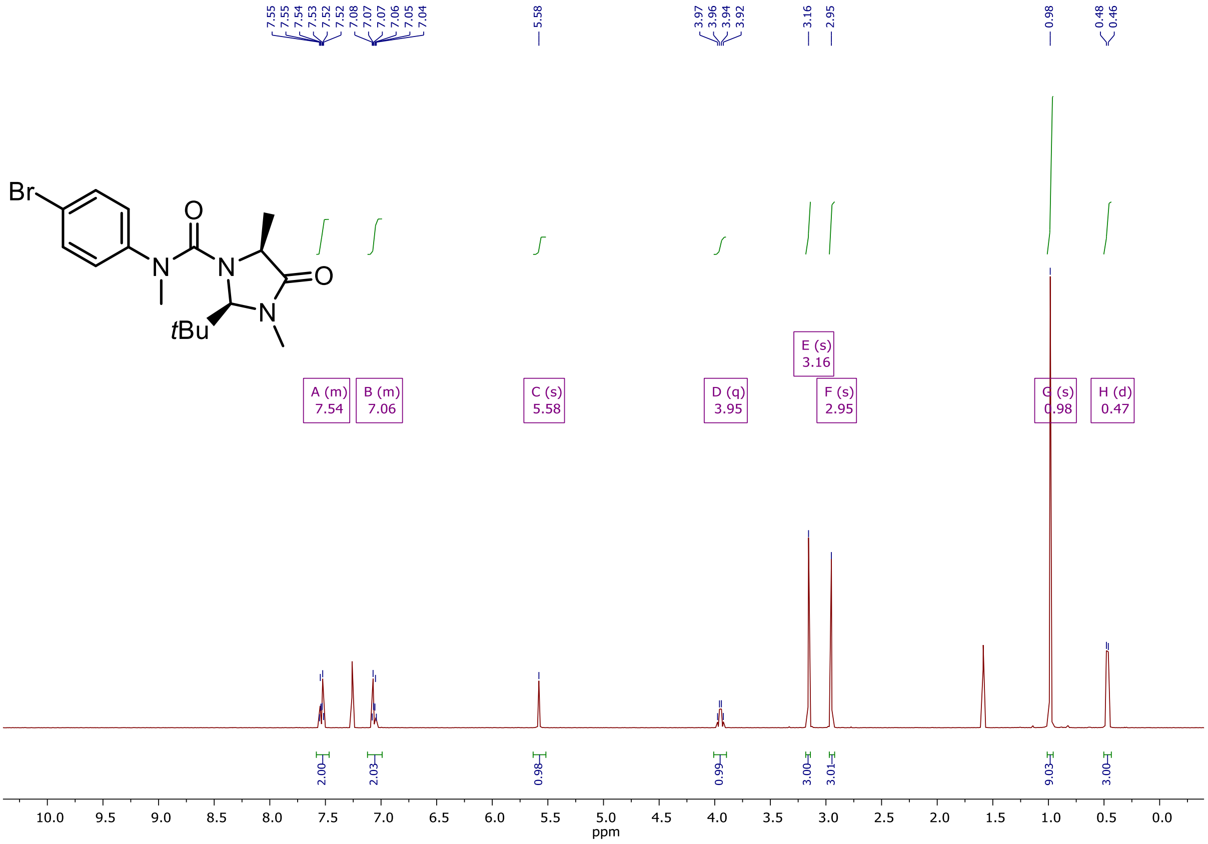

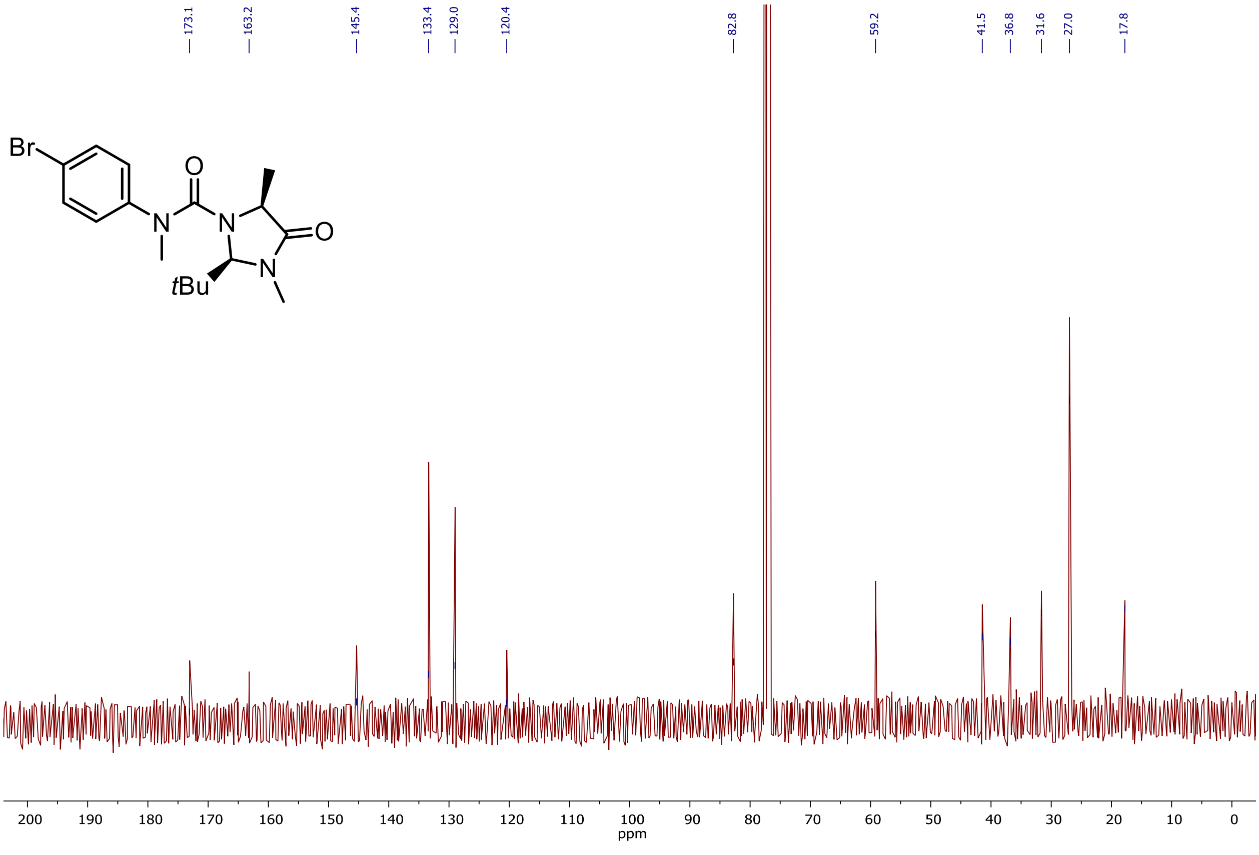

(2*R*,5*R*)-5-(4-Bromophenyl)-2-(*tert*-butyl)-*N*,3,5-trimethyl-4-oxoimidazolidine-1-carboxamide (7a)

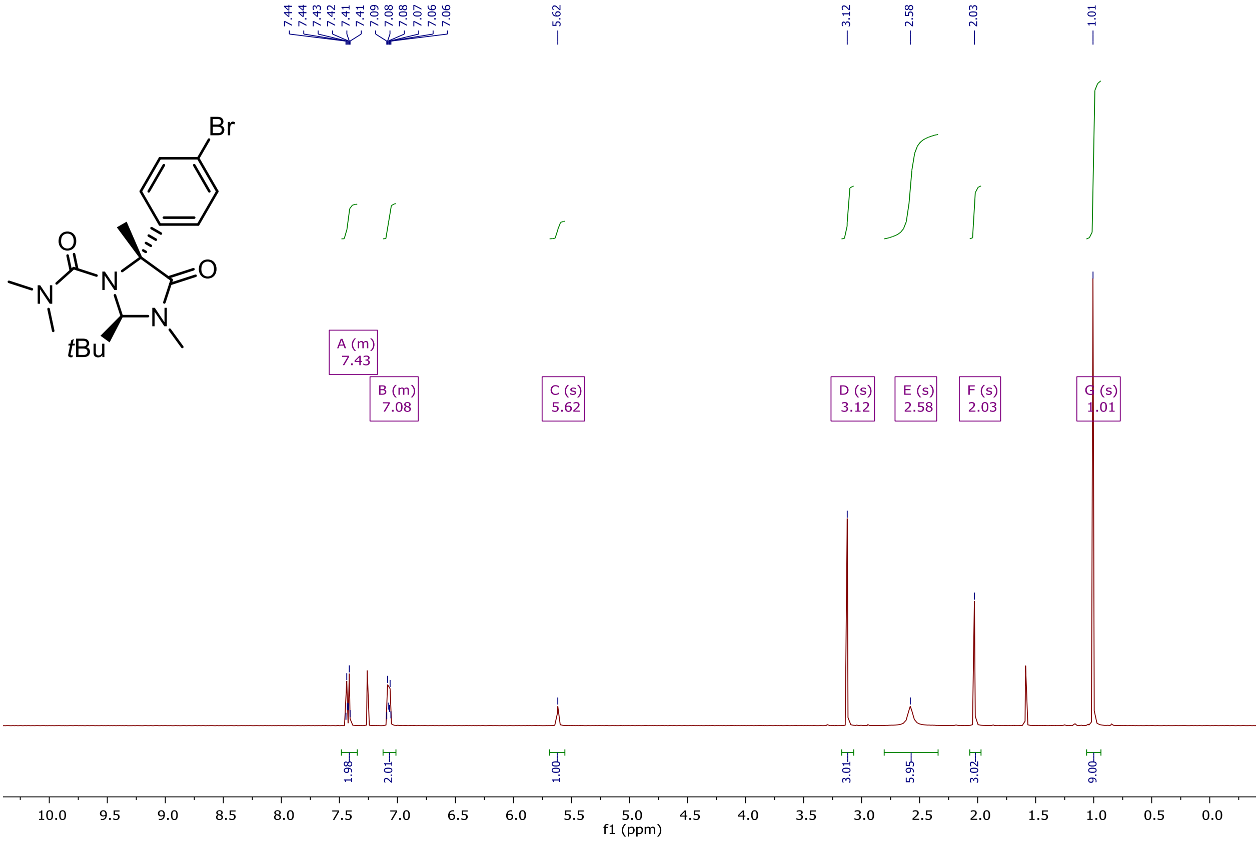

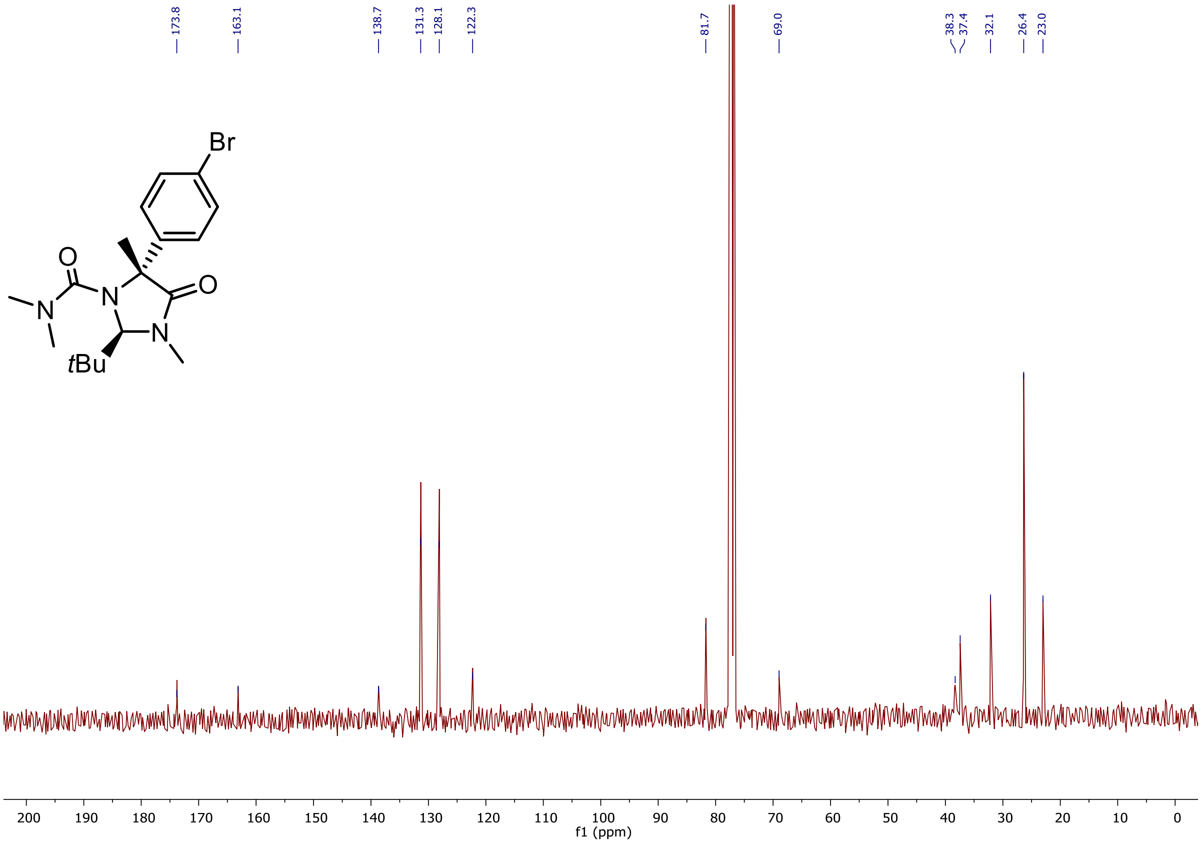

(2*S*,5*S*)-5-(3-Bromophenyl)-2-(*tert*-butyl*)*-*N*,*N*,*3*,*5*-trimethyl-4-oxoimidazolidine-1-carboxamide (3b)

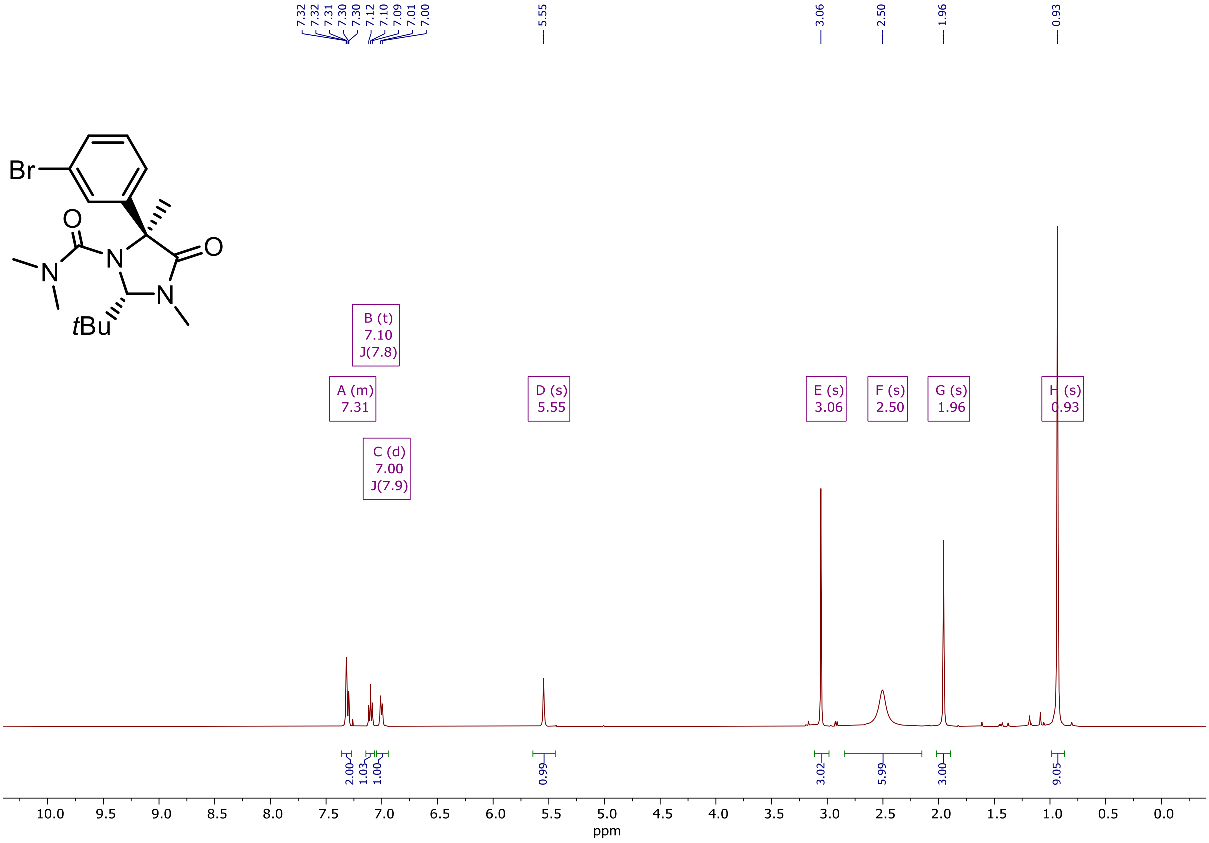

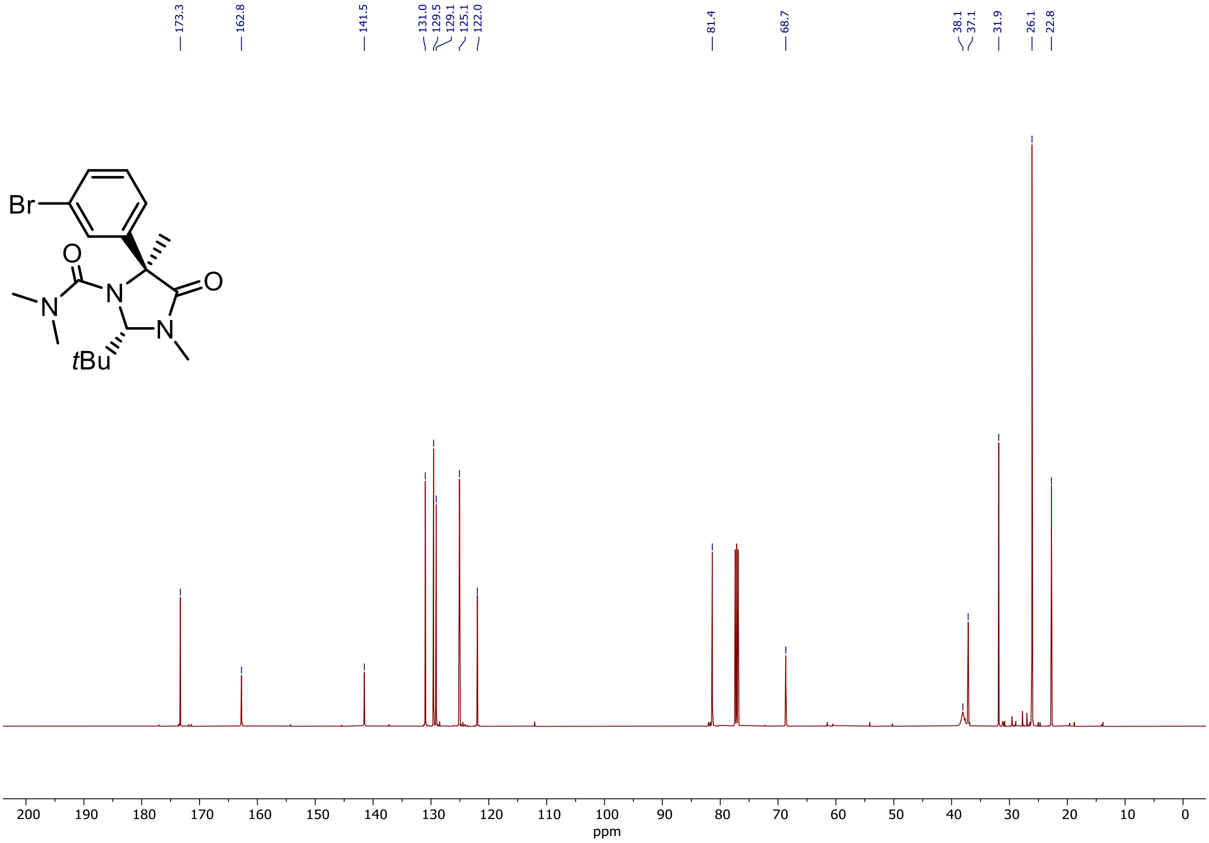

(2*S*,5*S*)-2-(*tert*-Butyl)-5-(3-cyanophenyl)-*N*,*N*,3,5-trimethyl-4-oxoimidazolidine-1-carboxamide (3c)

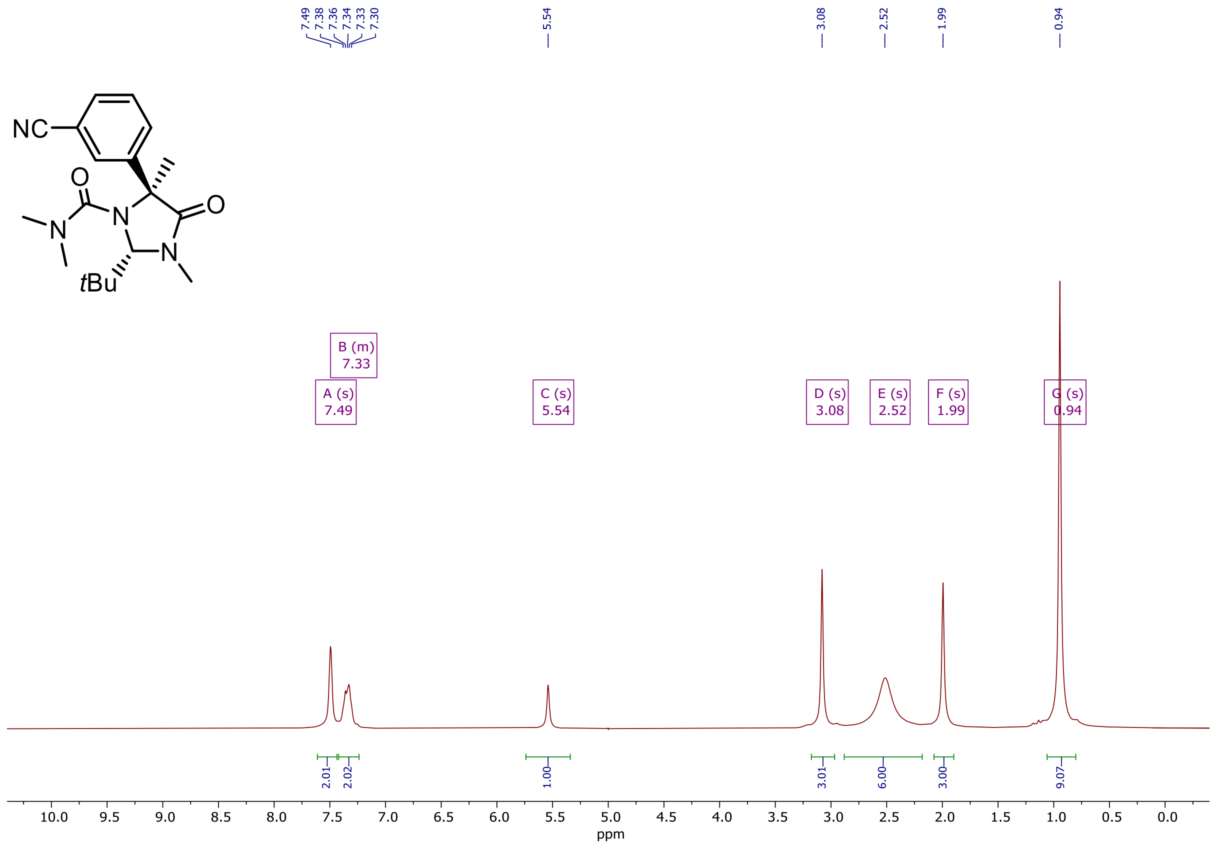

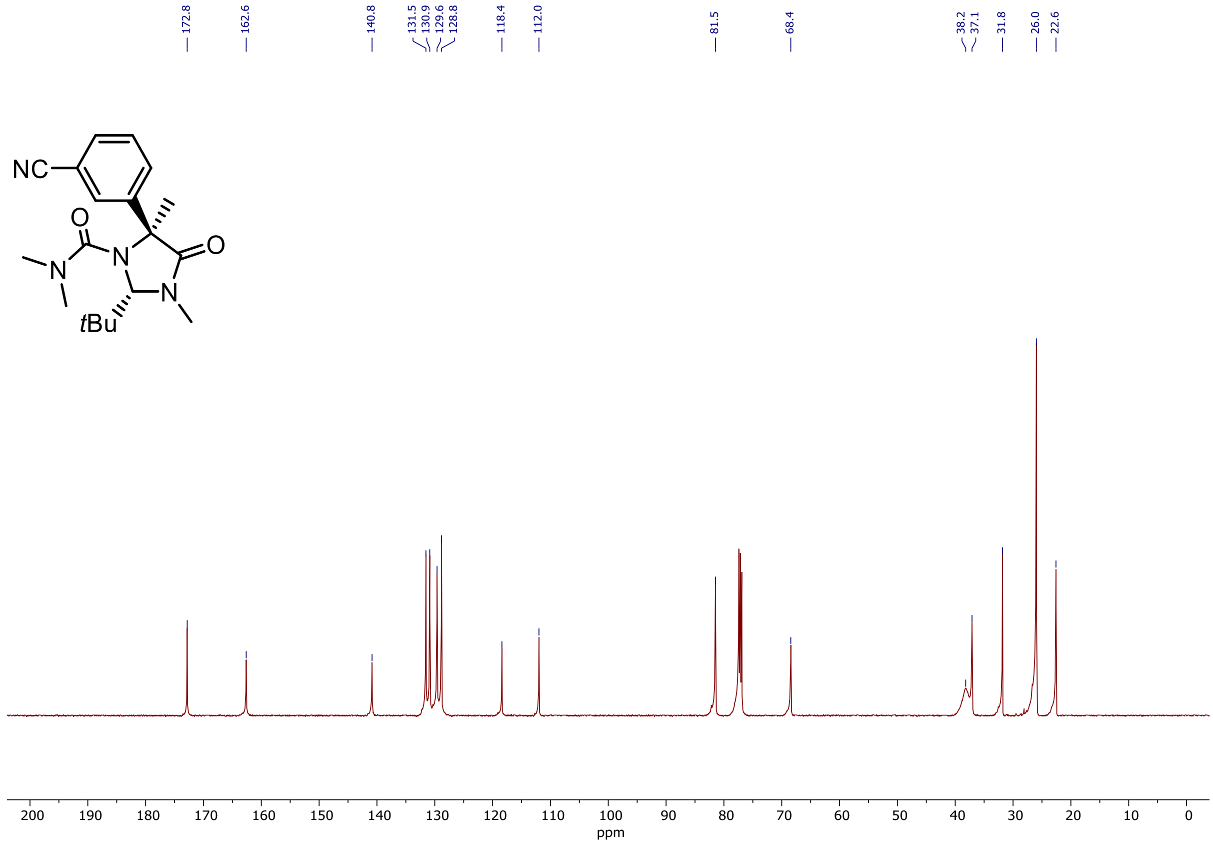

(2*S*,5*S*)-2-(*tert*-Butyl)-5-(3,5-difluorophenyl)-*N*,*N*,3,5-trimethyl-4-oxoimidazolidine-1-carboxamide (3d)

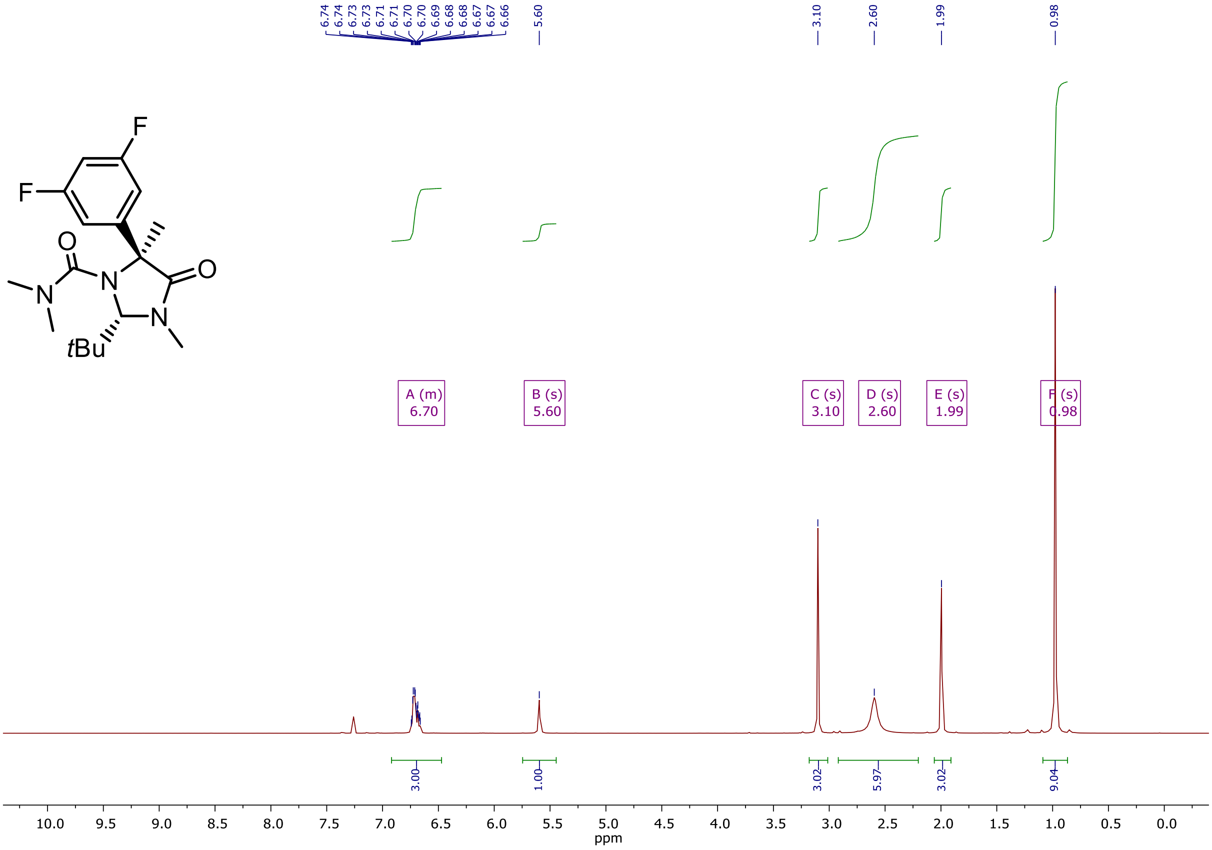

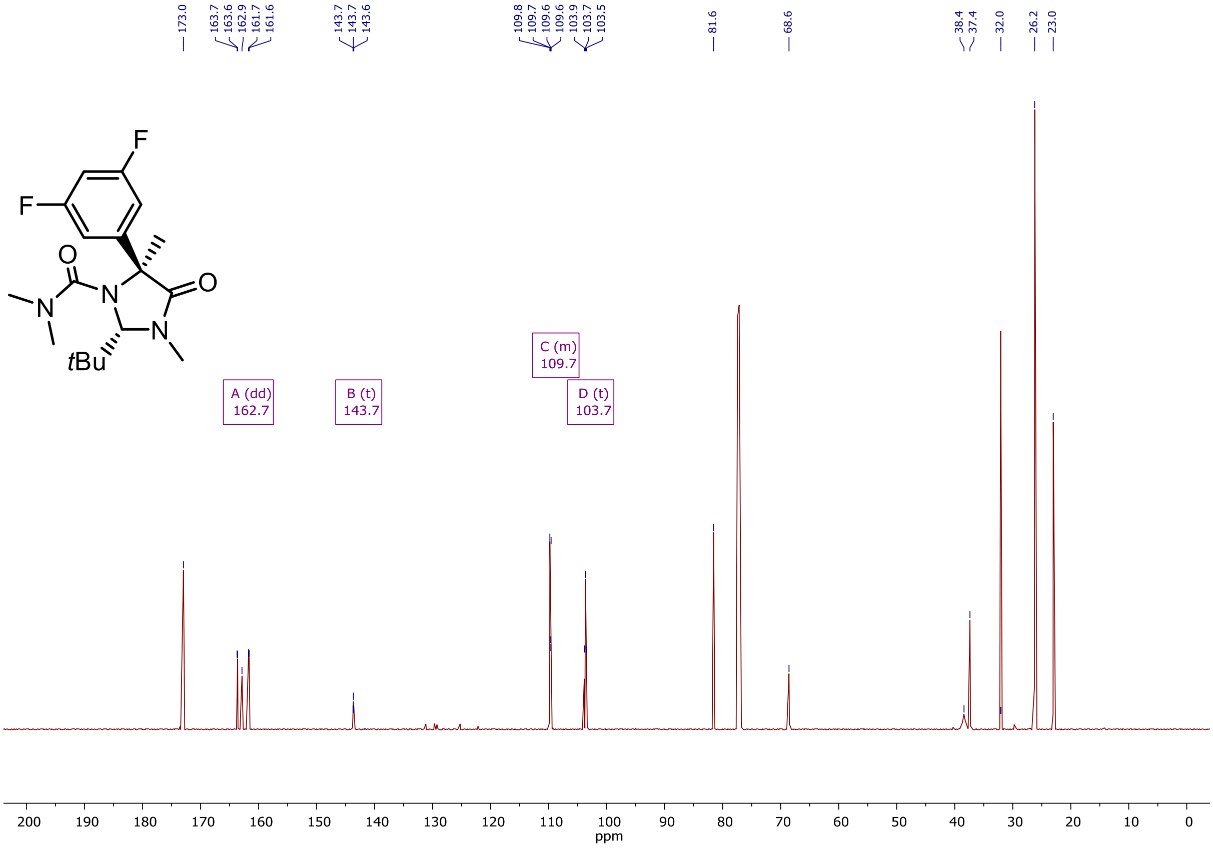

(2*S*,5*S*)-2-(*tert*-Butyl)-5-(3,5-dichlorophenyl)-*N*,*N*,3,5-trimethyl-4-oxoimidazolidine-1-carboxamide (3e)

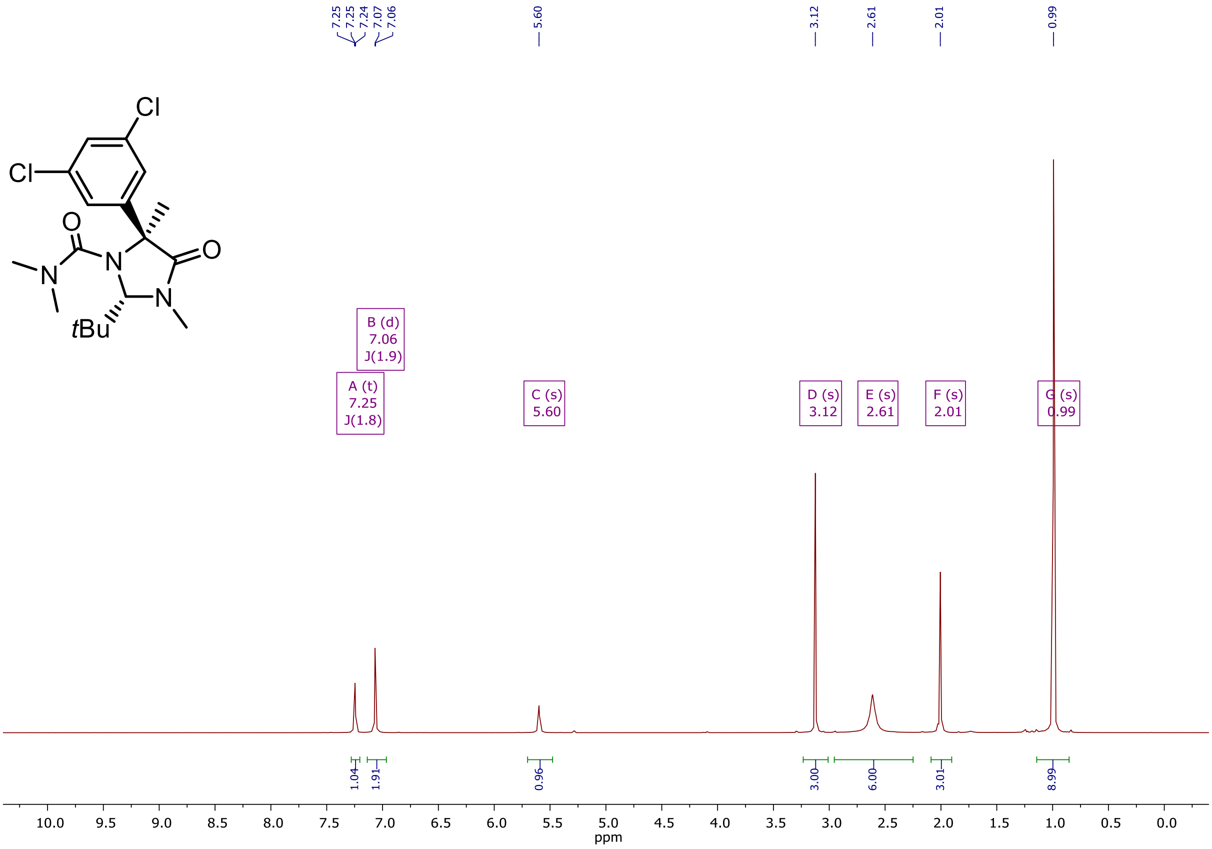

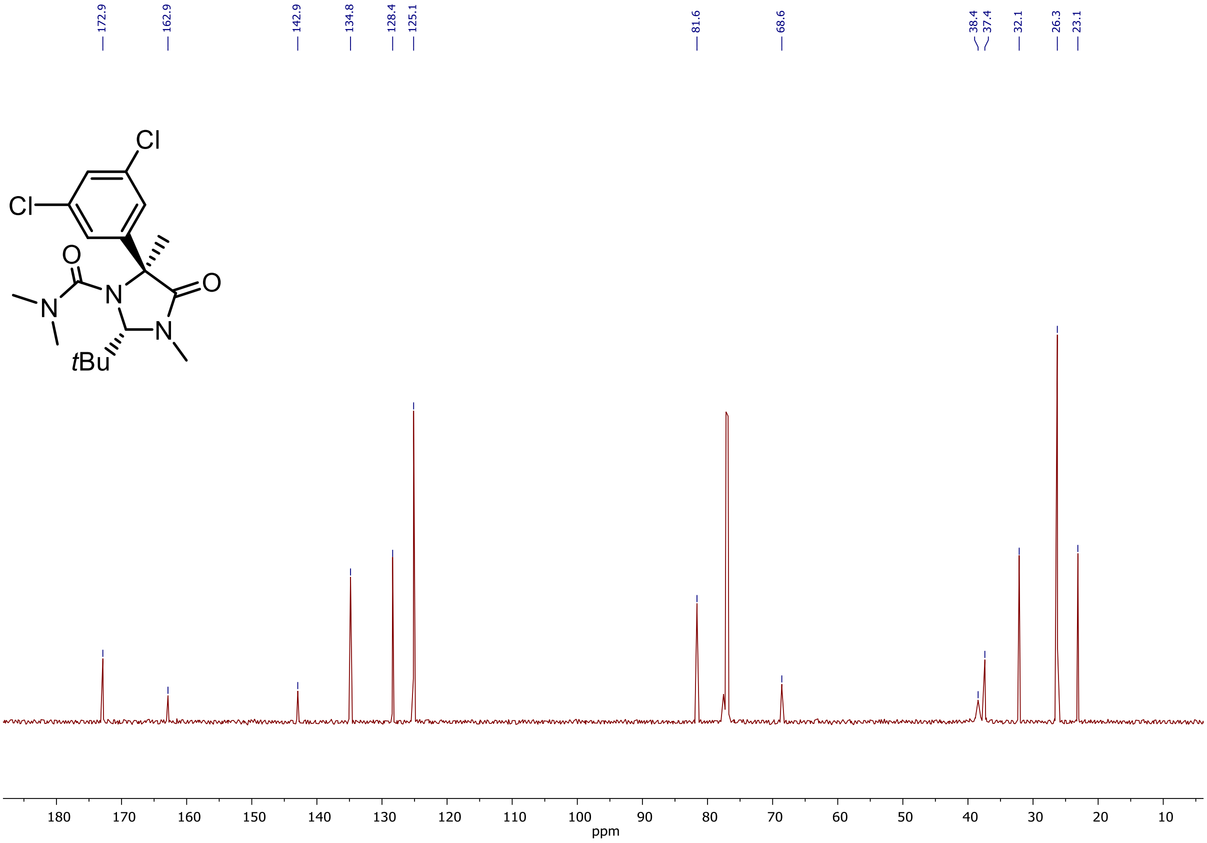

(2*S*,5*S*)-5-Benzyl-2-(*tert*-butyl)-5-(4-chlorophenyl)-*N*,*N*,3-trimethyl-4-oxoimidazolidine-1-carboxamide (3f)

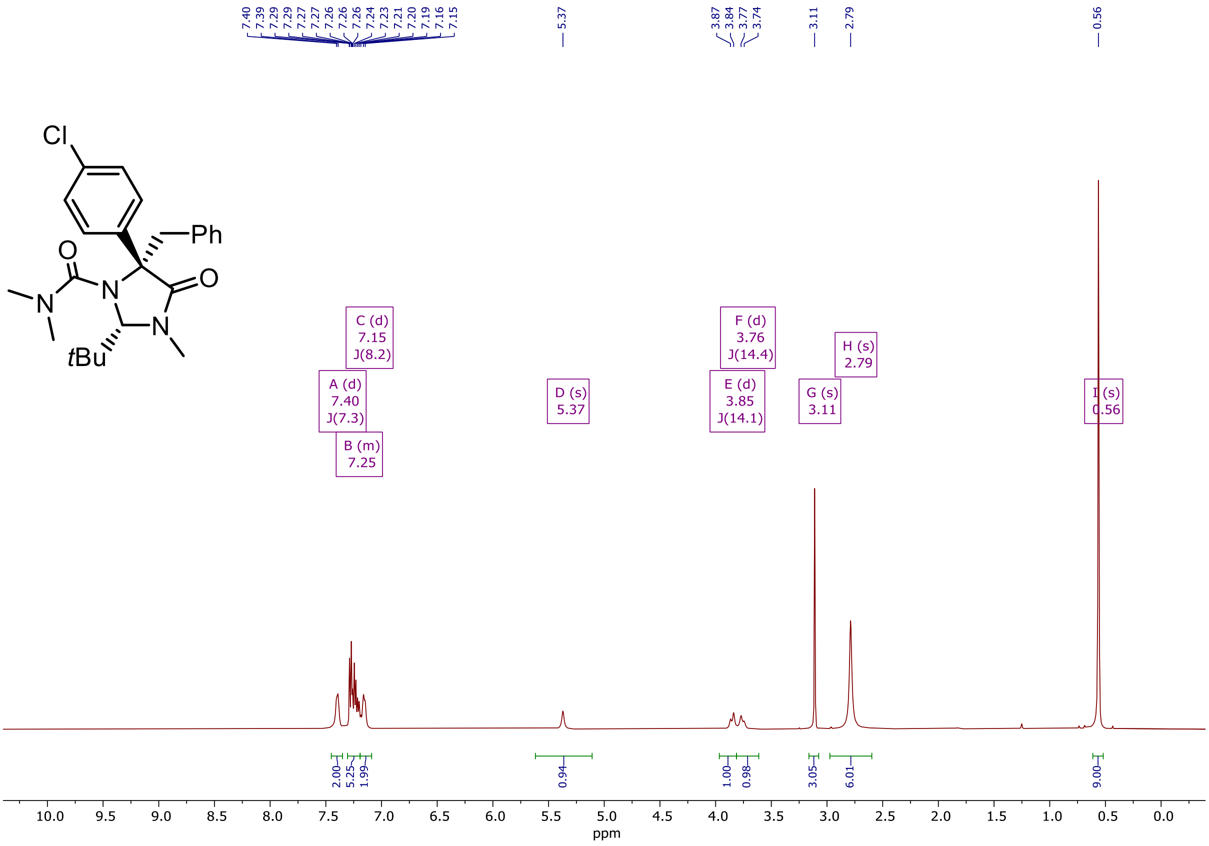

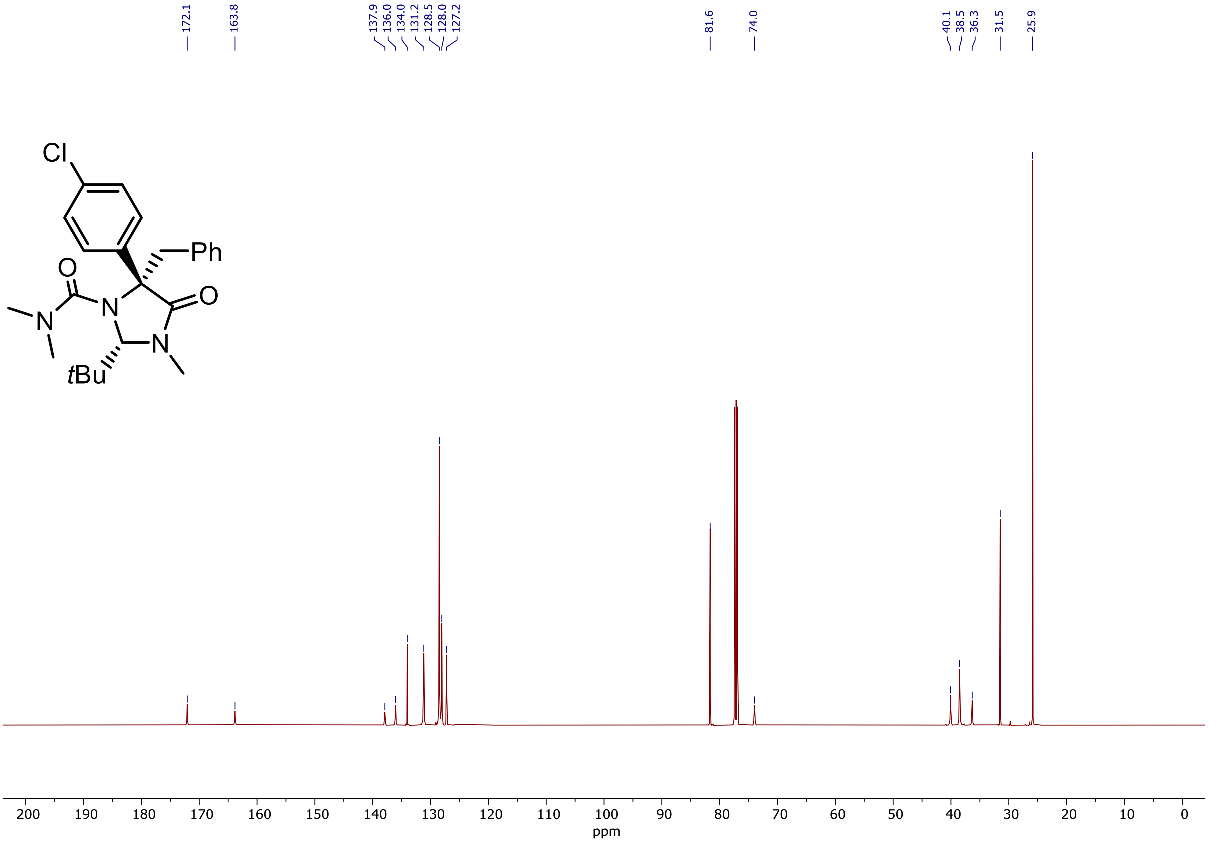

(2*S*,5*S*)-5-Benzyl-2-(*tert*-butyl)-*N*,*N*,3-trimethyl-4-oxo-5-(*p*-tolyl)imidazolidine-1-carboxamide (3g)

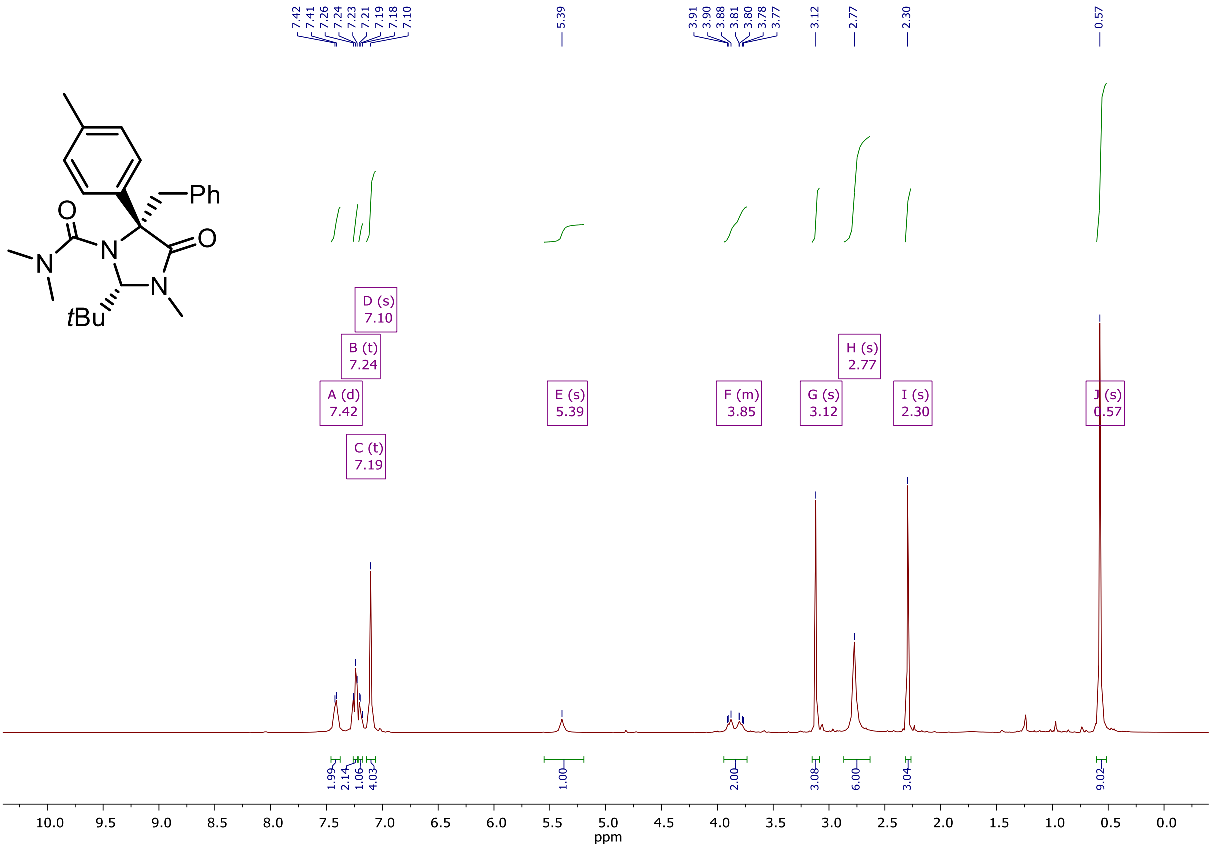

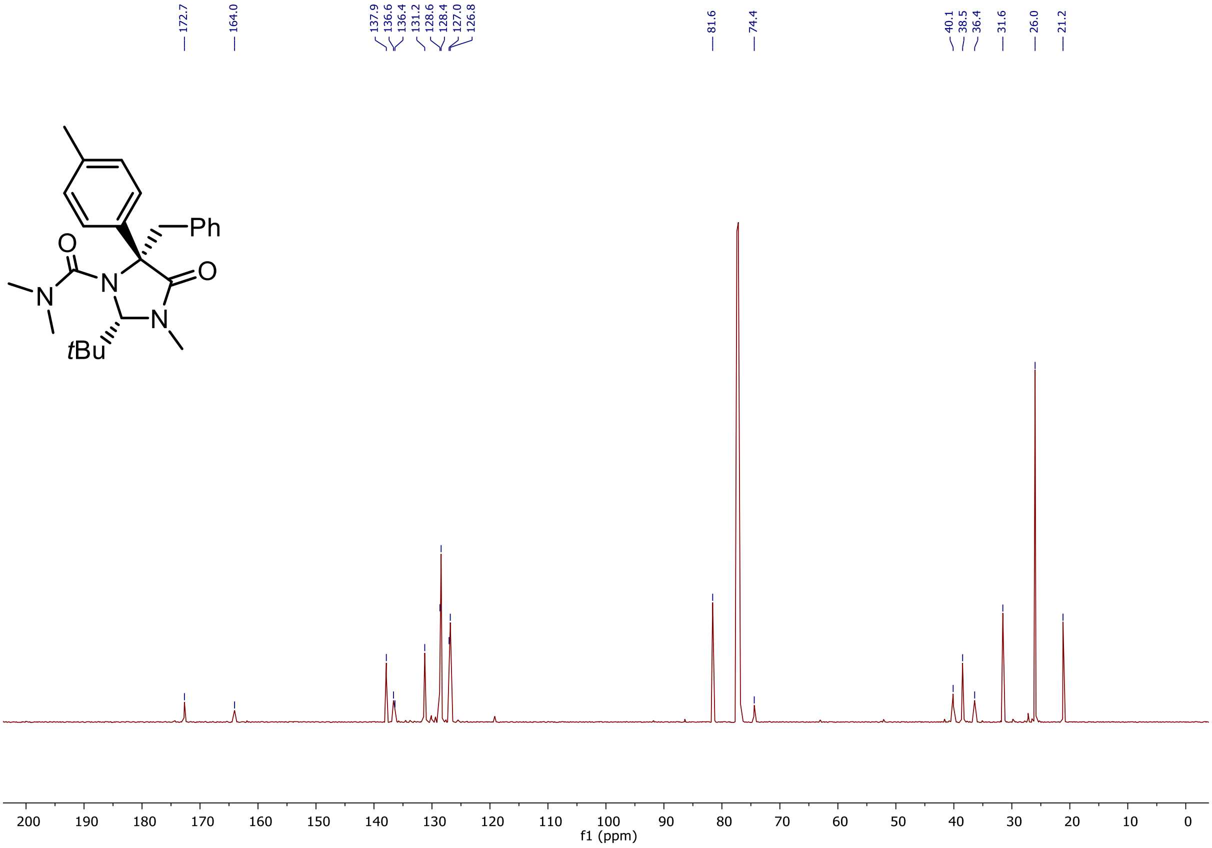

(2*S*,5*S*)-5-Benzyl-5-(3-bromophenyl)-2-(*tert*-butyl)-*N*,*N*,3-trimethyl-4-oxoimidazolidine-1-carboxamide (3h)

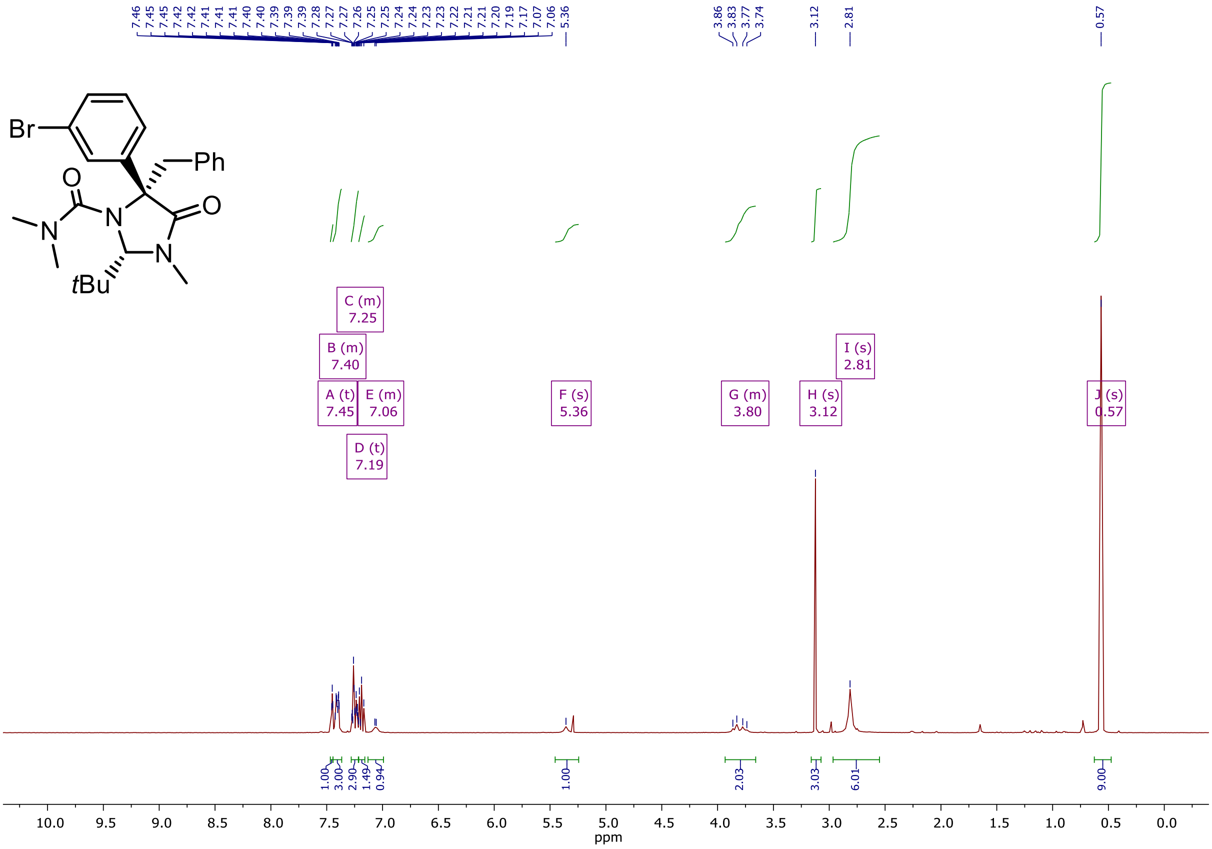

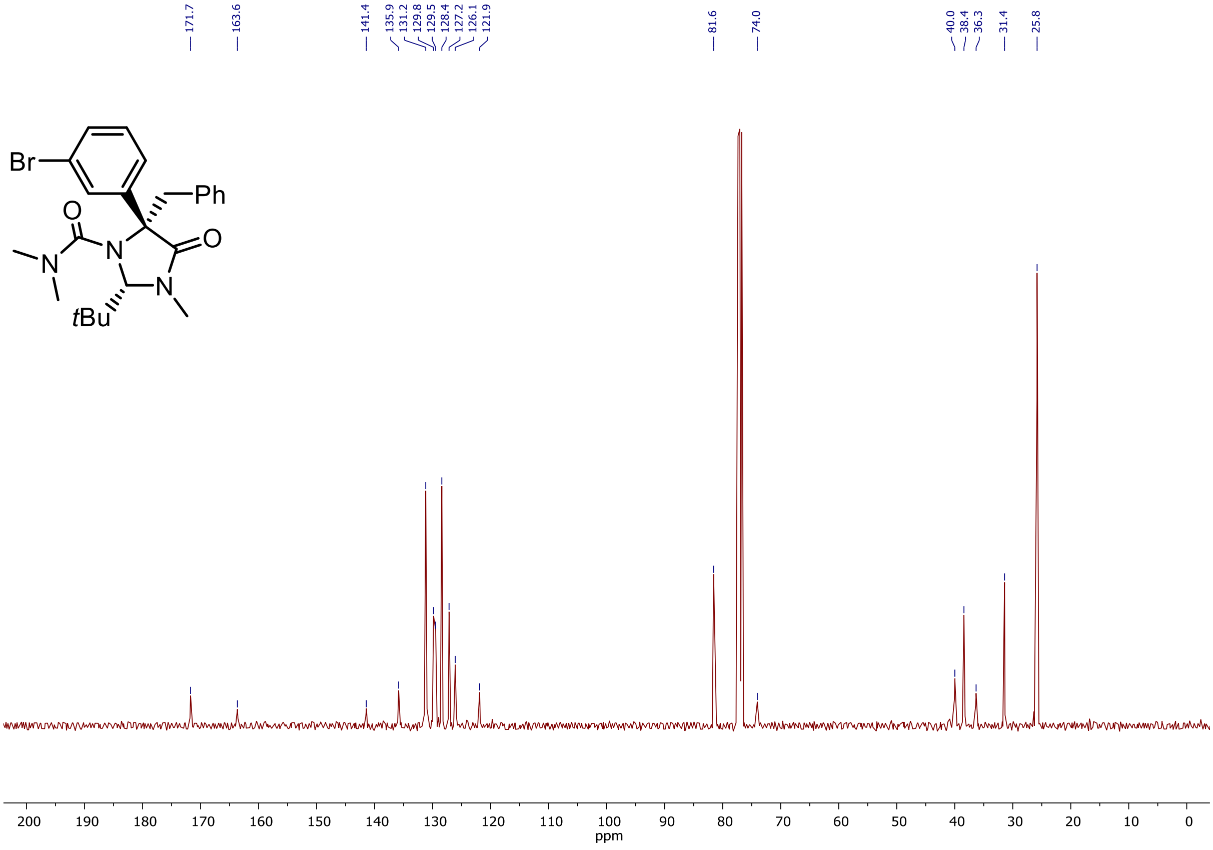

(2*S*,5*S*)-5-(4-(Benzyloxy)benzyl)-2-(*tert*-butyl)-*N*,*N*,3-trimethyl-4-oxo-5-(*p*-tolyl)imidazolidine-1-carboxamide (3i)

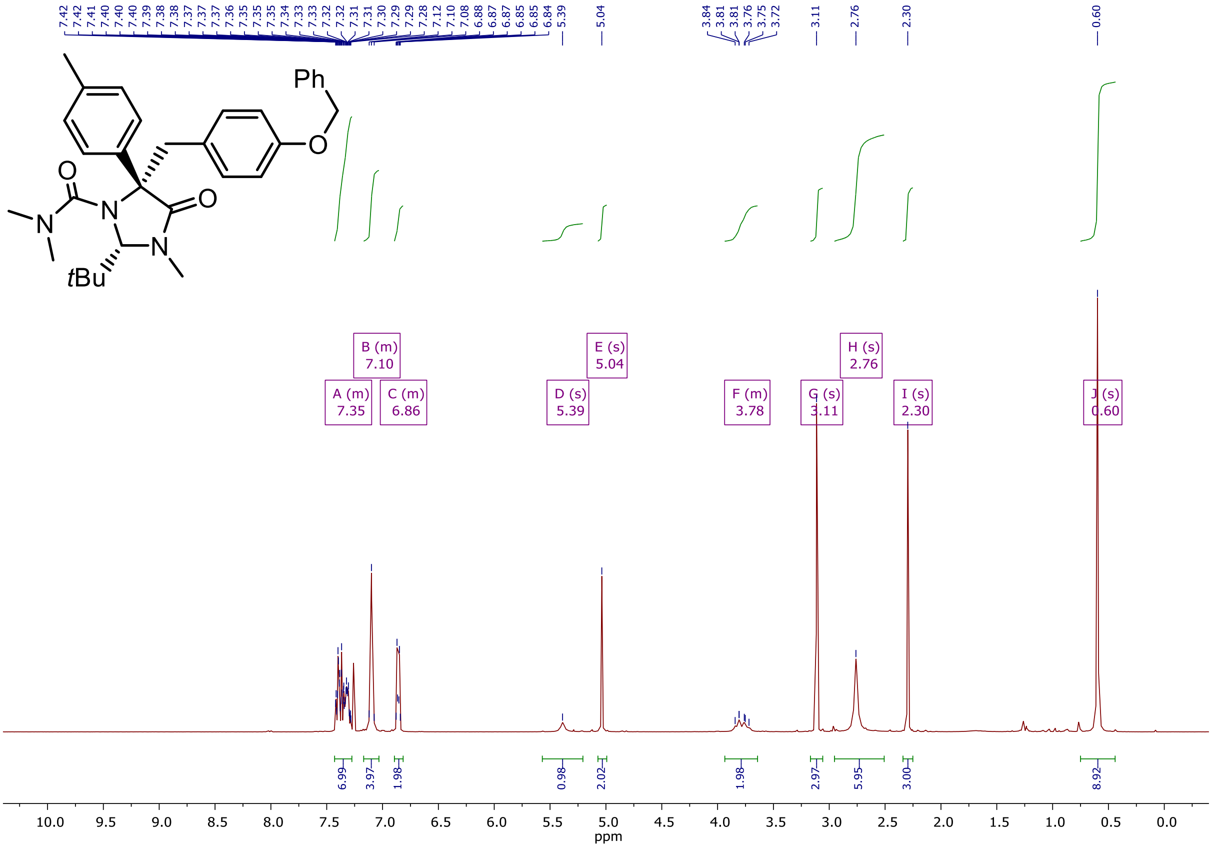

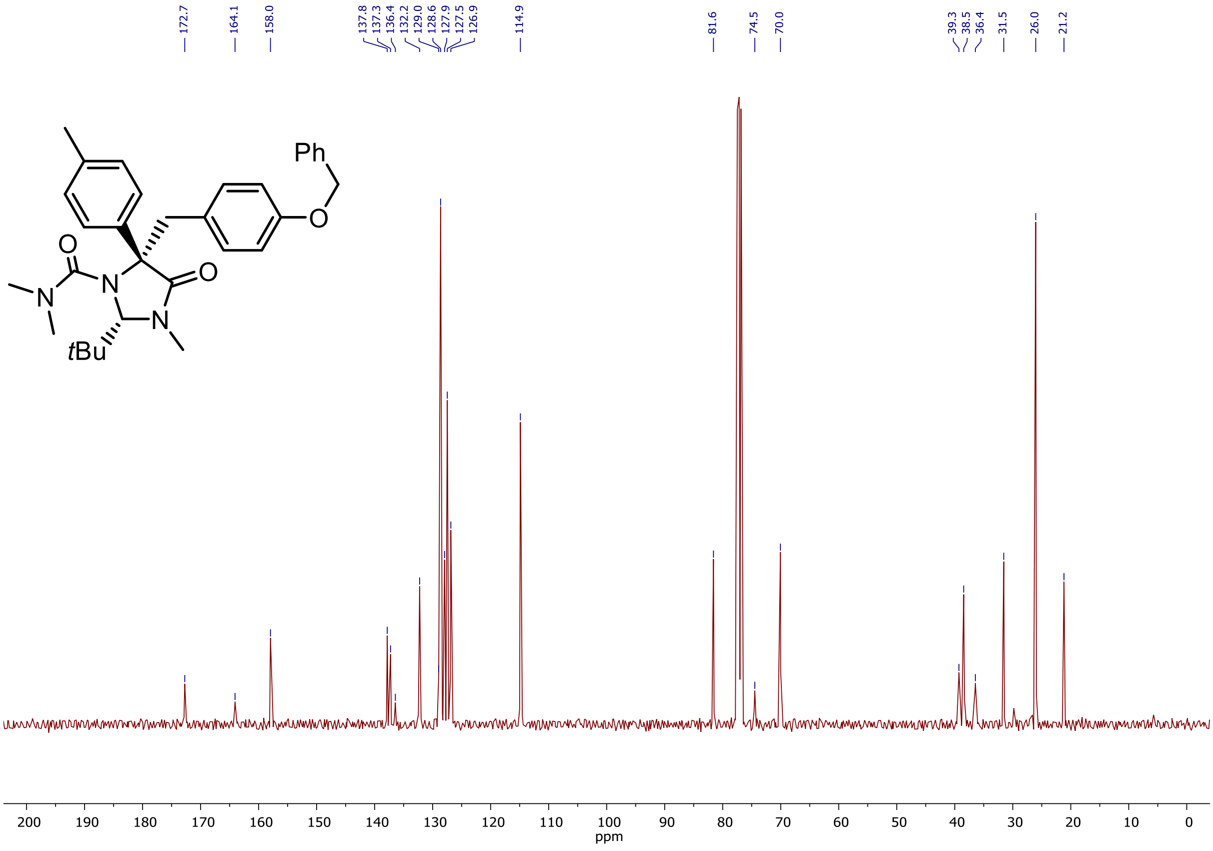

(2*S*,5*S*)-5-(4-(Benzyloxy)benzyl)-5-(4-bromo-3-fluorophenyl)-2-(*tert*-butyl)-*N*,*N*,3-trimethyl-4-oxoimidazolidine-1-carboxamide (3j)

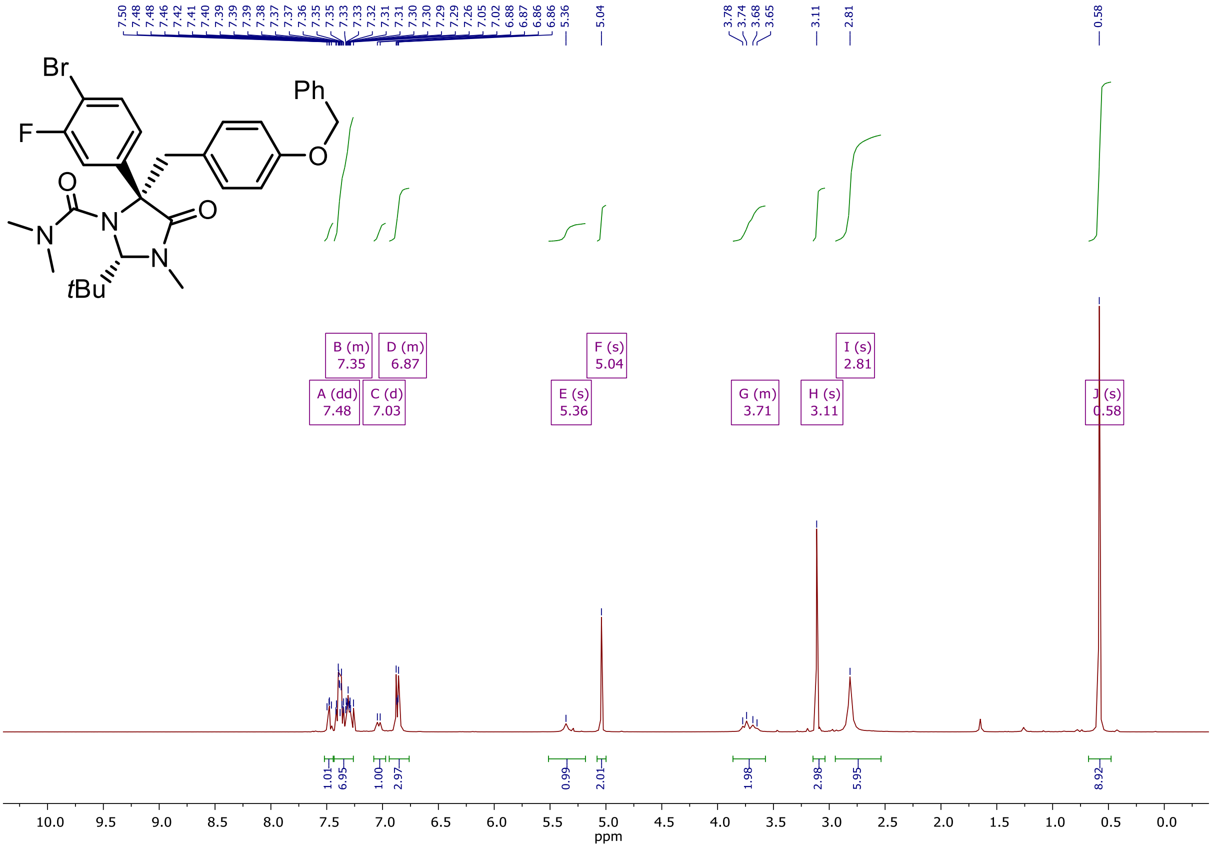

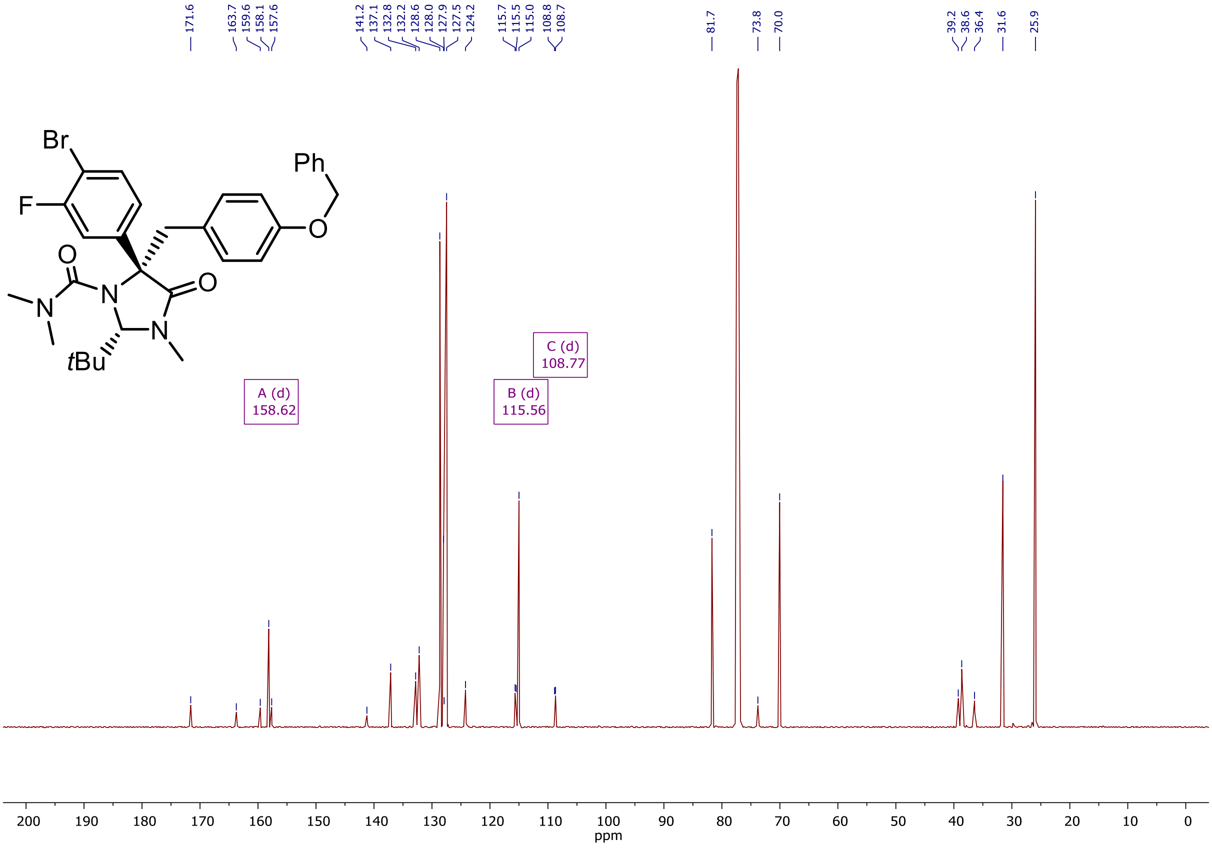

(*R*)-2-((((9*H*-Fluoren-9-yl)methoxy)carbonyl)amino)-2-(4-bromophenyl)propanoic acid (*R*-4a)

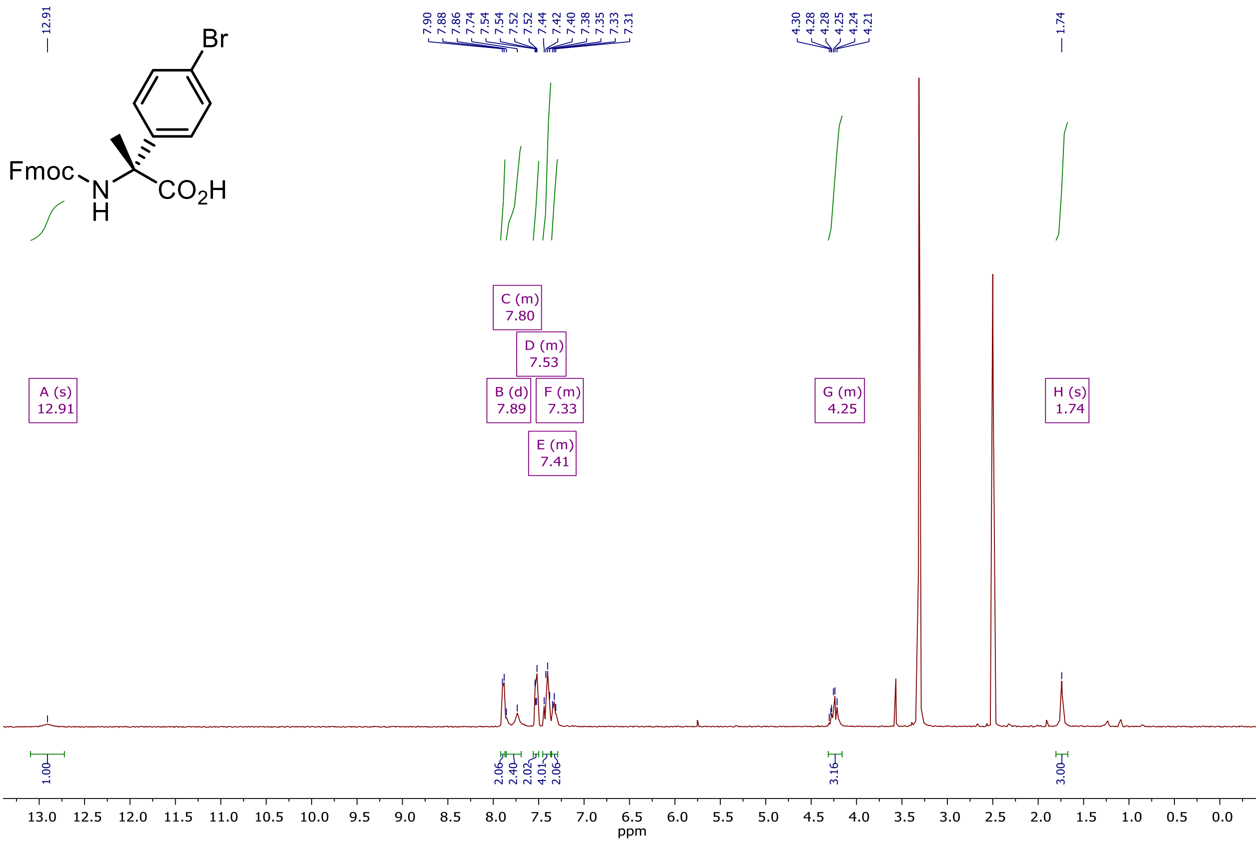

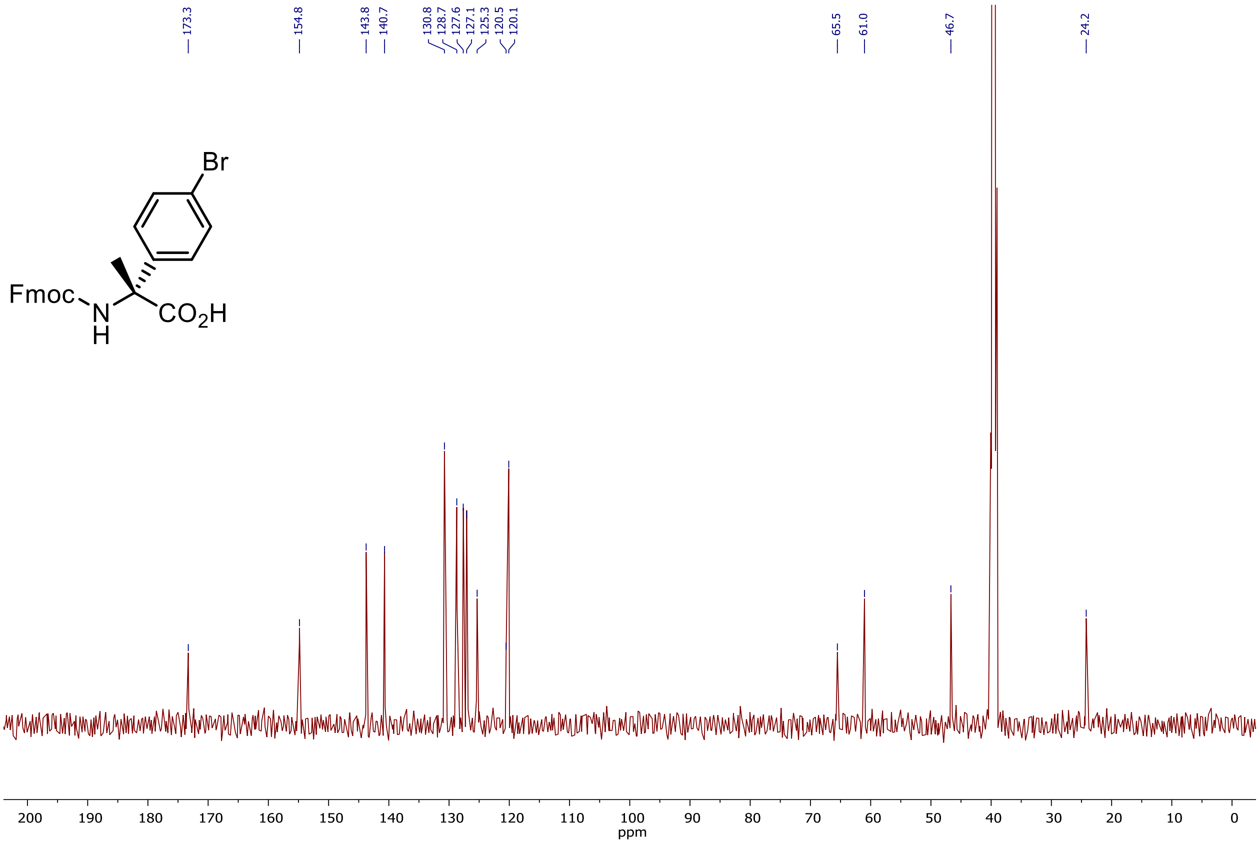

(*S*)-2-((((9*H*-Fluoren-9-yl)methoxy)carbonyl)amino)-2-(3-bromophenyl)propanoic acid (4b)

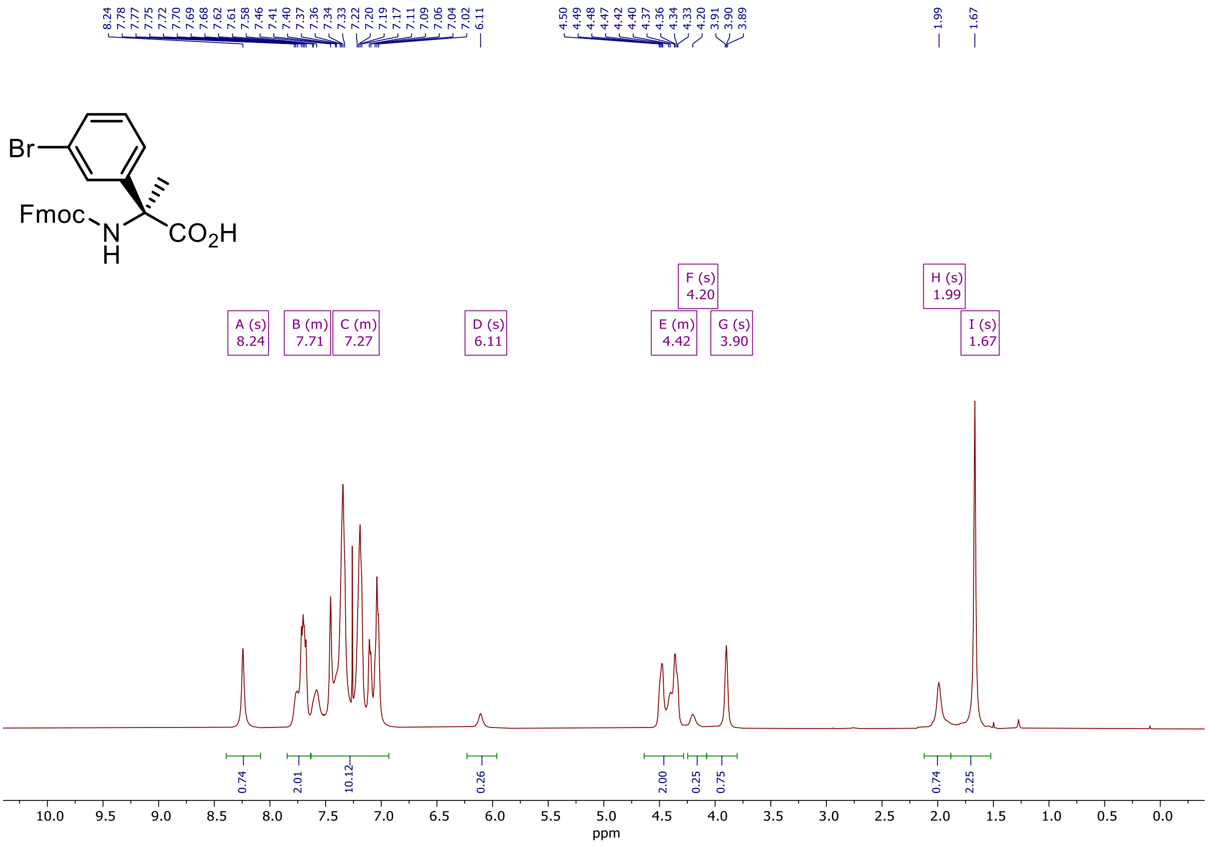

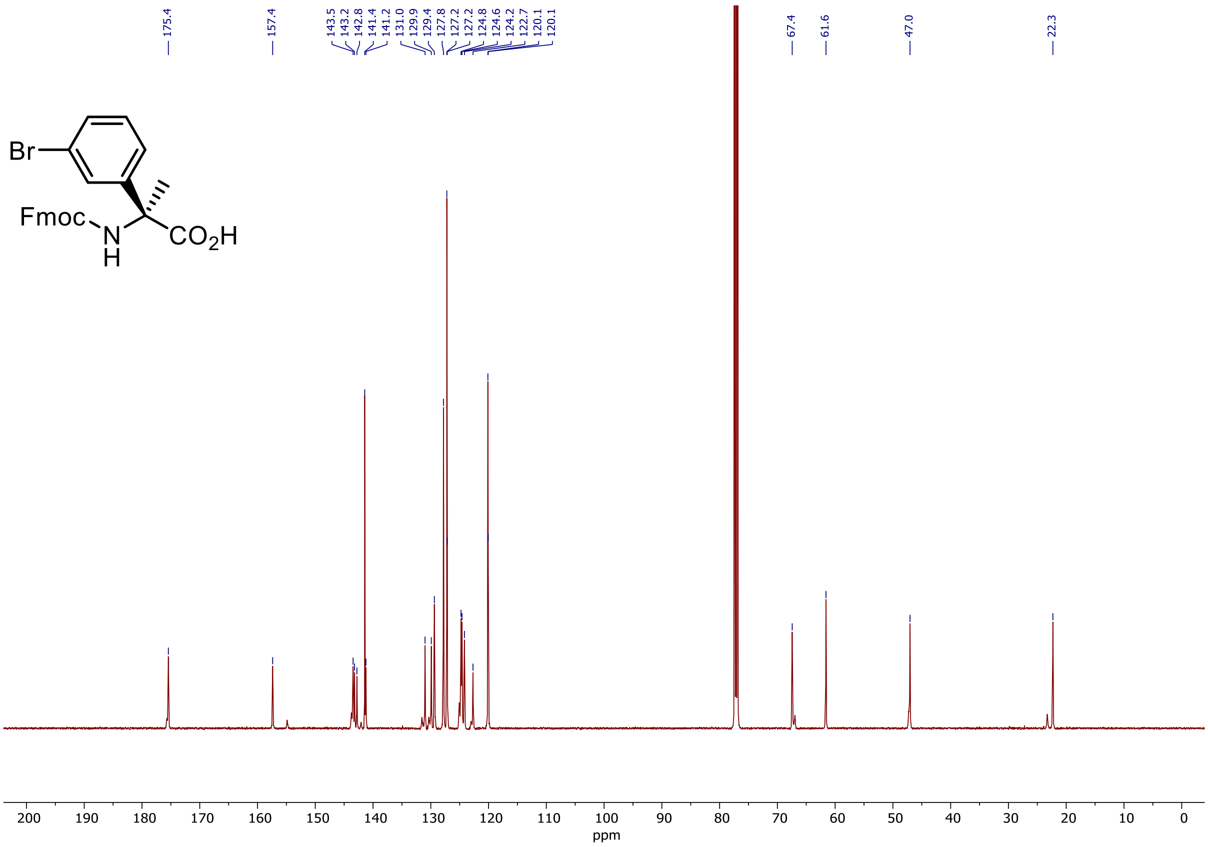

(*S*)-2-((((9*H*-Fluoren-9-yl)methoxy)carbonyl)amino)-2-(3,5-difluorophenyl)propanoic acid (4d)

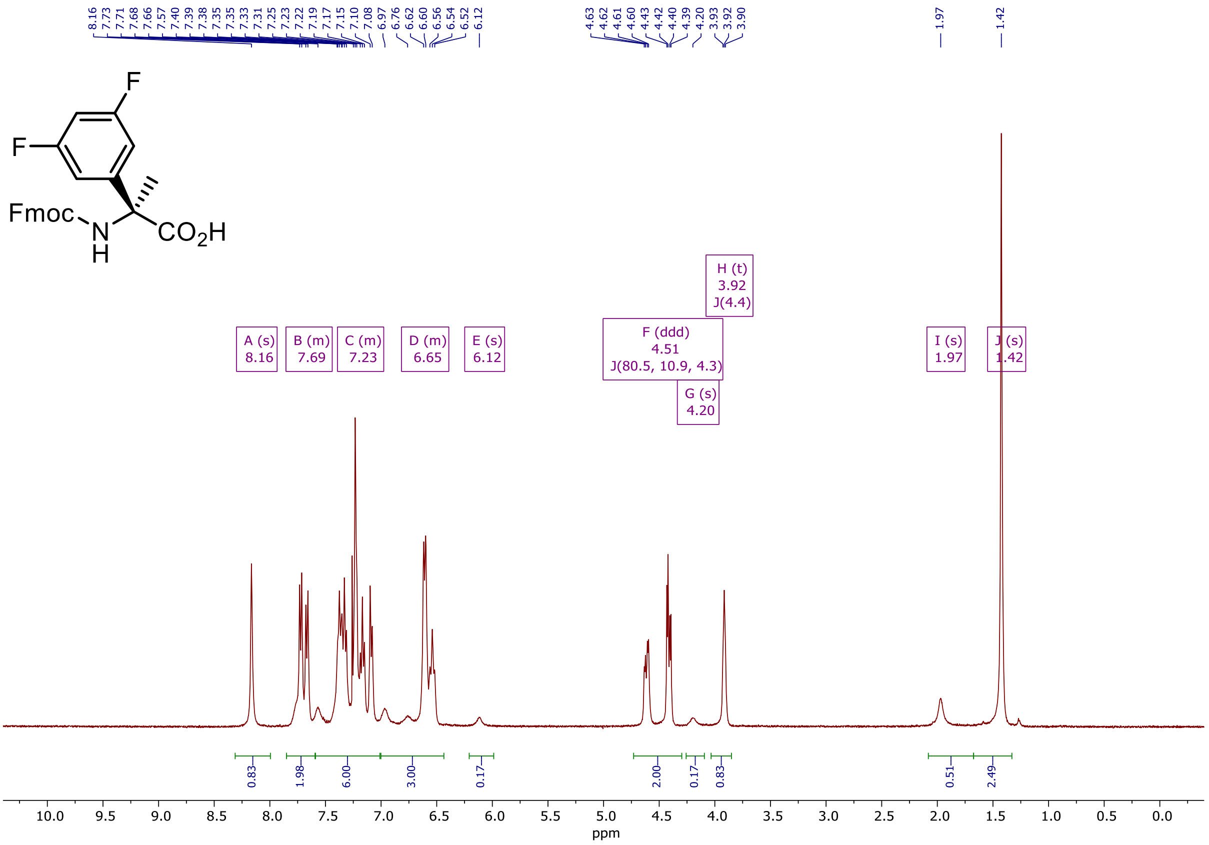

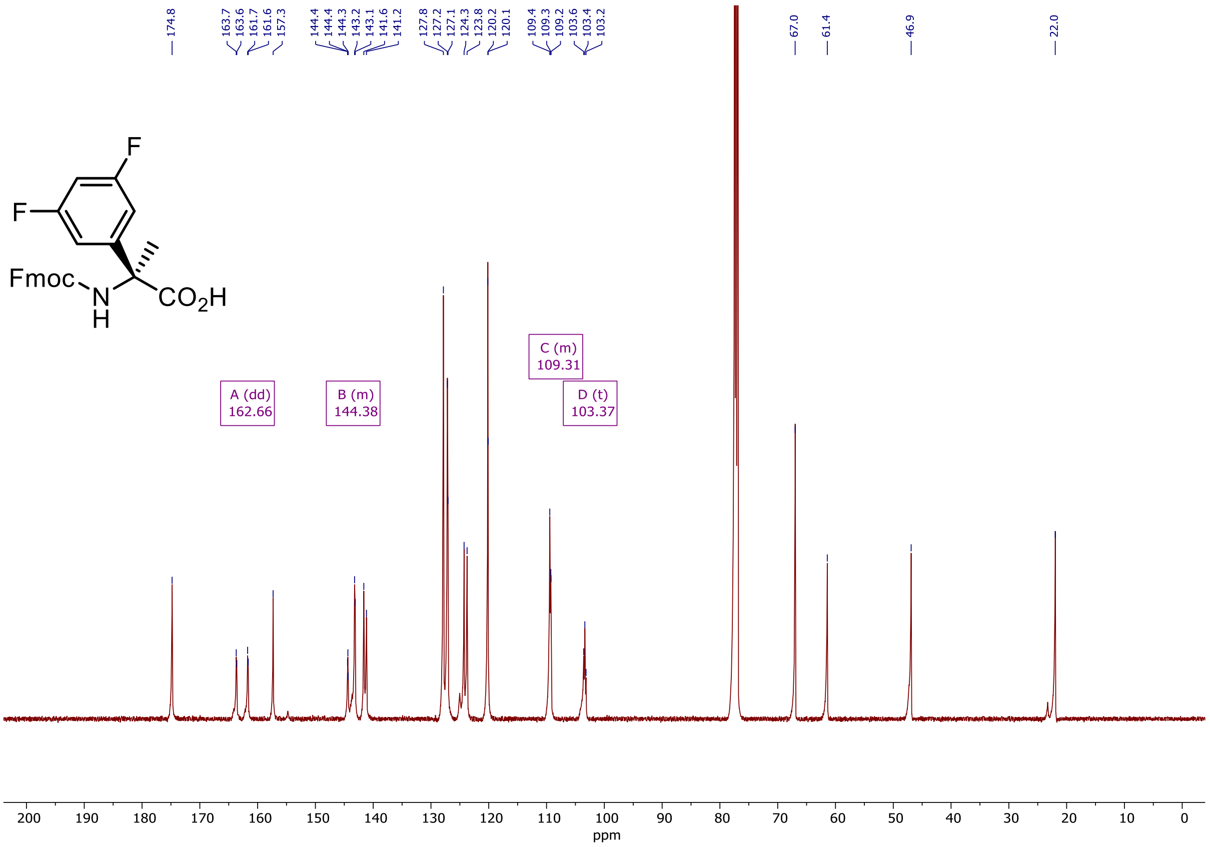

(*S*)-2-((((9*H*-Fluoren-9-yl)methoxy)carbonyl)amino)-2-(4-chlorophenyl)-3-phenylpropanoic acid (4f)

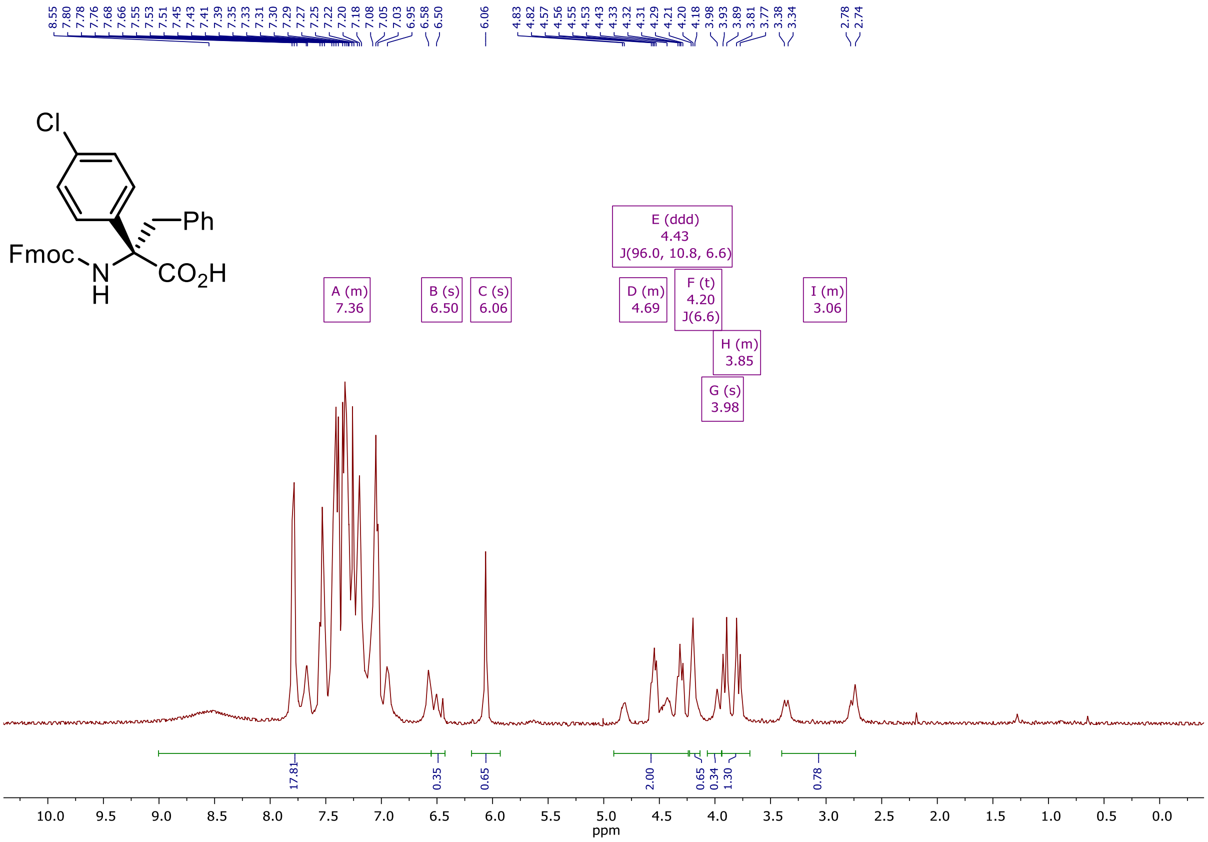

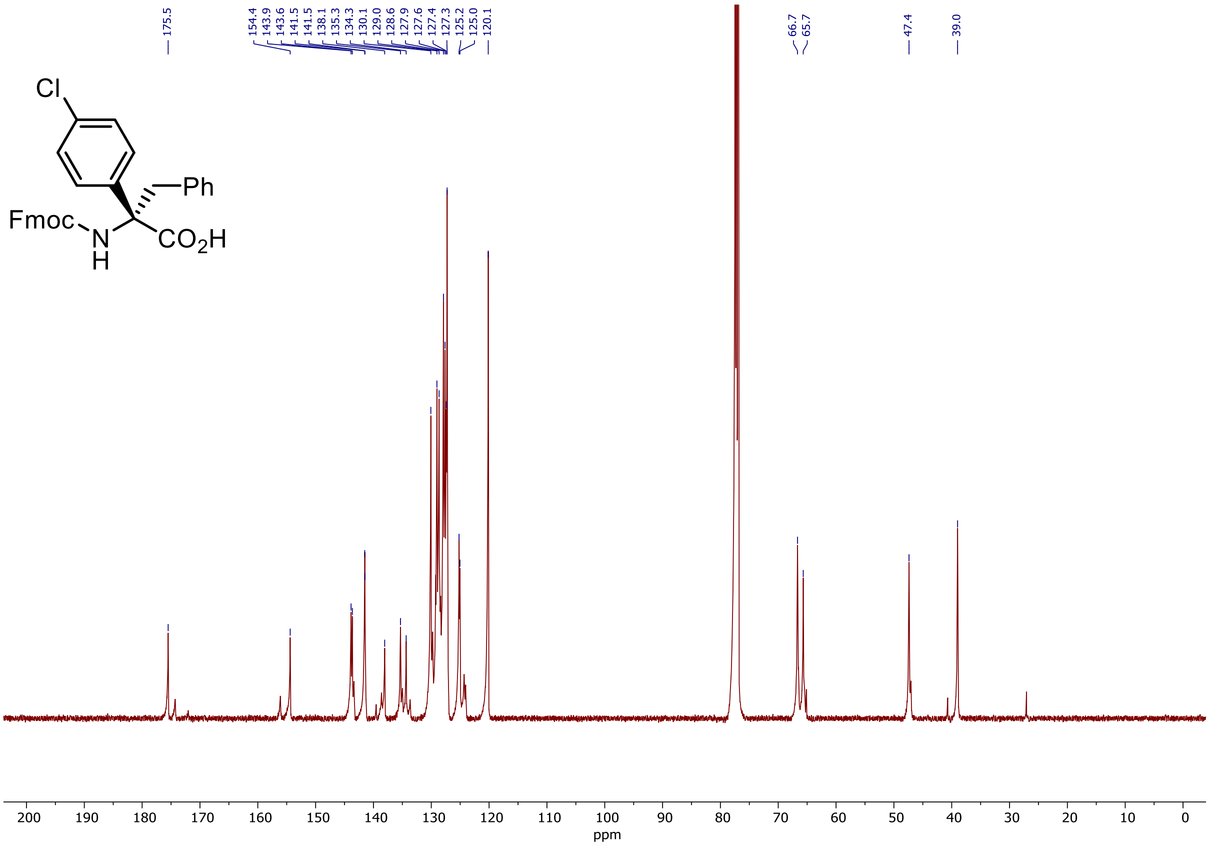

(*S*)-2-((((9*H*-Fluoren-9-yl)methoxy)carbonyl)amino)-3-phenyl-2-(*p*-tolyl)propanoic acid (4g)

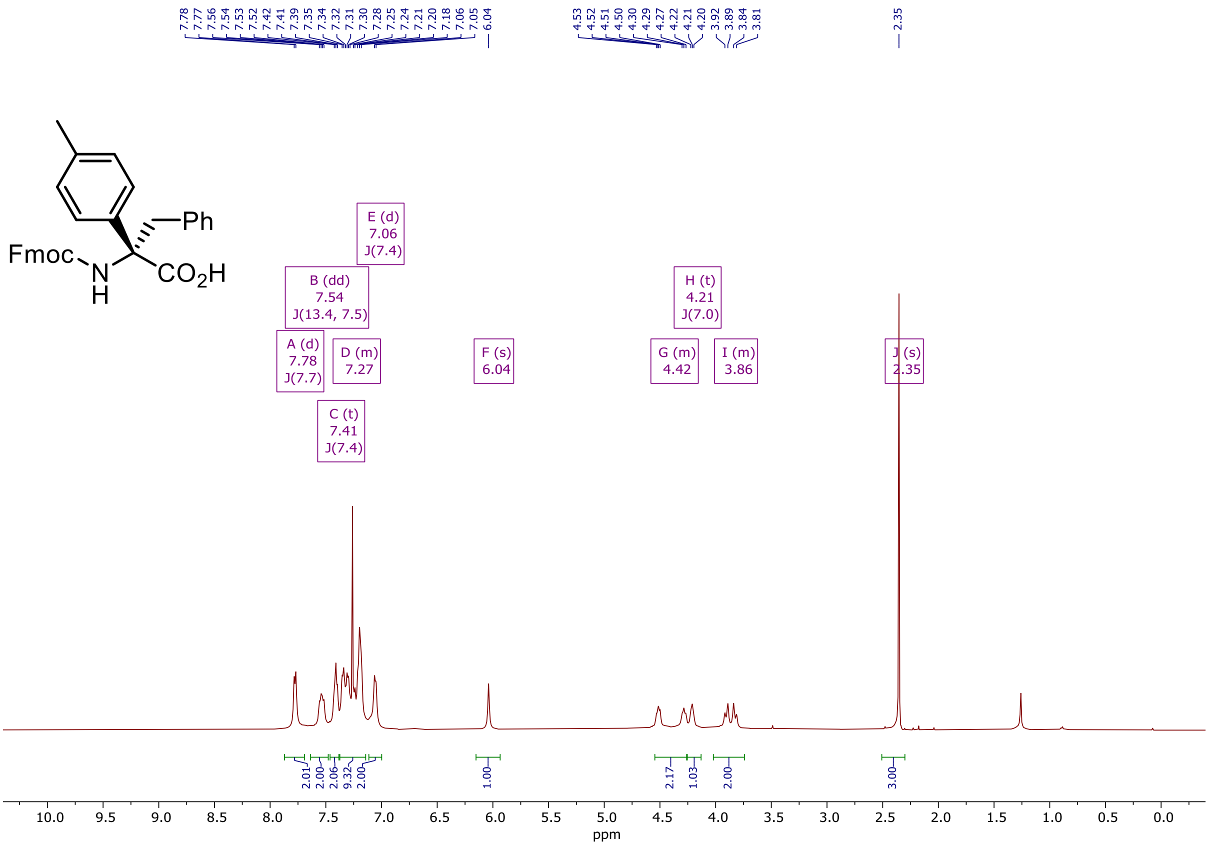

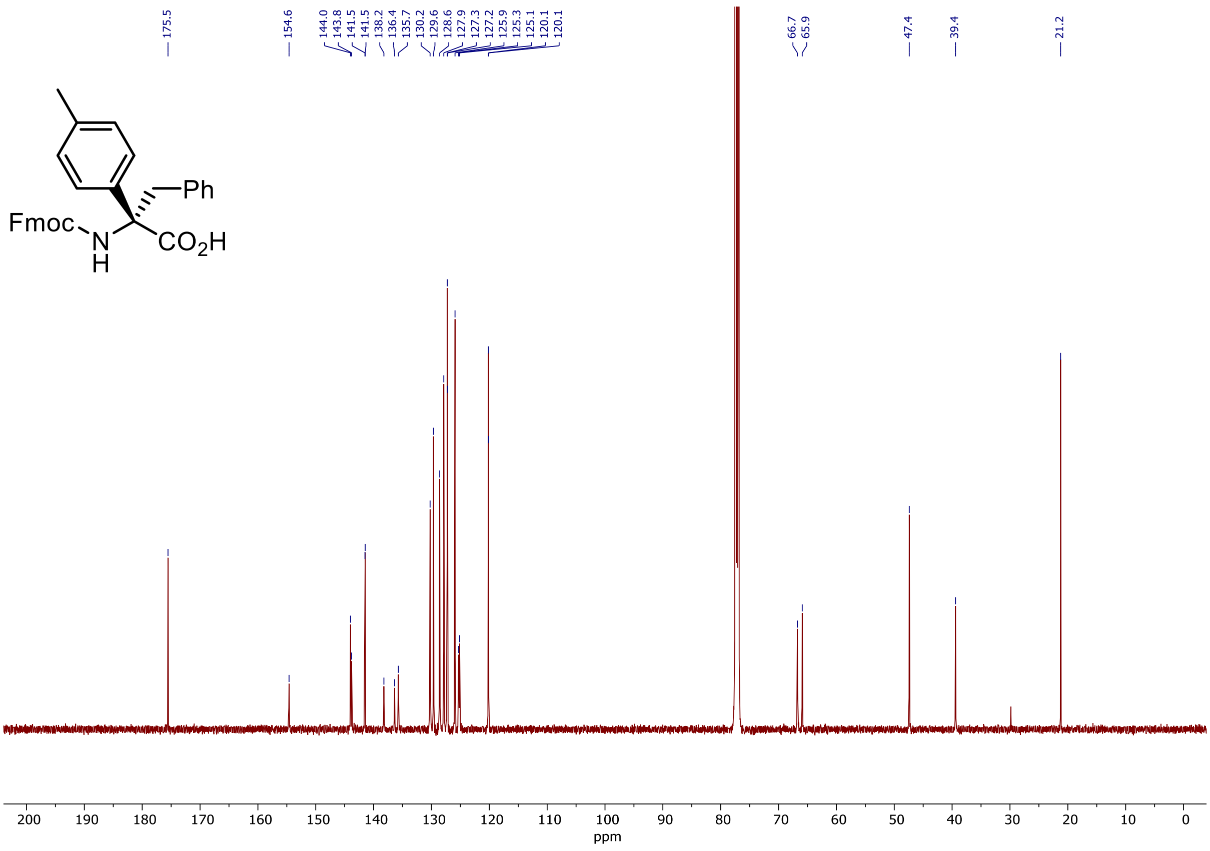

(*S*)-2-((((9*H*-Fluoren-9-yl)methoxy)carbonyl)amino)-2-(3-bromophenyl)-3-phenylpropanoic acid (4h)

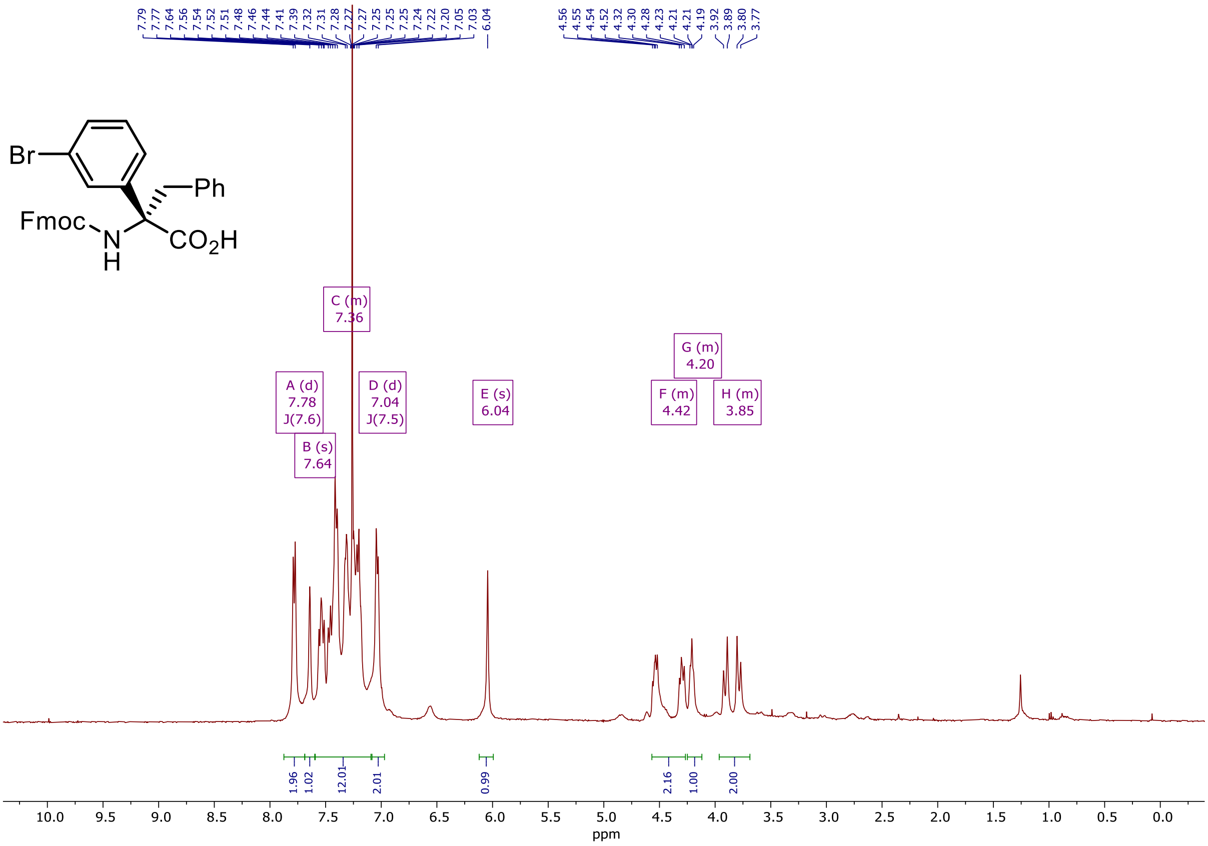

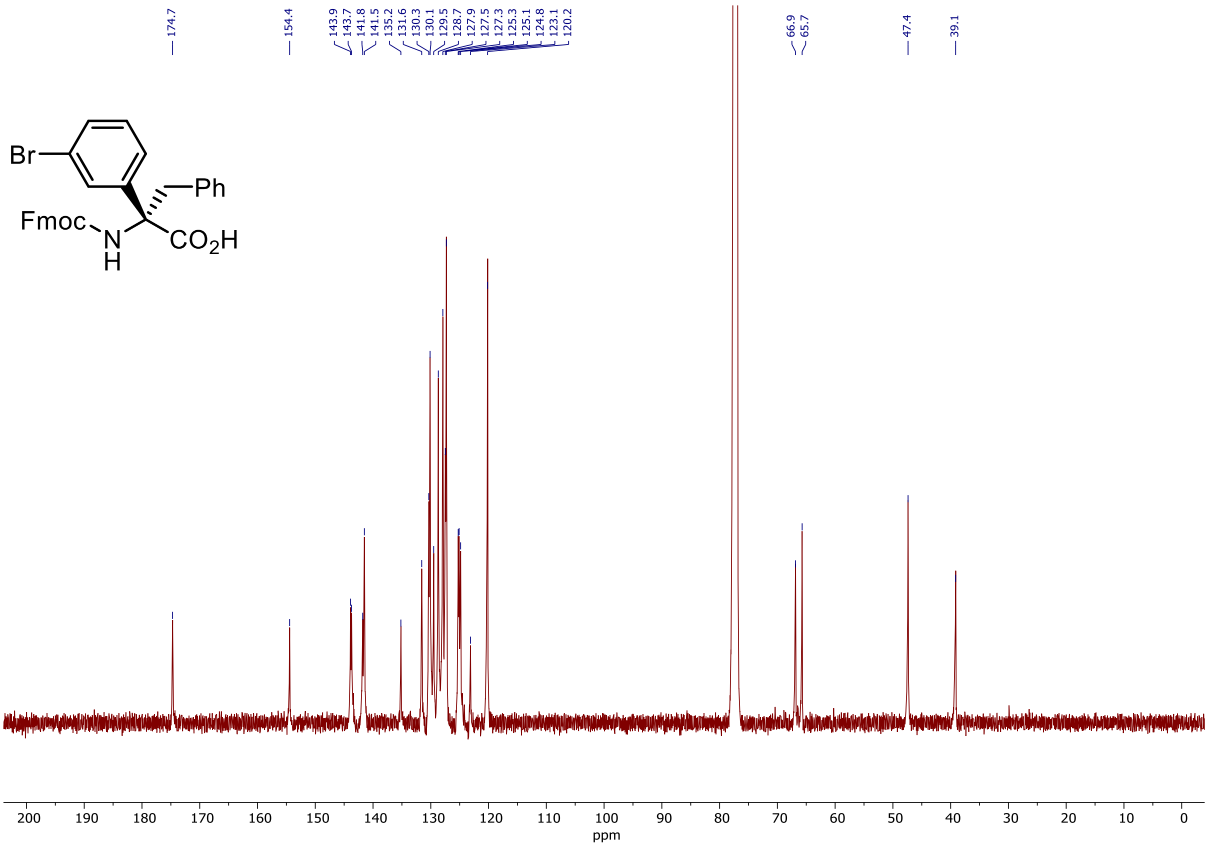

Peptide Synthesis

Peptides were synthesised using a microwave-assisted Liberty Blue automated peptide synthesizer (CEM Corporation) on Rink amide MBHA resin (Novabiochem, 0.51 mmol g^{-1}) as stated using Fmoc-coupling chemistry. All tertiary Fmoc-protected amino acids were purchased from Carbosynth Ltd (Compton, UK) or Fluorochem Ltd (Hadfield, UK). DMF was purchased from AGTC Bioproducts (Hessle, UK). Morpholine was purchased from Merck Millipore (Burlington, USA). All other chemicals were purchased from Sigma Aldrich (Gillingham, UK) or Fisher Scientific (Loughborough, UK) and used without further purification.

Table S1: Coupling cycle for quaternary amino acid or any standard amino acid following a quaternary residue (0.1 mmol scale).

Operation	Reagent(s)	Volume (mL)	Time (min)	Temp (°C)
Fmoc Deprotection	20% Morpholine in DMF	7.0	2	90
Drain and Wash (x3)	DMF	4.0, 5.0 & 4.0	-	-
Fmoc Deprotection	20% Morpholine in DMF	7.0	2	90
Drain and Wash (x3)	DMF	4.0, 5.0 & 4.0	-	-
Add amino acid	0.2 M Fmoc-AA-OH in DMF	2.5	-	-
Add DIC	1.0 M DIC in DMF	1.0	-	-
Add Oxyma	0.5 M Oxyma in DMF	1.0	-	-
Coupling	-	-	10	100
Drain and Wash (x3)	DMF	2.0, 2.0 & 3.0	-	-

Table S2: Coupling cycle for any standard amino acid not following a quaternary residue (0.1 mmol scale).

Operation	Reagent(s)	Volume (mL)	Time (min)	Temp (°C)
Fmoc Deprotection	20% Morpholine in DMF	7.0	2	90
Drain and Wash (x3)	DMF	4.0, 5.0 & 4.0	-	-
Fmoc Deprotection	20% Morpholine in DMF	7.0	2	90
Drain and Wash (x3)	DMF	4.0, 5.0 & 4.0	-	-
Add amino acid	0.2 M Fmoc-AA-OH in DMF	2.5	-	-
Add DIC	1.0 M DIC in DMF	1.0	-	-
Add Oxyma	0.5 M Oxyma in DMF	1.0	-	-
Coupling	-	-	3	90

Drain and Wash (x3)	DMF	2.0, 2.0 & 3.0	-	-

Table S3: Final Deprotection Cycle (0.1 mmol scale).

Operation	Reagent(s)	Volume (mL)	Time (min)	Temp (°C)
Fmoc Deprotection	20% Morpholine in DMF	5.0	1	90
Drain and Wash (x3)	DMF	5.0, 4.0 & 4.0	-	-
Fmoc Deprotection	20% Morpholine in DMF	5.0	1	90
Drain and Wash (x3)	DMF	5.0, 4.0 & 4.0	-	-

Peptide acetylation and cleavage took place in a fritted syringe with a stopcock tap. *N*-acetylation was carried out using excess Ac_2O (1 mL) and DIPEA (2 mL) in DMF (7 mL) with inversion for 30 mins. Cleavage from the resin was carried out with CH_2Cl_2:TFA:H_2O:TIPS (45:45:5:5 vol%, 15 mL) for peptide 1 and TFA:H_2O:TIPS (90:5:5 vol%, 15 mL) for peptides 2 & 3 with inversion for 1 and 3 h respectively. Following cleavage, the TFA solution was reduced to ~5 mL under a flow of nitrogen. The crude peptides were precipitated by cold Et_2O (~30 mL) and isolated by refrigerated centrifugation (3000 rpm, 10 mins), the precipitate dissolved in 1:1 MeCN:H_2O solution (5 mL), and lyophilized to give a white powder.

Peptide Purification

Crude peptides were purified by reverse-phase HPLC on a JASCO HPLC system equipped with a Phenomenex Luna C18 column (5 µm particle size; 100 Å pore size; 150×10 mm). A gradient of water (0.1 % TFA, buffer A) and acetonitrile (0.1 % TFA, buffer B) between 20 and 80 % or 40 and 100 % buffer B over 30 min at a flow rate of 3 mL min^{-1} with absorbance recorded at 220 and 280 nm was typically used. The fractions collected from HPLC were analysed by matrix assisted laser desorption ionization time-of-flight mass spectrometry (MALDI-TOF MS) on a Bruker ultraFlexXtreme II MALDI-TOF mass spectrometer operating in positive-ion reflector mode. Peptides were co-crystallised on a ground-steel target plate using α-cyano-4-hydroxycinnamic acid as the matrix. Peptide purity was confirmed by reverse-phase analytical HPLC on a JASCO chromatography system fitted with a Phenomenex® Kinetex C18 (5 µM particle size; 100 x 4.5 mm) column.

Peptide Concentration Determination

Peptide concentrations were determined in H_2O by UV-vis absorption at 280 nm on a Nanodrop 2000 (Thermo Scientific) using the extinction coefficient for tryptophan: ε_{280} = 5690 dm^3 mol^{-1} cm^{-1}. Any contribution to this from the arylated amino acids were deemed negligible.

Circular Dichroism Spectroscopy

Circular dichroism (CD) spectra were measured at 5 °C as the average of eight scans from 260–190 nm using a JASCO 810 spectropolarimeter fitted with a Peltier temperature controller, a 1 mm pathlength quartz cuvette (Starna), a scanning speed of 100 nm min^{-1}, and a bandwidth of 1 nm. Peptides were prepared at 50 µM concentration (150 µL) in phosphate-buffered saline (PBS, 8.2 mM Na_2HPO_4, 1.8 mM KH_2PO_4, 2.7 mM KCl, 137 mM NaCl, pH 7.4). Thermal unfolding profiles were obtained from 5 to 95 °C with a temperature slope of 60 °C h^{-1} by monitoring the absorbance at 222 nm (1 nm bandwidth) and 1 °C intervals with 16 s delay and 16 s response times. The midpoint of the denaturation curve was determined (T_M) as the maximum value for the first derivative of the thermal transition. Spectra in all cases are the averaged results of three replicate experiments.

Fractional helix percentage was calculated using the following equation:[5,6]

$$100 \times \frac{[\theta]_{222} - [\theta]_{coil}}{-42500 \times \left(1 - \frac{3}{n}\right) - [\theta]_{coil}}$$

Where $[\theta]_{coil}$ 415 deg cm^2 $dmol^{-1}$ res^{-1}at 5 °C, and n is the number of peptide bonds including the *N*-terminal acetyl.

Analytical Ultracentrifugation

Sedimentation equilibrium analytical ultracentrifugation (AUC) experiments were conducted at 20 °C in a Beckman XL-A Analytical Ultracentrifuge using an An-60 Ti rotor and Epon or aluminium 2-channel centrepieces fitted with quartz windows. Solutions were prepared at 75 µM (but 37.5 µM for peptide 15) in PBS to a volume of 100 µL for Epon centrepieces and 110 µL for aluminium centrepieces. The reference channel contained 110 µL of PBS for Epon centrepieces and 120 for aluminium centrepieces. The samples were centrifuged from 44–60 krpm in increments of 4 krpm. The absorbance was measured across the cell at a radial distance of 5.8–7.3 cm at each speed after 8 h and then again after a further 1 h to check the samples had reached equilibrium before moving onto the next speed. The data were fitted to a single ideal species model with sedphat (http://www.analyticalultracentrifugation.com/sedphat/download.htm). 95% confidence limits were calculated using Monte Carlo analysis of the obtained fits. The partial-specific volume ($\bar{v}$) for each of the various peptides and the solvent density (PBS, 1.0054 g mL^{-1}) were calculated using Sednterp (http://www.jphilo.mailway.com/download.htm). `b` and `B` were treated as Tyr in calculating $\bar{v}$.

Peptide Characterisation

CC-Mono (**8**)

Sequence: `Ac–G EAAAAKQ EAAAAKK EAAAAKW EAAAAKQ G-NH`$_2$

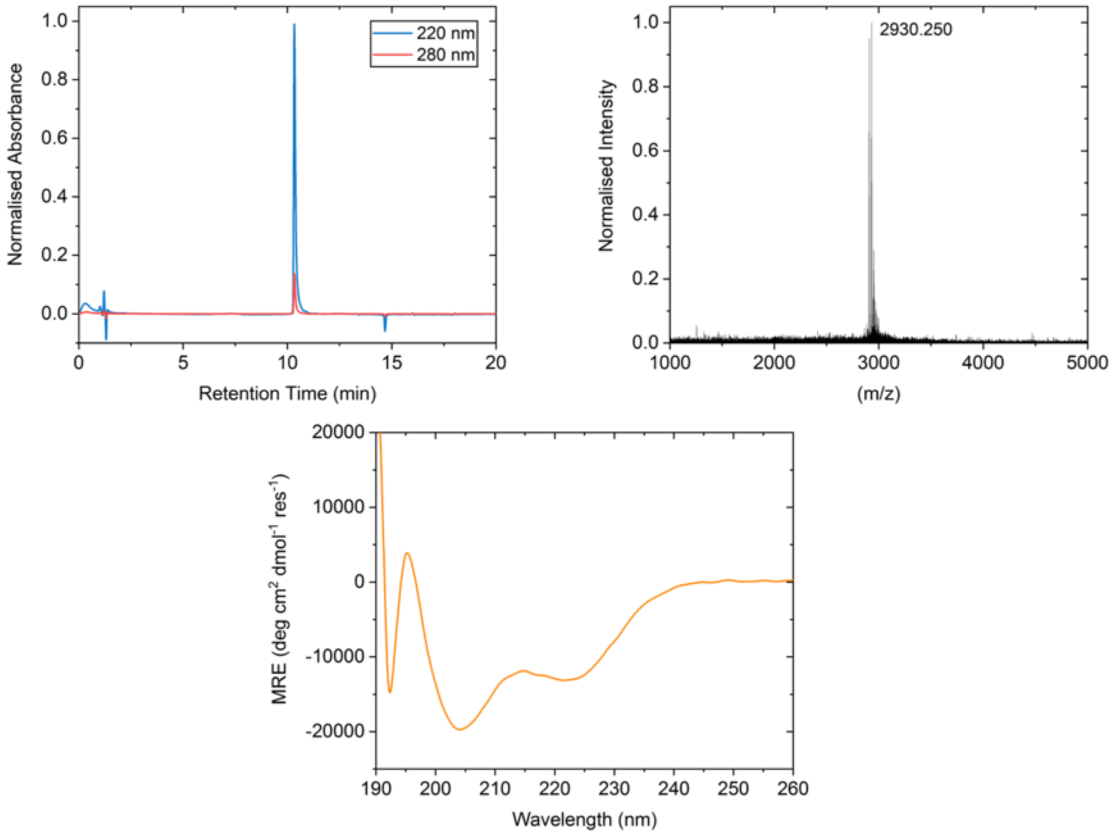

Figure S1: Top left: Analytical HPLC spectrum of pure peptide (gradient: 0–60% Buffer B). Top right: MALDI-TOF mass spectrometry trace ($[M+H]^+$ expected mass = 2930.5 Da, observed mass = 2930.250 Da). Bottom: CD spectrum of peptide in H_2O (conditions: 50 µM, 5 °C, PBS, pH 7.4). CD spectrum colour matches that in Fig. 1C.

CC-Mono-A13a (**9**)

Sequence: `Ac-G EAAAAKQ EAAAaKK EAAAAKW EAAAAKQ G-NH₂`

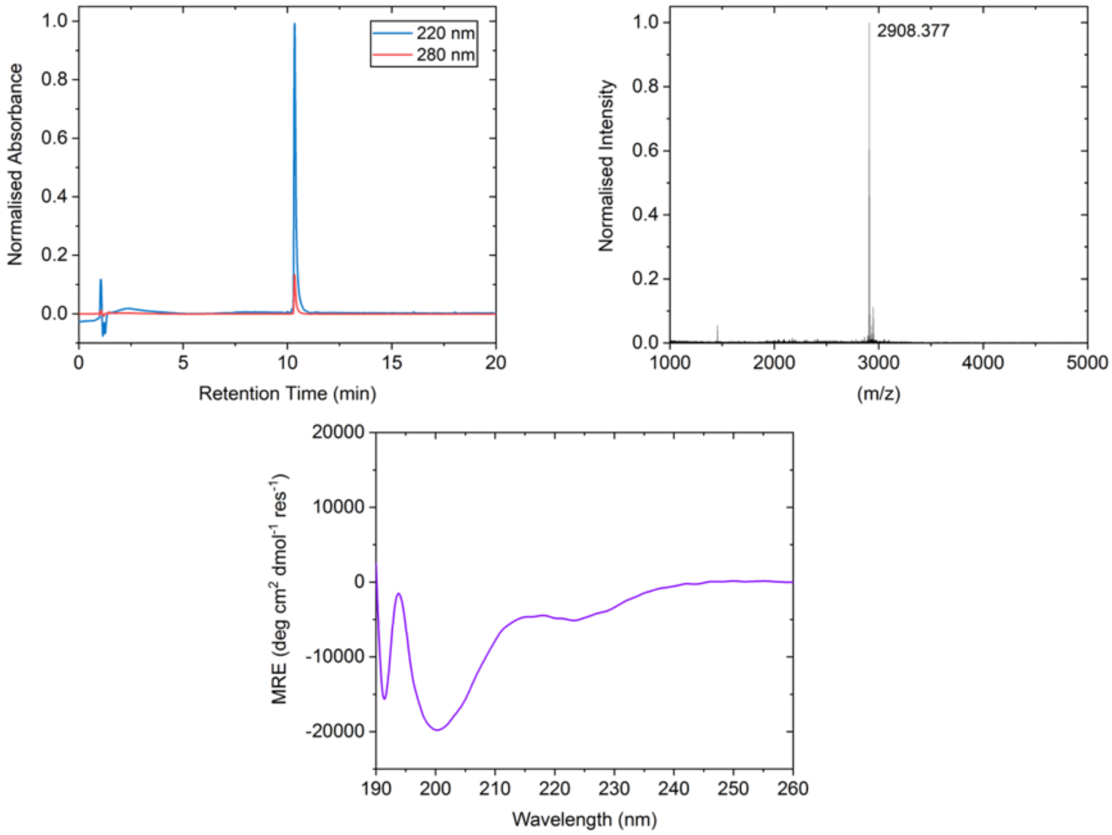

Figure S2: Top left: Analytical HPLC spectrum of pure peptide (gradient: 0–60% Buffer B). Top right: MALDI-TOF mass spectrometry trace ($[M+H]^+$ expected mass = 2908.5 Da, observed mass = 2908.377). Bottom: CD spectrum of peptide in H_2O (conditions: 50 µM, 5 °C, PBS, pH 7.4). CD spectrum colour matches that in Fig. 1C.

CC-Mono-A13ϕ (**10**)

Sequence: `Ac-G EAAAAKQ EAAAϕKK EAAAAKW EAAAAKQ G-NH2`

ϕ = 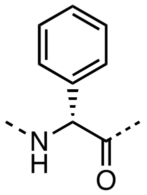

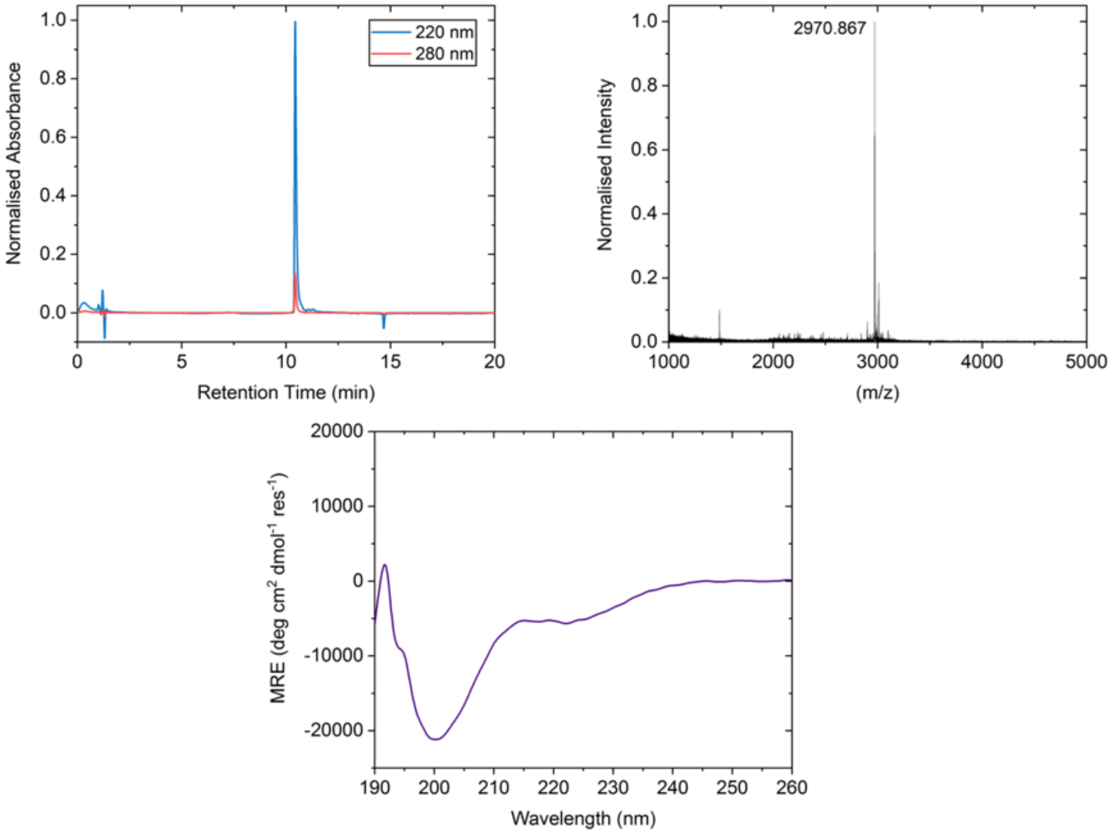

Figure S3: Top left: Analytical HPLC spectrum of pure peptide (gradient: 0–60% Buffer B). Top right: MALDI-TOF mass spectrometry trace ($[M+H]^+$ expected mass = 2970.5 Da, observed mass = 2970.867 Da). Bottom: CD spectrum of peptide in H_2O (conditions: 50 µM, 5 °C, PBS, pH 7.4). CD spectrum colour matches that in Fig. 1C.

CC-Mono-A13Φ (**11**)

Sequence: Ac−G EAAAAKQ EAAAΦKK EAAAAKW EAAAAKQ G−NH_2

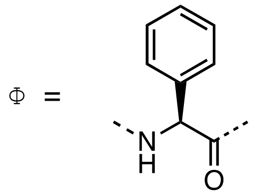

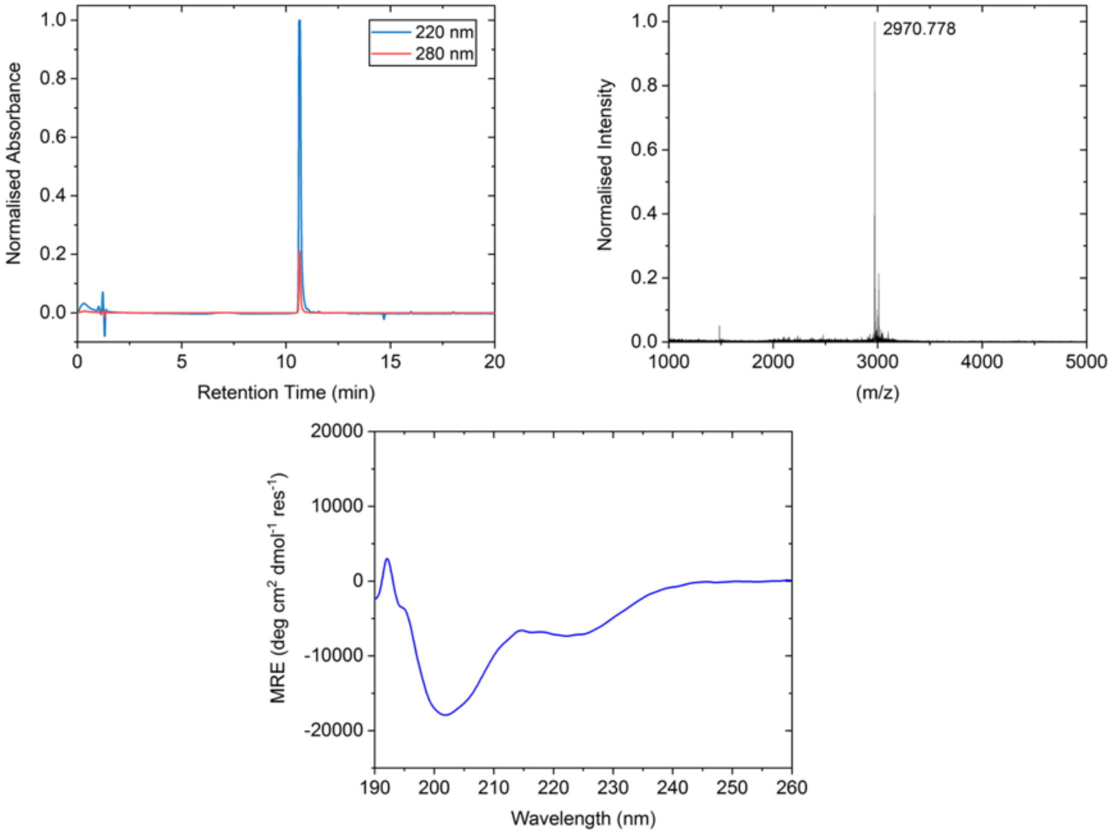

Figure S4: Top left: Analytical HPLC spectrum of pure peptide (gradient: 0–60% Buffer B). Top right: MALDI-TOF mass spectrometry trace ($[M+H]^+$ expected mass = 2970.5 Da, observed mass = 2970.778 Da). Bottom: CD spectrum of peptide in H_2O (conditions: 50 µM, 5 °C, PBS, pH 7.4). CD spectrum colour matches that in Fig. 1C.

CC-Mono-A13b (**12**)

Sequence: `Ac-G EAAAAKQ EAAAbKK EAAAAKW EAAAAKQ G-NH₂`

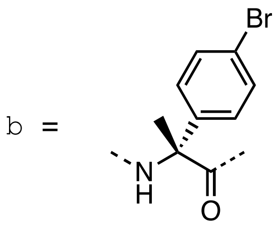

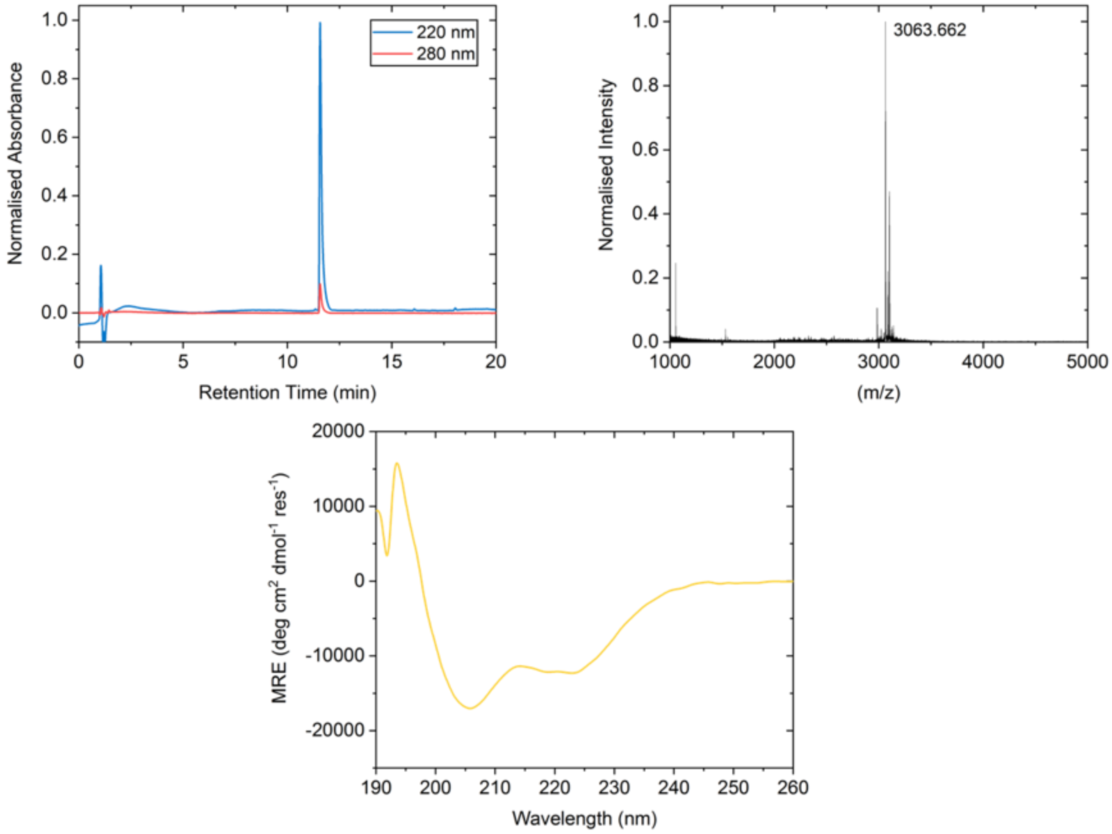

Figure S5: Top left: Analytical HPLC spectrum of pure peptide (gradient: 0–60% Buffer B). Top right: MALDI-TOF mass spectrometry trace ($[M+H]^+$ expected mass = 3063.5 Da, observed mass = 3063.662 Da). Bottom: CD spectrum of peptide in H_2O (conditions: 50 µM, 5 °C, PBS, pH 7.4). CD spectrum colour matches that in Fig. 1C.

CC-Mono-A13B (**13**)

Sequence: `Ac-G EAAAAKQ EAAABKK EAAAAKW EAAAAKQ G-NH2`

B =

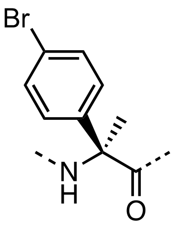

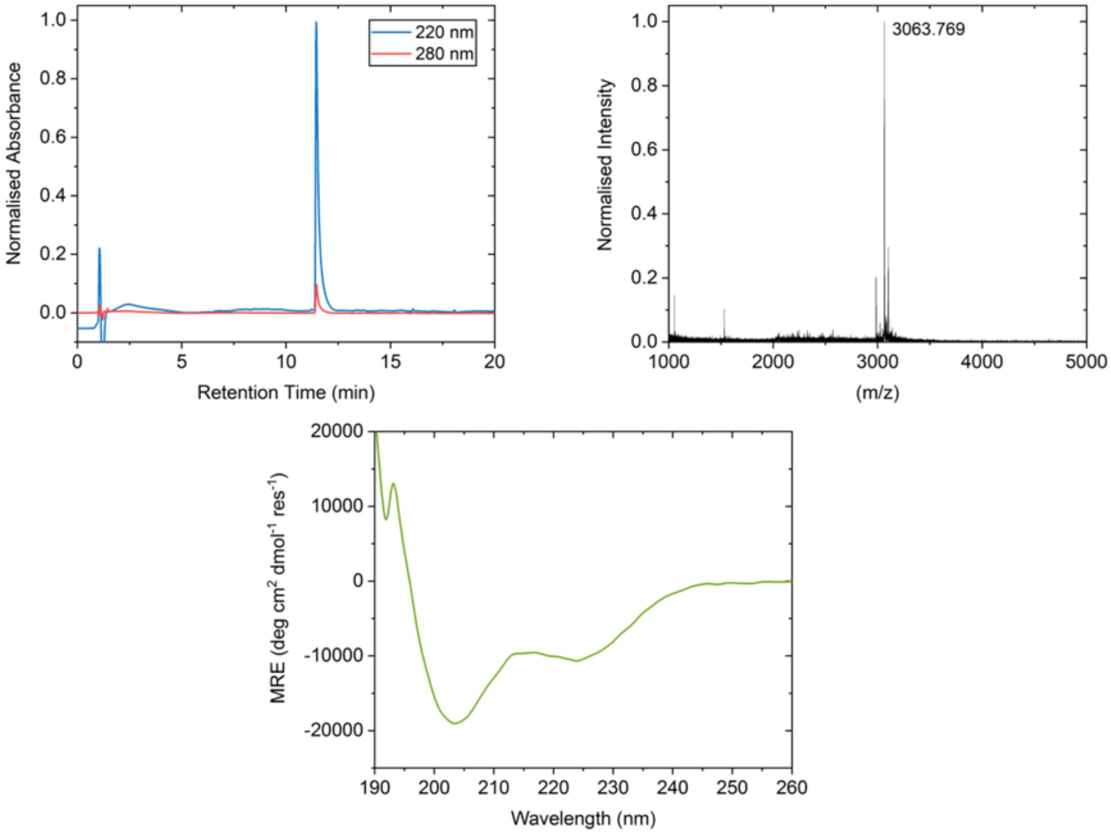

Figure S6: Top left: Analytical HPLC spectrum of pure peptide (gradient: 0–60% Buffer B). Top right: MALDI-TOF mass spectrometry trace ($[M+H]^+$ expected mass = 3063.5 Da, observed mass = 3063.769 Da). Bottom: CD spectrum of peptide in H_2O (conditions: 50 µM, 5 °C, PBS, pH 7.4). CD spectrum colour matches that in Fig. 1C.

CC-Mono-A13U (**14**)

Sequence: `Ac−G EAAAAKQ EAAAUKK EAAAAKW EAAAAKQ G−NH2`

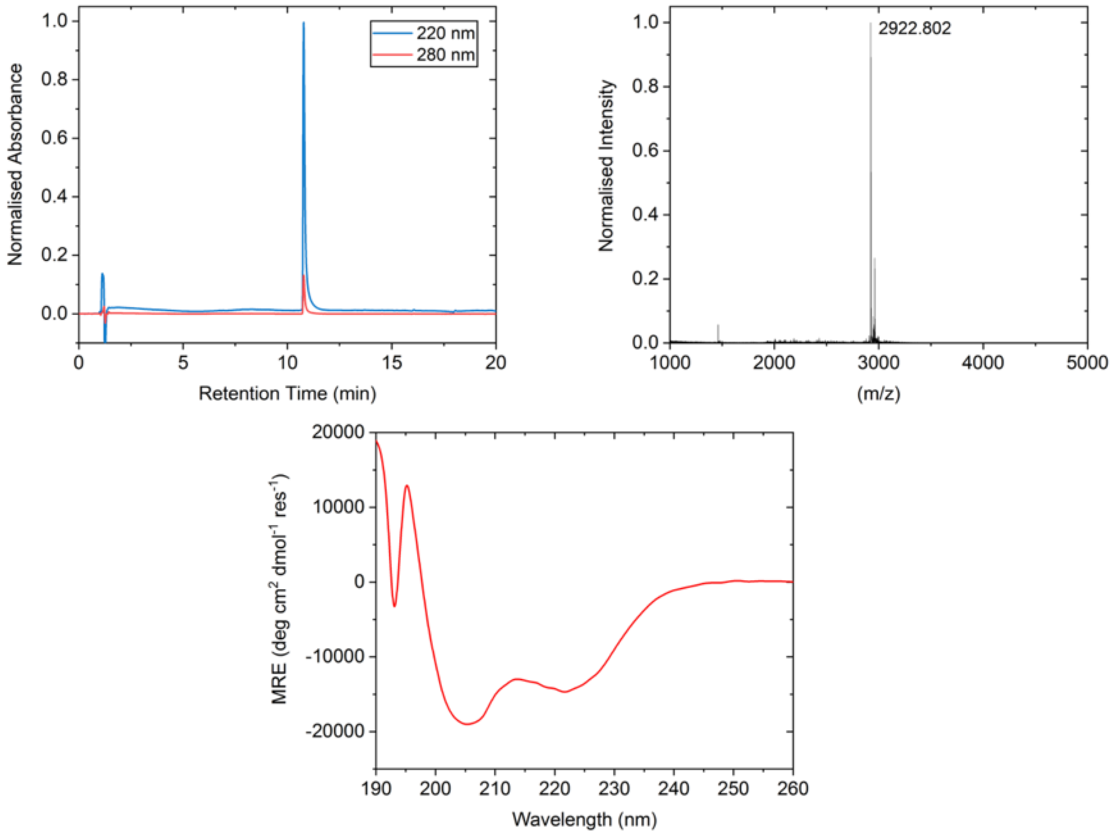

Figure S7: Top left: Analytical HPLC spectrum of pure peptide (gradient: 0–60% Buffer B). Top right: MALDI-TOF mass spectrometry trace ($[M+H]^+$ expected mass = 2922.5 Da, observed mass = 2922.802 Da). Bottom: CD spectrum of peptide in H_2O (conditions: 50 µM, 5 °C, PBS, pH 7.4). CD spectrum colour matches that in Fig. 1C.

CC-Di (**15**)

Sequence: `Ac-G EIAALKQ EIAALKK ENAALKW EIAALKQ GW-NH`$_2$

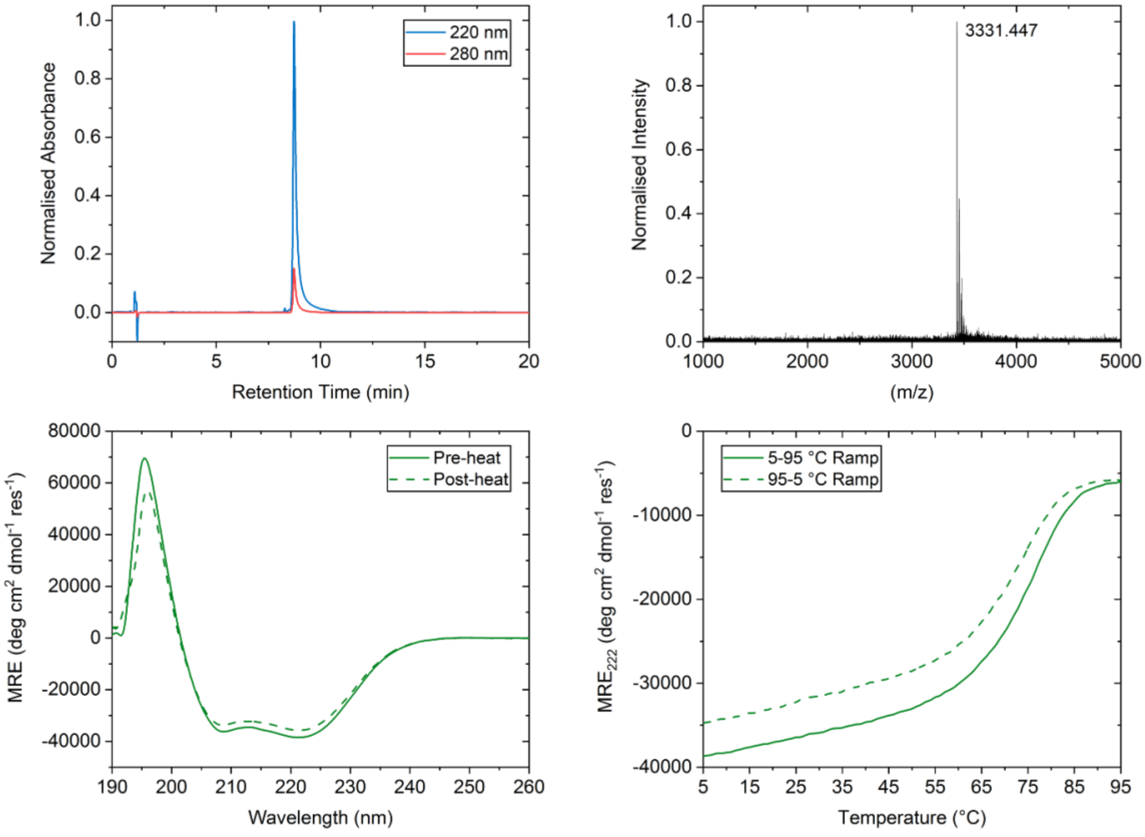

Figure S8: Top left: Analytical HPLC spectrum of pure peptide (gradient: 20–80% Buffer B). Top right: MALDI-TOF mass spectrometry trace ($[M+H]^+$ expected mass = 3431.9 Da, observed mass = 3431.447 Da). Bottom left: CD spectra of peptide in H_2O before and after heating (conditions: 50 µM, 5 °C, PBS, pH 7.4). Bottom right: Thermal denaturation profiles of peptide in H_2O upon heating and cooling between 5 and 95 °C (conditions: 50 µM, PBS, pH 7.4). CD spectra colours matches those in Fig. 1D & E.

CC-Di-W22b (**16**)

Sequence: `Ac-G EIAALKQ EIAALKK ENAALKb EIAALKQ GW-NH`$_2$

b =

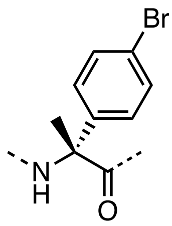

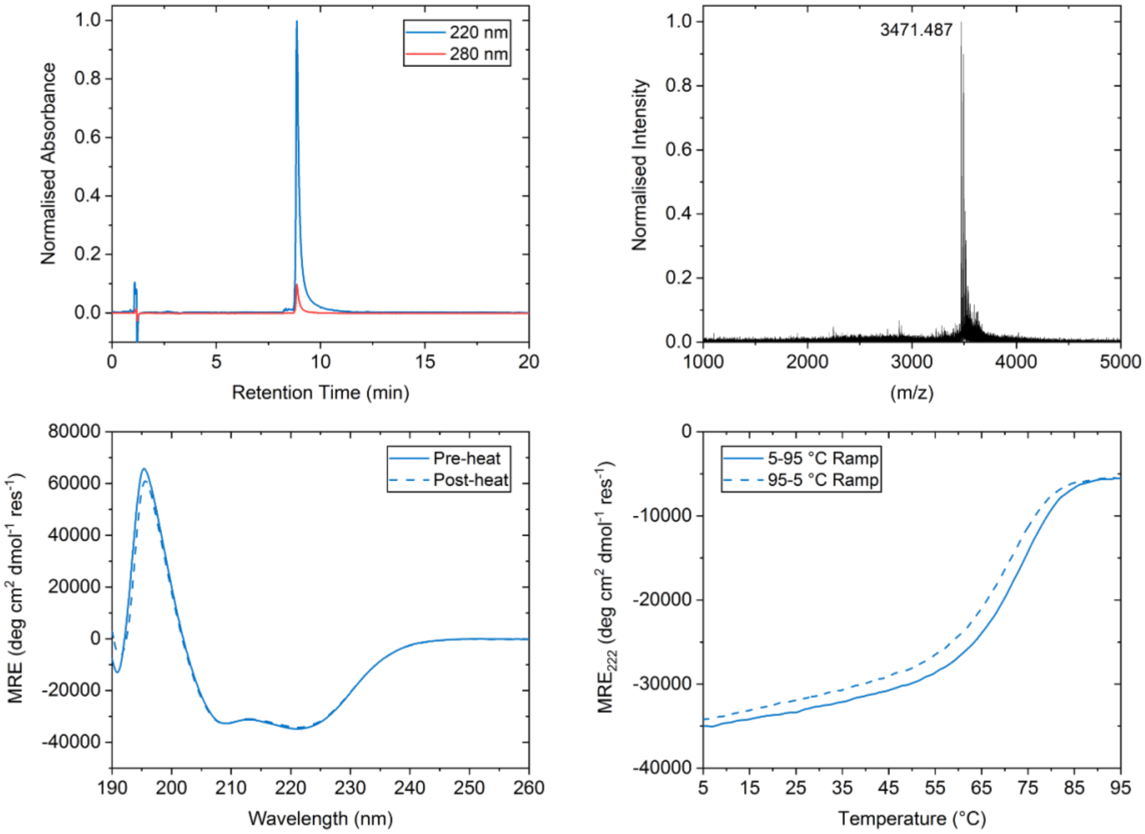

Figure S9: Top left: Analytical HPLC spectrum of pure peptide (gradient: 20–80% Buffer B). Top right: MALDI-TOF mass spectrometry trace ($[M+H]^+$ expected mass = 3470.8 Da, observed mass = 3471.487 Da). Bottom left: CD spectra of peptide in H_2O before and after heating (conditions: 50 µM, 5 °C, PBS, pH 7.4). Bottom right: Thermal denaturation profiles of peptide in H_2O upon heating and cooling between 5 and 95 °C (conditions: 50 µM, PBS, pH 7.4). CD spectra colours matches those in Fig. 1D & E.

CC-Di-W22B (**17**)

Sequence: `Ac-G EIAALKQ EIAALKK ENAALKB EIAALKQ GW-NH`$_2$

B =

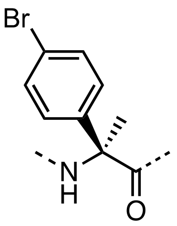

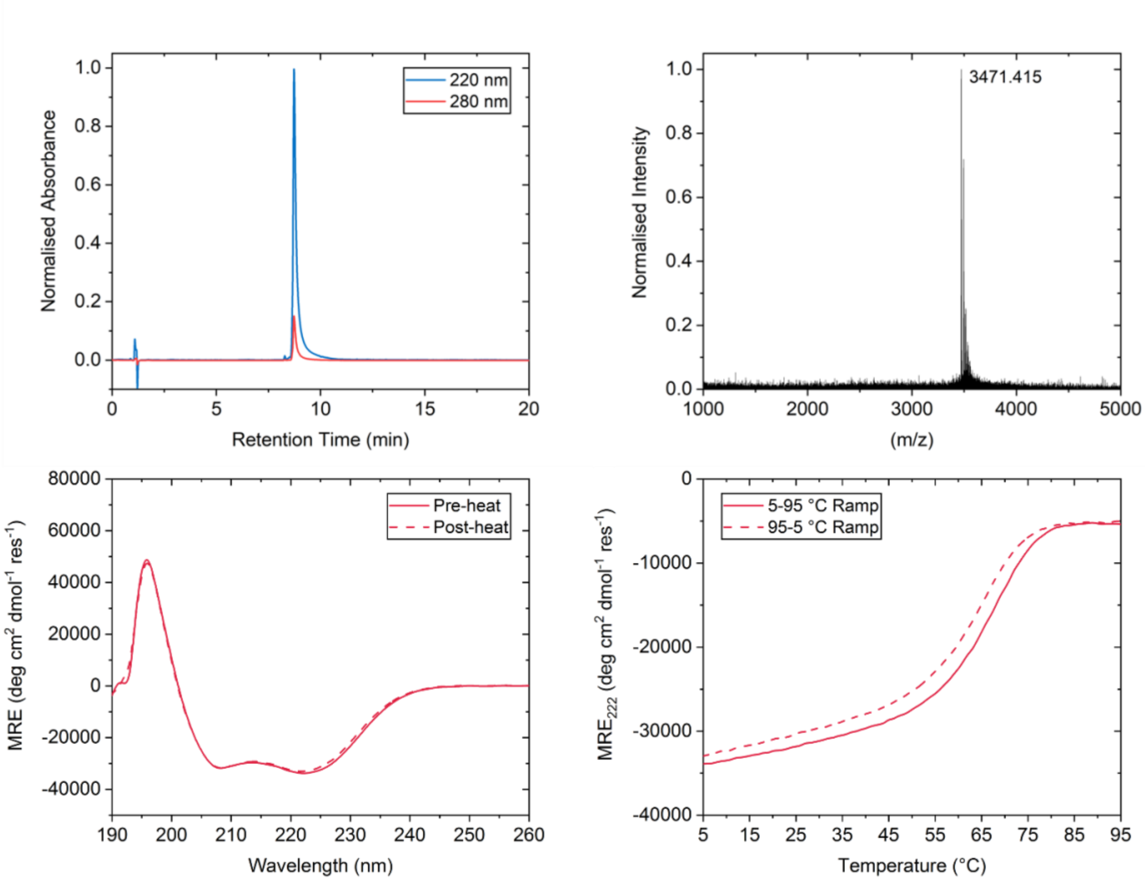

Figure S10: Top left: Analytical HPLC spectrum of pure peptide (gradient: 20–80% Buffer B). Top right: MALDI-TOF mass spectrometry trace ($[M+H]^+$ expected mass = 3470.8 Da, observed mass = 3471.415 Da). Bottom left: CD spectra of peptide in H_2O before and after heating (conditions: 50 µM, 5 °C, PBS, pH 7.4). Bottom right: Thermal denaturation profiles of peptide in H_2O upon heating and cooling between 5 and 95 °C (conditions: 50 µM, PBS, pH 7.4). CD spectra colours matches those in Fig. 1D & E.

Sedimentation Equilibrium Traces

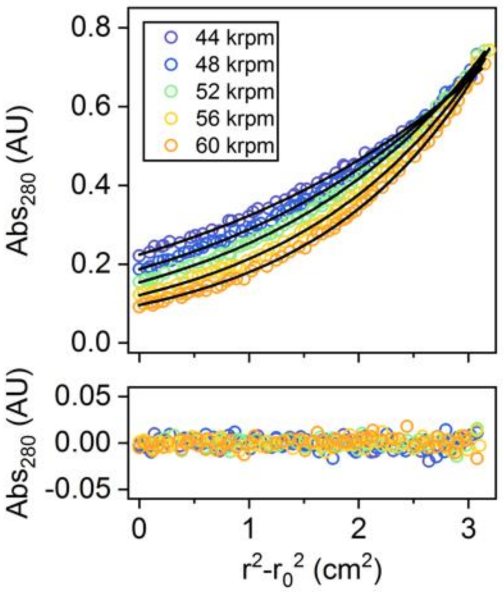

CC-Mono (**8**)

Figure S11: Top: SE data (circles) fitted to a single ideal species model (black lines) at 44, 48, 52, 56 and 60 krpm, returning MW = 3070 Da (1.09 x monomer mass, 95% confidence limits: 3023–3154 Da). Bottom: residuals for the above fit at respective krpm. Measurements were carried out at 75 µM, 20 °C, PBS, pH 7.4.

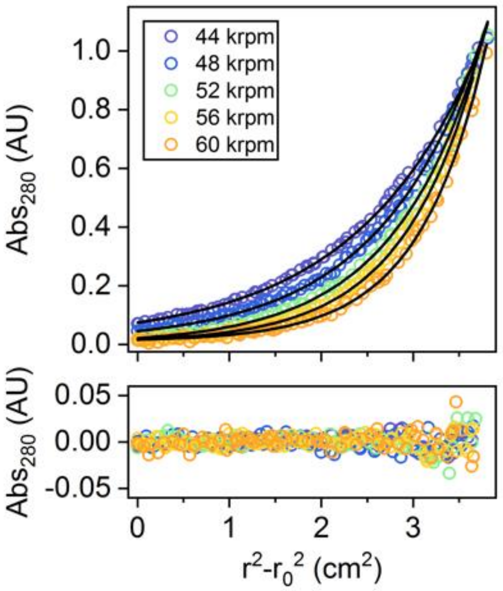

CC-Di (**15**)

Figure S12: Top: SE data (circles) fitted to a single ideal species model (black lines) at 44, 48, 52, 56 and 60 krpm, returning MW = 7321 Da (2.13 x monomer mass, 95% confidence limits: 7272–7445 Da). Bottom: residuals for the above fit at respective krpm. Measurements were carried out at 75 µM, 20 °C, PBS, pH 7.4.

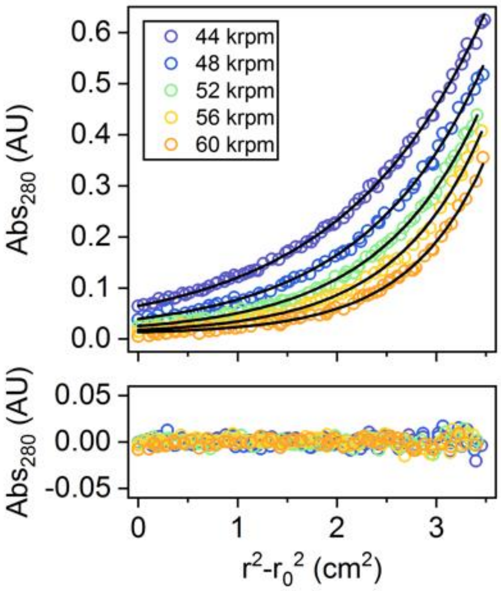

CC-Di-W22b (**16**)

Figure S13: Top: SE data (circles) fitted to a single ideal species model (black lines) at 44, 48, 52, 56 and 60 krpm, returning MW = 6899 Da (1.99 x monomer mass, 95% confidence limits: 6834–6965 Da). Bottom: residuals for the above fit at respective krpm. Measurements were carried out at 75 µM, 20 °C, PBS, pH 7.4.

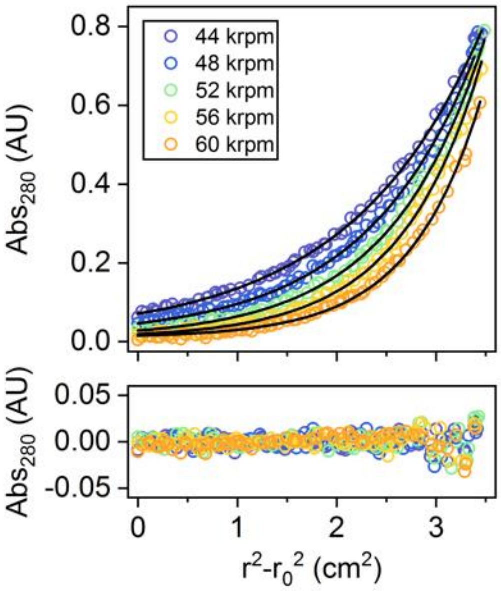

CC-Di-W22B (**17**)

Figure S14: Top: SE data (circles) fitted to a single ideal species model (black lines) at 44, 48, 52, 56 and 60 krpm, returning MW = 7439 Da (2.14 x monomer mass, 95% confidence limits: 7345–7539 Da). Bottom: residuals for the above fit at respective krpm. Measurements were carried out at 37 µM, 20 °C, PBS, pH 7.4.

References

[1] Z. G. Le, Z. C. Chen, Y. Hu, Q. G. Zheng, *Synthesis (Stuttg).* **2004**, *2004*, 2809.

[2] T. Iwai, T. Fujihara, J. Terao, Y. Tsuji, *J. Am. Chem. Soc.* **2010**, *132*, 9602.

[3] D. J. Leonard, J. W. Ward, J. Clayden, *Nature* **2018**, *562*, 105.

[4] M. M. Amer, A. C. Carrasco, D. J. Leonard, J. W. Ward, J. Clayden, *Org. Lett.* **2018**, *20*, 7977.

[5] J. M Scholtz, H. Qian, E. J. York, J. M. Stewart, R. L Baldwin, *Biopolymers* **1992**, *31*, 1463.

[6] J. K. Myers, C. N. Pace, J. M. Scholtz, *Proc. Natl. Acad. Sci. USA* **1997**, *94*, 2833.
